# Cause-specific years of life lost attributable to non-optimal body mass index by county, sex, race, and ethnicity in the USA, 2000–2019: a systematic analysis of health disparities

**DOI:** 10.1186/s12916-026-04795-y

**Published:** 2026-03-18

**Authors:** GBD US Health Disparities Collaborators, Farah Mouhanna, Ethan Kahn, Chris A. Schmidt, Theresa A. McHugh, Mathew M. Baumann, Yekaterina O. Kelly, Wichada La Motte-Kerr, Rebecca M. Cogen, Xiaochen Dai, Emmanuela Gakidou, César Montalvo-Clavijo, Zhuochen Li, Michael Celone, Nicole DeCleene, Kosuke Tamura, Kelvin Choi, Juliana Teruel Camargo, Amanda S. Hinerman, Christian S. Alvarez, George A. Mensah, Eliseo J. Pérez-Stable, Christopher J. L. Murray, Ali H. Mokdad, Laura Dwyer-Lindgren

**Affiliations:** 1 Institute for Health Metrics and Evaluation, University of Washington, Seattle, WA USA; 2 Department of Health Metrics Sciences, University of Washington, Seattle, WA USA; 3 Division of Intramural Research, National Institute On Minority Health and Health Disparities, National Institutes of Health, Bethesda, MD USA; 4 Epidemiology and Community Health Branch, Division of Intramural Research, National Heart, Lung and Blood Institute, National Institutes of Health, Bethesda, MD USA; 5 Center for Translation Research and Implementation Science, National Heart, Lung, and Blood Institute, National Institutes of Health, Bethesda, MD USA; 6 University of California, San Francisco, San Francisco, CA USA

**Keywords:** Body mass index, Obesity, Race and ethnicity, Health disparities, BRFSS, Attributable years of life lost, Small-area estimation, US counties

## Abstract

**Background:**

Over 334,000 deaths in 2021 in the USA were attributed to non-optimal body mass index ([BMI] greater than 20 to 21 kg/m^2^), with elevated mortality among American Indian and Alaska Native (AIAN), Black, and Latino populations. Disparities in attributable mortality by race, ethnicity, and county are poorly understood. This analysis examined variation by race and/or ethnicity in obesity prevalence (BMI ≥ 30) and years of life lost (YLLs) attributable to non-optimal BMI in 3110 US counties from 2000 to 2019.

**Methods:**

Using survey data from the Behavioral Risk Factor Surveillance System (BRFSS), Gallup Daily, and National Health and Nutrition Examination Survey (NHANES), we estimated obesity prevalence annually, stratified by county, age, sex, and five mutually exclusive racial and/or ethnic populations (AIAN, Asian or Pacific Islander [Asian], Black, Latino or Hispanic [Latino], and White). We calculated population attributable fractions (PAFs) and estimated YLLs attributable to non-optimal BMI for 27 causes of death (focusing on ischemic heart disease [IHD], colorectal cancer, and diabetes) using cause-specific YLL estimates from a previous analysis.

**Results:**

Age-standardized obesity prevalence increased by 12.3 percentage points (95% uncertainty interval 11.9–12.8) to 40.2% (40.0–40.6) in the USA from 2000 to 2019 and was highest in the Black population, followed by the AIAN, Latino, White, and Asian populations. In 2019, the Black population had the highest rates of IHD and colorectal cancer YLLs attributable to non-optimal BMI, followed by the AIAN, White, Latino, and Asian populations. The AIAN population had the highest attributable YLL rate for diabetes in 2019, followed by the Black, Latino, White, and Asian populations. All racial and/or ethnic populations had statistically significant reductions in IHD and diabetes YLL rates attributable to non-optimal BMI from 2000 to 2019, with declines in total YLL rates for these causes more than offsetting increases in obesity prevalence and PAFs. Relative disparities among counties were two to four times as large for attributable YLL rates as for obesity prevalence.

**Conclusions:**

Racial and/or ethnic disparities in obesity prevalence are substantial, but disparities in YLLs attributable to non-optimal BMI are larger because they are compounded by disparities in YLL rates.

**Supplementary Information:**

The online version contains supplementary material available at 10.1186/s12916-026-04795-y.

## Background

Non-optimal body mass index ([BMI] greater than 20 to 21 kg/m^2^, which is associated with increased all-cause mortality) [1] is the leading health risk for morbidity and among the top five leading causes of mortality in the USA, contributing to over 334,000 deaths in 2021 [1]. Obesity (BMI ≥ 30) is a risk factor for many adverse health outcomes, including cardiovascular diseases, diabetes, kidney diseases, neoplasms, musculoskeletal disorders, and dementia [1]. In the USA, adult obesity prevalence is high: 44% in 2021, double the prevalence in 1990 [2]. Obesity has increased in nearly every nation, but increased more from 1990 to 2022 in the USA than in any other high-income Western country [3, 4], likely contributing to relatively low and stagnant life expectancy [5]. Additionally, the effects of obesity have a large financial impact. In 2021, Americans with private health insurance and an obesity diagnosis spent more than twice as much on health care as those without an obesity diagnosis [6].

Substantial variation in obesity prevalence has been documented at the state level, and to an even greater degree at the county level. Mississippi, Arkansas, and Louisiana are among the states with the highest obesity prevalence (both extreme [BMI ≥ 40] and moderate [BMI 30.0–39.9]), while Colorado, Massachusetts, and Vermont are among the lowest; yet, hot and cold spots of obesity can occur within the same state [7]. Generally, obesity prevalence is higher in the Southeast and Midwest and lower in the Pacific Northwest and western US—ranging from 17.7% in San Francisco County, California to 53.0% in Holmes County, Mississippi [8]. Previous research examining obesity prevalence at the county level identified demographic, socioeconomic, health care, and environmental factors as having the greatest predictive power [9]; positive associations have also been found between obesity prevalence and unemployment, outpatient health care visits, physical inactivity, female-headed families, and less education [10]. Accurate county-level estimates can provide valuable insights for effectively shaping local policies and interventions to reduce obesity and its health consequences.

While obesity is a widespread health condition in the USA, disparities in prevalence are known to exist across racial and/or ethnic populations. Generally, the Black population has the highest obesity prevalence, followed by the Latino and White populations, while the Asian population has the lowest prevalence [11]. The American Indian or Alaska Native (AIAN) population faces a high prevalence of obesity [12], although results for this population are often aggregated into broader “other” groups or not reported due to small sample sizes [13]. Consideration of racial and/or ethnic disparities is essential for understanding geographic variation in obesity prevalence [10].

Prior research has reported state-level estimates of cause, age, and sex-specific mortality and morbidity attributable to non-optimal BMI [1], and of excess all-cause mortality attributable to non-optimal BMI for White, Black, Latino, and total populations [14]. While these state-level studies are rigorous and account for self-report bias, they lack the geographic granularity needed to identify local determinants of health, especially as those differ by race and/or ethnicity. Existing county-level analyses of obesity prevalence typically have not included corrections for self-report bias (the most recent example that did correct for self-report bias is more than a decade old) [15], and none to our knowledge has estimated attributable mortality for a full set of causes and locations. Our study fills these gaps by comprehensively analyzing racial and/or ethnic disparities in premature mortality associated with non-optimal BMI across US counties among adults aged 20 years and older (hereafter, “adults”) from 2000 to 2019. We provided county-level estimates by sex and race and/or ethnicity for obesity prevalence and years of life lost (YLLs) attributable to non-optimal BMI for 27 causes of death. The combination of geographic granularity, methodological rigor, and demographic detail enables more precise identification of communities with elevated risk.

## Methods

### Overview of approach

Our statistical analysis leveraged survey data from the Behavioral Risk Factor Surveillance System (BRFSS) [16], Gallup Daily [17], and National Health and Nutrition Examination Survey (NHANES) [18], and had three stages, which we summarize here and describe in more detail in the following sections. First, BMI estimates (calculated from self-reported height and weight) were adjusted to correct for self-reporting bias. Second, we used small-area estimation (SAE) models to estimate the prevalence of obesity (BMI ≥ 30) among adults aged 20 years and older by year, county, age group, sex, and racial and/or ethnic population, hereafter referred to as a “stratum.” We then estimated the population distributions of BMI at this same granularity. Third, we calculated population attributable fractions (PAFs, the proportion of the disease avoidable by reducing risk exposure to a theoretical minimum) for all strata and combined them with published YLL estimates [19] to calculate YLLs attributable to non-optimal BMI separately by cause of death.

This study adheres to the Guidelines for Accurate and Transparent Reporting of Health Estimates (Additional file 1: Sect. 1) [20]. Institutional review board approval was obtained from the University of Washington. No primary data were collected for this study, and we had no contact with human participants.

We estimated attributable YLLs for 27 causes of death that were directly linked to non-optimal BMI in the Global Burden of Diseases, Injuries, and Risk Factors Study (GBD) 2021 [1], and which had at least 10,000 total US deaths from 2000 to 2019 (Additional file 1: Table S1) [1, 19]. We used the same geographic units and racial and/or ethnic classification system as a previous study on cause-specific mortality by county and race and/or ethnicity [19], which provided critical inputs to this analysis. Spatial units consisted of counties or equivalents that were merged, if necessary, to create stable boundaries over time [19]. We refer to these 3110 counties or merged-county units as “counties” (collectively encompassing all 3143 counties or equivalents existing in 2019; Additional file 1: Table S2). We present a stratified analysis of obesity and attributable YLL rates in counties with and without federally recognized tribal reservations, defining the former as those with at least 1% overlap in their land area with federal reservations (Additional file 1: Table S3).

We used a combined racial and/or ethnic classification (“race and/or ethnicity”) with five mutually exclusive populations: AIAN, Asian or Pacific Islander (Asian), Black, Latino or Hispanic of any race (Latino), and White. This is consistent with the 1977 version of the Office of Management and Budget standards for federal data collection on race and/or ethnicity [21]. While a 1997 update [22] to these standards separated Asian and Native Hawaiian and Pacific Islander (NHPI) populations and required data collectors to provide the option to select multiple races, this analysis follows the older classification system due to constraints in the data underlying the YLL estimates used in this study [19]. Specifically, the updated Office of Management and Budget guidance was not implemented in death certificates by all states until mid-2017 [23]. Additionally, a combined “Other Asian and Pacific Islander” group was reported prior to 2011, which prevented full disaggregation of Asian and NHPI individuals. We refer to this combined population as “Asian,” as the Asian population in the USA is an order of magnitude larger than the NHPI population [24], and our estimates are therefore generally reflective of the Asian population. We also did not model health outcomes for a “Two or More Races” category, instead assigning individuals to self-reported or imputed “primary” racial and/or ethnic populations, where available (Additional file 1: Sect. 2.1) [1, 16–19, 21, 25–30].

### Self-report bias correction

We developed a quantile-based model to adjust self-reported BMI in the BRFSS (*N* = 6,902,216; 1999–2020) and Gallup Daily (*N* = 2,245,371; 2008–2017) surveys, leveraging information about self-reported and measured BMI among NHANES respondents (*N* = 56,981; 1999–2023) (Additional file 1: Sects. 2.1–2.3) [28, 31–45]. We accounted for differences in self-report bias by age, sex, race and/or ethnicity, and quantile of self-reported BMI by stratifying the models by sex and including parameters for age group, race and/or ethnicity, and BMI quantile (Additional file 1: Sect. 2.3). We predicted ten sets of adjusted BMI values for all BRFSS and Gallup respondents and propagated uncertainty in the self-report correction to all subsequent estimation steps.

### Obesity prevalence

Next, we used SAE models to estimate obesity prevalence (BMI ≥ 30) for all strata (Additional file 1: Sect. 2.4) [43, 44, 46–54]. We separately modeled the prevalence of BMI ≥ 25 and the proportion of people with obesity among those with BMI ≥ 25. We multiplied these estimates to obtain obesity prevalence (Additional file 1: Sect. 2.4). These models were fit on self-report adjusted data from BRFSS and Gallup and included three county-level covariates: poverty rate and percentage born outside the USA, both by race and/or ethnicity, and population density (Additional file 1: Sect. 2.5) [43, 54–58]. Models were fit using the Template Model Builder package [57] in R. We used multilevel regression and post-stratification to adjust for sampling and non-response bias across data sources (Additional file 1: Sect. 2.4) [54]. We fit separate SAE models for each set of predicted self-report-adjusted BMI, producing 100 draws per model (1000 draws in total) for each stratum.

### YLLs attributable to non-optimal BMI

In order to calculate YLLs (a measure of premature death) attributable to non-optimal BMI, we estimated for each stratum the full continuous population distribution of BMI, optimized to reflect the estimated prevalence of BMI ≥ 25 and the prevalence of obesity (Additional file 1: Sect. 2.6) [1, 42, 59]. We used continuous BMI distributions and relative risk curves to account for the gradient of risk across BMI levels, including within standard BMI categories. We then calculated PAFs for 27 causes of death using continuous global cause-specific relative risk curves and the theoretical minimum risk exposure level (TMREL) (Additional file 1: Sect. 2.7) [1]. The TMREL (BMI 20–21 kg/m^2^) is the empirically derived BMI level associated with the lowest risk of all-cause mortality and does not rely on the “normal” BMI category of 18.5–24.9. We utilized the TMREL estimated in GBD 2021, which was the BMI value that minimized the mortality-weighted mean of cause-specific relative risk (Additional file 1: Sect. 2.7) [1, 60]. The PAFs and associated morbidity and mortality attributable to “non-optimal BMI” represent excess YLLs that would have been averted if the population distribution of BMI above the TMREL was reduced to the TMREL. We multiplied cause-specific PAF estimates by corresponding YLL estimates from a previous study [19] to derive 1000 draws of YLLs attributable to non-optimal BMI for all strata.

We derived final point estimates from the means of the 1000 draws, and 95% uncertainty intervals (UIs) from their 2.5th and 97.5th percentiles. We generated estimates for males and females combined, all racial and/or ethnic populations combined, and state and national levels by population-weighting age-specific estimates, and age-standardized estimates to the US adult population age structure in the 2010 Census. We described differences between any pair of estimates as statistically significant when the posterior probability that the difference is greater than 0 was less than 2.5% or greater than 97.5%, akin to a two-tailed test with an alpha of 0.05. We calculated the coefficient of variation (CoV; the standard deviation divided by the mean) to compare geographical variation across different outcomes, where higher values indicate greater relative variation. Finally, we masked (i.e., did not display) estimates for strata with mean annual populations below 1000, as a previous analysis indicated poor performance below this threshold in the model that generated the mortality estimates (Additional file 1: Table S7) [47].

### Role of the funding source

Co-authors employed by the US National Institutes of Health contributed to data interpretation and revising drafts of this report. Otherwise, the funders had no role in study design, data collection, data analysis, or the initial writing of the report.

## Results

### Obesity prevalence

**Fig. 1:**
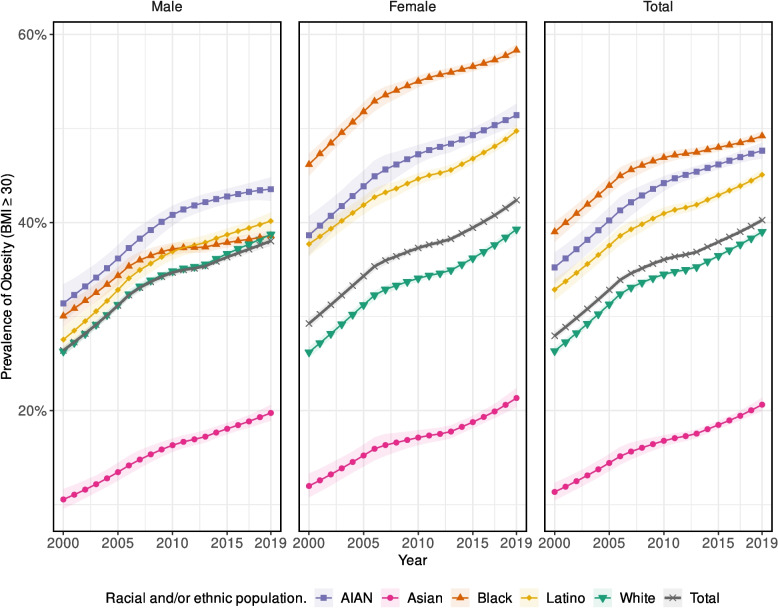
Estimated prevalence of obesity (BMI ≥ 30) in the USA, 2000–2019, by year, sex, and racial and/or ethnic population. Shaded areas indicate the 95% uncertainty intervals

Age-standardized obesity prevalence increased in the USA from 2000 to 2019 by 12.3 percentage points (95% UI 11.9–12.8), from 28.0% (27.5–28.4) to 40.2% (40.0–40.6). Prevalence was highest every year in the Black population (49.2% [48.7–49.7] in 2019), followed by the AIAN (47.6% [46.8–48.5]), Latino (45.1% [44.5–45.6]), White (39.0% [38.6–39.4]), and Asian (20.6% [19.9–21.4]) populations (Fig. 1). The largest absolute increase in obesity prevalence was observed in the White population (12.7 percentage points [12.2–13.2]), followed by the AIAN (12.4 percentage points [10.7–14.4]), Latino (12.2 percentage points [11.1–13.3]), Black (10.2 percentage points [9.1–11.2]), and Asian (9.3 percentage points [8.2–10.3]) populations.

Age-standardized obesity prevalence in the total population was lower and increased by a smaller amount from 2000 to 2019 among males (11.6 percentage points [95% UI 11.0–12.2] to 38.0% [37.5–38.4] in 2019) than females (13.1 percentage points [12.5–13.7] to 42.4 [41.9–42.8]). The White population had the largest increase in prevalence nationally from 2000 to 2019 among females (13.1 percentage points [12.3–13.7]), while the Latino population had the largest increase among males (12.6 percentage points [11.2–14.1]). The Black population had the highest obesity prevalence among females every year from 2000 to 2019 (58.3% [57.7–58.9] in 2019); the AIAN population had the highest prevalence among males in each year (43.5% [42.3–44.8] in 2019).

**Fig. 2:**
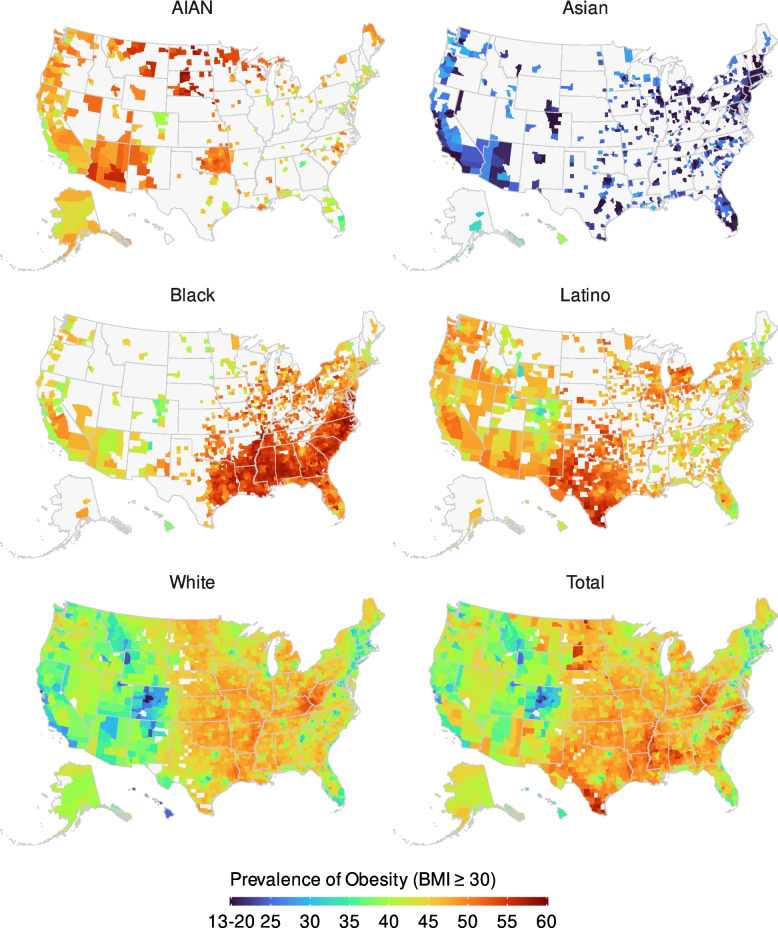
Estimated prevalence of obesity (BMI ≥ 30) in 2019 by county and racial and/or ethnic population. Estimates have been masked for county and racial and/or ethnic populations with a mean annual population fewer than 1000 people

Racial and/or ethnic disparities within counties mostly had ordering consistent with national estimates (Fig. 2). In 2019, the Black population had the highest obesity prevalence nationally and in 79.6% of counties (1183 of 1486; statistically significant in 60.6% [900]), among racial and/or ethnic populations with unmasked estimates in each county. Within the Black population, the prevalence was highest in the Southeast. The Asian population had the lowest obesity prevalence nationally and in 99.4% of counties (665 of 669; statistically significant in 99.0% [662]) but had higher obesity prevalence than the White population (typically having the next lowest prevalence) in 0.6% of counties (4 of 669), all of which were in Hawaii and represented the totality of that state.

### YLLs attributable to non-optimal BMI

Our reporting focuses on causes of death with the largest morbidity and mortality attributable to non-optimal BMI within three different disease categories: ischemic heart disease (IHD), diabetes mellitus, and colorectal cancer. Results for 27 causes of death and 12 aggregate causes are reported in the supplementary materials (Additional file 1: Sect. 5) and online (link available upon publication). We present estimates as age-standardized YLLs per 100,000 population.

**Fig. 3:**
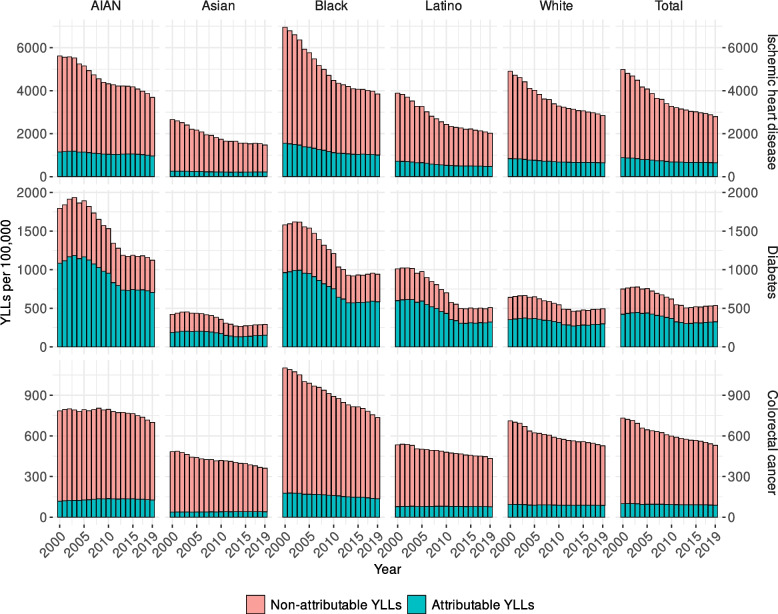
Estimated YLLs (per 100,000 population) attributable to non-optimal BMI and non-attributable to non-optimal BMI in USA, 2000–2019, by year and racial and/or ethnic population for ischemic heart disease, diabetes, and colorectal cancer

The morbidity and mortality attributable to non-optimal BMI in 2019 for IHD was 642 (95% UI 309–951) YLLs per 100,000 population, representing 23% (11–34%) of total YLLs for that cause (Fig. 3). The corresponding morbidity and mortality attributable to non-optimal BMI for diabetes was 326 YLLs per 100,000 (170–419; 61% [32–79%] of total) and for colorectal cancer was 89 YLLs per 100,000 (45–130; 17% [9–25%] of total). The Black population had the highest rates of IHD YLLs attributable to non-optimal BMI in 2019 (1006 [490–1469] per 100,000), followed closely by the AIAN population (963 [472–1436]), and then the White (644 [310–957]), Latino (472 [225–703]), and Asian (216 [100–331]) populations. Colorectal cancer YLLs attributable to non-optimal BMI in 2019 had this same ordering of racial and/or ethnic populations, although the magnitude of the burden was smaller. In 2019, the Black population had 136 (69–196) colorectal cancer YLLs per 100,000 attributable to non-optimal BMI, followed by the AIAN (127 [65–189]), White (88 [44–127]), Latino (77 [39–111]), and Asian (40 [20–61]) populations. The AIAN population had the highest rate of morbidity and mortality attributable to non-optimal BMI in 2019 for diabetes (707 [404–928] YLLs per 100,000), followed by the Black (585 [315–742]), Latino (322 [167–415]), White (299 [155–385]), and Asian (152 [70–211]) populations. Despite having lower obesity prevalence, males had higher attributable YLL rates than females for IHD, diabetes, and colorectal cancer for each racial and/or ethnic population. Compared to racial and/or ethnic disparities in obesity prevalence, relative disparities in YLLs attributable to non-optimal BMI were larger and sometimes ordered differently. For example, the Black male population had lower or equal obesity prevalence to the Latino and White male populations, respectively, but experienced higher rates of IHD YLLs attributable to non-optimal BMI. The ratio of obesity prevalence between the Black and Asian populations, which had the highest and lowest prevalence, respectively, was 2.4 (2.3–2.5) in 2019. The analogous ratio of IHD YLLs attributable to non-optimal BMI was twice as large (4.7 [4.4–5.0]).

Despite increasing obesity prevalence and thus increasing fraction of YLLs attributable to non-optimal BMI from 2000 to 2019, age-standardized rates of YLLs attributable to non-optimal BMI for IHD, diabetes, and colorectal cancer decreased overall and for most racial and/or ethnic populations. IHD had the largest absolute reduction for the total population (by 239 [95% UI 107–375] YLLs per 100,000 population, to 642 [309–951] in 2019), followed by diabetes (by 98 [39–146], to 326 [170–419]) and colorectal cancer (by 10 [5–16], to 89 [45–130]). IHD and diabetes YLL rates attributable to non-optimal BMI declined in all racial and/or ethnic populations from 2000 to 2019, with the largest rate reductions for IHD in the Black population (543 [249–821] per 100,000) and for diabetes in the Black (377 [185–507] per 100,000) and AIAN (377 [158–557] per 100,000) populations. The Black and White populations had slight reductions (42 [21–63] and 6 [2–11] per 100,000, respectively) in colorectal cancer YLL rates attributable to non-optimal BMI, and the Asian population had a small increase (3 [0–7]); the Latino and AIAN populations had no statistically significant changes (− 1 [− 5 to 2] and 9 [0–20], respectively) in morbidity and mortality attributable to non-optimal BMI.

**Fig. 4:**
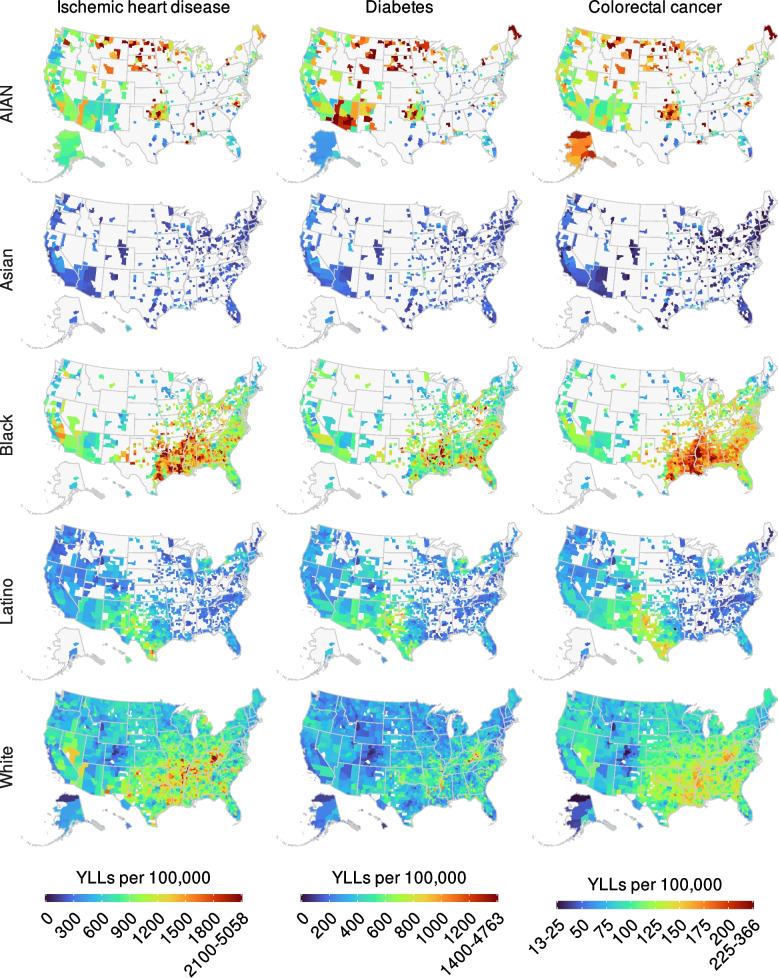
Estimated YLLs (per 100,000 population) attributed to non-optimal BMI in 2019 by county, cause of death, and racial and/or ethnic population. Estimates have been masked for county and racial and/or ethnic populations with a mean annual population fewer than 1000 people

**Fig. 5:**
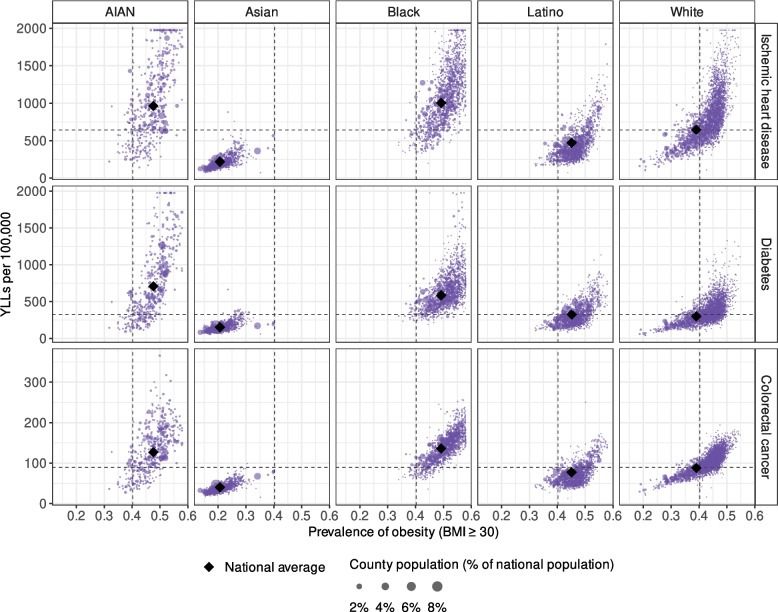
Comparison of county-level prevalence of obesity (BMI ≥ 30) to YLLs (per 100,000 population) attributable to non-optimal BMI in 2019 by cause of death and racial and/or ethnic population. Each circle represents a county, with its size reflecting the county population (% of national population). The diamonds indicate national obesity prevalence and attributable YLL rates for each racial and/or ethnic population. The vertical line marks the overall obesity prevalence, and the horizontal line represents the overall attributable YLL rates. Estimates above the 99.5th percentile are truncated. Estimates have been masked for county and racial and/or ethnic populations with a mean annual population fewer than 1000 people.

Geographical disparities in 2019 were more pronounced for morbidity and mortality attributable to non-optimal BMI than for obesity, despite similar regional patterns (Figs. 2, 4, and 5). The CoV among counties for the total population was 0.11 for obesity, compared with 0.38 for IHD, 0.43 for diabetes, and 0.25 for colorectal cancer. In 2019, the AIAN population had particularly high obesity prevalence and YLL rates for IHD, diabetes, and colorectal cancer attributable to non-optimal BMI (in the top 10% of counties for the same population) in Oklahoma and the northern Midwest. The same was true for the Asian population in the California Central Valley; the Black population in the Southeast, especially the Mississippi Delta region in Mississippi and Arkansas; the Latino population in the Southwest, especially southern and western Texas; and the White population in eastern Kentucky, West Virginia, and eastern Arkansas. For most racial and/or ethnic populations, counties in the bottom decile of obesity prevalence and rates of morbidity and mortality attributable to non-optimal BMI were predominantly in Rocky Mountain states (especially Colorado), mid-Atlantic states, New England, and coastal California.

National statistics obscured disparities in 2019 among counties. For example, the AIAN population had the highest spatial variation in attributable YLL rates (measured by CoV) for all three focal causes, with an interquartile range (IQR) of county-level diabetes YLLs attributable to non-optimal BMI of 347–976 per 100,000 population (median 615). Notably, the AIAN population tended to have higher attributable YLL rates for diabetes in counties with federally recognized tribal reservations (median 978 [IQR 673–1338] per 100,000) than in counties without reservations (median 496 [IQR 303–766]). In comparison, the median obesity prevalence among the AIAN population in counties with reservations was only slightly higher (51% [48%–54%]) than in counties without reservations (46% [43%–49%]).

## Discussion

We found large geographical, sex, and racial and/or ethnic disparities in obesity prevalence and YLL rates attributable to non-optimal BMI. Disparities were substantially greater for attributable YLLs than for obesity prevalence. Obesity prevalence increased from 2000 to 2019, while YLL rates attributable to non-optimal BMI generally decreased. Counties with relatively high obesity prevalence and YLL rates attributable to non-optimal BMI were identified for each racial and/or ethnic population and substantially overlapped for each health outcome. However, the disparities between counties were markedly larger for YLLs attributable to non-optimal BMI than for obesity, highlighting the need for dual efforts to reduce the prevalence of obesity and associated diseases to eliminate geographical and racial and/or ethnic disparities.

Obesity is a complex public health challenge driven by interconnected individual, interpersonal, community, and societal factors [61–63]. The generally high and increasing rates of obesity in the USA are primarily the result of systemic social, economic, and environmental factors [61, 64, 65]. Ample evidence exists linking obesity [66, 67] and chronic disease risk, including IHD, diabetes, and cancer [68, 69] to a number of modifiable structural and behavioral factors—“social determinants of obesity”—that vary among geographic and socio-demographic populations. These factors include access to medical care, food insecurity, safe and accessible outdoor spaces, public transportation, and health behaviors such as diet, physical activity, and sleep duration. Consistent with this existing body of research, these social determinants are a plausible explanation of many geographic and demographic trends observed in this paper. However, perhaps more importantly, our findings expand beyond prior research by emphasizing the need to simultaneously reduce obesity and obesity-related mortality by addressing these factors in the identified populations with the highest prevalence of obesity and the highest YLLs attributable to non-optimal BMI.

Our findings were consistent with previously observed trends of females generally having higher obesity prevalence than males [70] with the largest sex differences in the Black population and the smallest in the White and Asian populations [71]. Moreover, Black females had the highest obesity prevalence nationally among all sex and racial and/or ethnic combinations [66, 67]. Low socioeconomic status [72] and poor physical neighborhood conditions [73] are particularly associated with obesity among females compared with males, regardless of race and/or ethnicity, potentially due to different stress-coping mechanisms, familial responsibilities, and occupational roles, among other factors [71]. One study found equally large sex differences in obesity prevalence between low-income Black and White adults, suggesting that obesity is higher among low-income females than males regardless of their race [71]. Thus, the very high obesity prevalence among Black women and the much larger sex difference in obesity prevalence for the Black as compared to the White population in this study may reflect the much higher exposure to low socioeconomic status among Black (and also AIAN and Latino) populations compared to White populations nationally [71, 74]. Moreover, Black females are more likely than their White counterparts to have high weight gain during pregnancy and to retain this weight postpartum [75], and are also more likely to experience postmenopausal weight gain [76]. These sex-specific mechanisms likely contributed to the much larger racial and/or ethnic disparities in obesity prevalence observed for females compared with males.

Rates of YLLs attributable to non-optimal BMI for IHD, diabetes, and colorectal cancer decreased between 2000 and 2019 overall and in most racial and/or ethnic populations, despite increasing obesity prevalence. These reductions in attributable YLL rates are partially credited to factors that reduce the incidence or lethality of conditions associated with obesity. These factors include improved control of non-BMI risk factors and comorbid conditions (e.g., lower smoking rates, better control of high blood pressure, and reduction in low-density lipoprotein cholesterol levels) and use of thrombolysis for cardiovascular disease [77]; improved screening and early detection, secondary prevention, and improved treatment for colorectal cancer [78]; broader screening and diagnosis of diabetes [79]; and more effective medications for diabetes [80, 81]. Other factors contributing to the divergent trends of obesity and mortality merit further investigation. Concerningly, reductions in mortality from IHD, diabetes, and colorectal cancer have recently stalled [19, 77], while obesity prevalence increased consistently between 2000 and 2019. The prevalence of obesity in the USA is forecasted to increase substantially for adults and adolescents between 2022 and 2050, despite some evidence that the rate of increase has diminished [2]. Notwithstanding reductions in mortality over the past decades, IHD, colorectal cancer, and diabetes were the first, eleventh, and twelfth leading causes of premature death in the USA for adults in 2019, respectively, together costing Americans over 11 million years of life in 2019 [82]. Stagnating mortality rates, compounded by aging and further increases in obesity prevalence, will likely increase rates of YLLs attributable to non-optimal BMI in the future.

Primary prevention strategies may reduce obesity prevalence and YLL rates attributed to non-optimal BMI, but improved treatment for obesity and obesity-related diseases will be crucial to eliminate disparities in attributable YLLs identified in this study. Many social determinants of obesity, such as access to health care, healthy and affordable food, and safe places for physical activity, depend on location [83, 84]. Increasing exposure to these protective factors may also reduce the risk of developing diseases such as diabetes and IHD that share common causes with obesity (e.g., consumption of ultra-processed food and sugar-sweetened beverages) [84–87]. However, prevention efforts alone are insufficient to reduce disparities in YLLs attributable to non-optimal BMI that result from unequal disease management by race and/or ethnicity. For example, Black and Latino populations have significantly lower rates of blood pressure control [88] and glycemic control for those diagnosed with diabetes [89, 90] compared with the White population.

We found greater geographical differences in morbidity and mortality attributable to non-optimal BMI than in obesity alone. Variation in obesity prevalence is a proximate driver of disparities in associated disease prevalence, but the loose correlation between obesity and attributable YLLs suggests that disease-modifying factors play a major role in mortality disparities. Counties with similar obesity levels varied in their attributable YLL rates, underscoring the need for concurrent obesity prevention and treatment of associated diseases. For example, the median county-level obesity prevalence for the AIAN population in counties with tribal reservations was only slightly higher than in counties without reservations, while the diabetes YLL rates attributed to non-optimal BMI were nearly twice as high. AIAN individuals face an elevated risk of premature mortality because they bear the legacy and ongoing harms of colonization, contending with lower socioeconomic status and more limited access to health care than other racial and/or ethnic populations [91, 92]. These issues are particularly acute on reservations, where health care and nutritional assistance are inadequate, inaccessible, and underfunded, despite the US government’s treaty obligations to provide these services [91–94].

Rising obesity prevalence is a major public health concern that traditional public health approaches have not yet substantially abated in the USA or in other countries [4]. One promising pharmacological intervention is glucagon-like peptide-1 receptor agonists (GLP-1s), a class of drugs originally developed to treat type 2 diabetes and recently approved to treat obesity in the USA. Participants with overweight or obesity who took GLP-1s were much more likely to experience clinically meaningful weight reduction compared with those using lifestyle modification alone after at least one year [95]. This class of drugs also reduced the incidence of comorbidities such as type 2 diabetes [96] and cardiovascular events [97, 98]. A small percentage of eligible adults take GLP-1s for weight loss [99], and the impact of these drugs on population health ultimately will depend on their long-term effectiveness, tolerability (some trials noted high attrition due to side effects), and accessibility [95, 100, 101]. Uneven access to GLP-1s for weight loss [99] and diabetes treatment [102] could worsen existing disparities in obesity prevalence and disease severity. The price of GLP-1s and the number of people who would need to take these drugs over decades to sustain population reductions in obesity make health system costs of GLP-1s a major concern [103].

We used BMI ≥ 30 to define obesity because it is the most widely studied measure of obesity and has been consistently linked to a wide range of adverse health outcomes [1, 104]. Furthermore, BMI data are available at granular demographic and geographic levels and over time in the USA, validation studies have found a strong correlation between BMI and direct fat measurements [105], and nearly all adults with BMI ≥ 30 (or BMI ≥ 27.5 for the Asian population) also have excess adiposity [106]. However, the use of BMI to identify people with obesity has been debated because BMI is not a direct measure of adiposity, and BMI does not define a precise health state for individuals [104]. Heterogeneity in the association between BMI and disease outcomes among specific racial and/or ethnic populations raises concerns for certain groups. In particular, Asian populations have a higher relative risk of obesity-related comorbidities at a given BMI [107–109].

This study has several limitations. First, we adjusted BMI data for self-report bias at the national level, using measured BMI data from NHANES [36]. We relied on the more detailed geographic resolution of self-reported BMI data from BRFSS and Gallup to inform our estimates at the county level. This approach assumes that self-report bias does not vary by location, but we could not validate this at the state or county levels. An additional limitation is that NHANES did not report data specifically for the Asian population until 2012 and grouped the AIAN and Multiracial populations into a residual “Other” category in all years. Consequently, we could not obtain unique crosswalk parameter estimates for these races and/or ethnicities and instead applied crosswalk adjustments estimated from data pooled across these specific populations. Second, like other non-response bias adjustment techniques, multilevel regression and post-stratification assumes that respondents and non-respondents are alike within a given stratum. Third, BRFSS sometimes reported coarser geographic and age detail than we targeted. Our approach accounted for nesting of detailed strata within coarser strata but could not recover the full variation within aggregated strata [26]. Fourth, our approach assumes the same relative risk of mortality at a given level of BMI for all racial and/or ethnic populations, locations, and years. The association between BMI and diabetes has been shown to vary by race and/or ethnicity [108, 110], but there is insufficient evidence to robustly estimate race and/or ethnicity-specific relative risk curves for each health outcome. Fifth, we aggregated Asian and NHPI populations due to data constraints. This aggregation masks disparities between the Asian and NHPI populations [111], and the relative sizes of these populations may influence geographical disparities in the combined Asian population. The Hawaiian counties were the only locations where the combined Asian population had a higher obesity prevalence than the White population in 2019, while NHPI people made up a much greater proportion of this population than in other counties [24]. Sixth, our study covers only the pre-COVID period. In the first year of the pandemic, obesity prevalence increased by 1.1 percentage points [112], and obesity elevated the risk of hospitalization or death related to COVID-19 [113, 114], thereby magnifying disparities in virus exposure [115]. While we did not present estimates related to COVID-19, our conclusion that disparities in obesity are compounded by disparities in mortality likely extends to COVID-19 pandemic years and beyond. Finally, our estimates of morbidity and mortality attributable to non-optimal BMI have considerable uncertainty, reflecting heterogeneity in the literature in associations between non-optimal BMI and mortality [1].

## Conclusions

Disparities between racial and/or ethnic populations in YLLs attributable to non-optimal BMI greatly exceeded disparities in obesity alone. Prevention and treatment advances for cardiovascular disease, colorectal cancer, and diabetes have substantially reduced these forms of mortality, despite increasing population levels of obesity. In addition to reducing disparities in obesity, addressing differences in prevention and treatment of diseases associated with obesity is necessary to eliminate disparities in obesity-related mortality. These findings can inform multilevel policies and prevention strategies aimed at reducing obesity prevalence and alleviating the impact of specific diseases associated with non-optimal BMI in the USA.

## Supplementary Information

Additional file 1: Section 1 GATHER table; Section 2 Further details on data and processing; Supplementary tables—Table S1 Cause hierarchy for attributable burden; Table S2 Counties combined to create historically stable units of analysis; Table S3 Covariate data sources; Table S4 Population data sources; Table S5 Sources of survey data; Table S6 Summary of measurement types, years, and resolution of BMI data; Table S7 Population mask; Table S8 Ensemble distribution weights. Supplementary figures—Figure S1 Data and modeling flowchart; Figure S2 Age-standardized prevalence of obesity, 2019, male; Figure S3 Age-standardized prevalence of obesity, 2019, female; Figure S4 Age-standardized attributable YLL rates and PAFs, all causes, 2000–2019; Figures S5–S42 Age-standardized attributable YLL rates and PAFs for various health conditions, 2000–2019.

## Data Availability

Estimates of annual prevalence of obesity and YLLs attributable to non-optimal BMI, by county, age, sex, and race and/or ethnicity are available for download from the Global Health Data Exchange (https://ghdx.healthdata.org/us-non-optimal-bmi-prevalence-ylls-2000-2019). Information about the underlying data sources is available in the supplementary materials (pp 34–46). The code used for this analysis is available on GitHub: https://github.com/ihmeuw/USHD.

## Abbreviations

AIAN: American Indian and Alaska Native
BMI: Body mass index
BRFSS: Behavioral Risk Factor Surveillance System
CoV: Coefficient of variation
GBD: Global Burden of Diseases, Injuries, and Risk Factors Study
GLP-1s: Glucagon-like peptide-1 receptor agonists
IHD: Ischemic heart disease
IQR: Interquartile range
NHANES: National Health and Nutrition Examination Survey
NHPI: Native Hawaiian and Pacific Islander
PAF: Population attributable fraction
SAE: Small-area estimation
TMREL: Theoretical minimum risk exposure level
UI: Uncertainty interval
YLL: Years of life lost
