# Supplementary material for: Cause-specific years of life lost attributable to non-optimal body mass index by county, sex, race, and ethnicity in the USA, 2000–2019: a systematic analysis of health disparities
Source: BMC Med. 2026 Mar 18;24:268. doi: 10.1186/s12916-026-04795-y (PMC13112685; doi:10.1186/s12916-026-04795-y)
Supplement: Supplementary file 1 — Additional file 1: Section 1 GATHER table; Section 2 Further details on data and processing; Supplementary tables—Table S1 Cause hierarchy for attributable burden; Table S2 Counties combined to create historically stable units of analysis; Table S3 Covariate data sources; Table S4 Population data sources; Table S5 Sources of survey data; Table S6 Summary of measurement types, years, and resolution of BMI data; Table S7 Population mask; Table S8 Ensemble distribution weights. Supplementary figures—Figure S1 Data and modeling flowchart; Figure S2 Age-standardized prevalence of obesity, 2019, male; Figure S3 Age-standardized prevalence of obesity, 2019, female; Figure S4 Age-standardized attributable YLL rates and PAFs, all causes, 2000–2019; Figures S5–S42 Age-standardized attributable YLL rates and PAFs for various health conditions, 2000–2019. [file 12916_2026_4795_MOESM1_ESM.pdf]

Additional file 1: Cause-specific years of life lost (YLLs) attributable to non-optimal body-mass index by county, sex, race, and ethnicity in the USA, 2000–19: a systematic analysis of health disparities

Contents

Contents 1

1 GATHER Checklist 5

2 Supplementary Methods 7

2.1 Data sources and processing 7

2.1.1 Population data 7

2.1.2 Prevalence data 8

2.1.3 Mortality data 8

2.2 Spatial units 9

2.3 Self-report bias correction 9

2.3.1 Rationale 10

2.3.2 Method details 10

2.3.3 Exclusions 14

2.4 Estimation of overweight and obesity prevalence 14

2.4.1 Outcome definition 14

2.4.2 Overweight and obesity model specification 15

2.4.3 Addressing data misalignment 18

2.4.4 Post-stratification frame 20

2.5 Covariates 21

2.6 Population distribution of BMI 24

2.6.1 Mean BMI 24

2.6.2 Ensemble weights 25

2.6.3 Standard deviation optimisation 26

2.7 Calculation of Attributable Burden 26

2.7.1 Relative risk curves and theoretical minimum risk exposure level 27

2.7.2 Population attributable fraction 27

2.7.3 Attributable burden 27

Supplementary Tables 29

Table S1. Cause hierarchy for attributable burden 29

Table S2. Counties combined to create historically stable units of analysis 31

Table S3. Covariate data sources 32

Table S4. Population data sources 36

Table S5. Sources of survey data 38

Table S6. Summary of measurement types, years, and resolution of BMI data 45

Table S7. Population mask 47

Table S8. Ensemble distribution weights 48

Supplementary Figures 49

Figure S1. Data and modelling flowchart 49

Figure S2. Age-standardised prevalence of obesity (BMI ≥ 30), 2019, male 50

Figure S3. Age-standardised prevalence of obesity (BMI ≥ 30), 2019, female 51

Figure S4. Age-standardised attributable YLL rates and PAFs, all causes, 2000–2019 52

Figure S5. Age-standardised attributable YLL rates and PAFs, non-communicable, 2000–2019 53

Figure S6. Age-standardised attributable YLL rates and PAFs, neoplasms, 2000–2019 54

Figure S7. Age-standardised attributable YLL rates and PAFs, colorectal cancer, 2000–2019 55

Figure S8. Age-standardised attributable YLL rates and PAFs, liver cancer, 2000–2019 56

Figure S9. Age-standardised attributable YLL rates and PAFs, gallbladder cancer, 2000–2019 57

Figure S10. Age-standardised attributable YLL rates and PAFs, pancreatic cancer, 2000–2019 58

Figure S11. Age-standardised attributable YLL rates and PAFs, breast cancer, 2000–2019 59

Figure S12. Age-standardised attributable YLL rates and PAFs, uterine cancer, 2000–2019 60

Figure S13. Age-standardised attributable YLL rates and PAFs, ovarian cancer, 2000–2019 61
Figure S14. Age-standardised attributable YLL rates and PAFs, kidney cancer, 2000–2019 62
Figure S15. Age-standardised attributable YLL rates and PAFs, thyroid cancer, 2000–2019 63
Figure S16. Age-standardised attributable YLL rates and PAFs, lymphoma, 2000–2019 64
Figure S17. Age-standardised attributable YLL rates and PAFs, Burkitt lymphoma, 2000–2019 65
Figure S18. Age-standardised attributable YLL rates and PAFs, other lymphoma, 2000–2019 66
Figure S19. Age-standardised attributable YLL rates and PAFs, myeloma, 2000–2019 67
Figure S20. Age-standardised attributable YLL rates and PAFs, leukemia, 2000–2019 68
Figure S21. Age-standardised attributable YLL rates and PAFs, acute lymphoid leukemia, 2000–2019 . 69
Figure S22. Age-standardised attributable YLL rates and PAFs, chronic lymphoid leukemia, 2000–2019 70
Figure S23. Age-standardised attributable YLL rates and PAFs, acute myeloid leukemia, 2000–2019 71
Figure S24. Age-standardised attributable YLL rates and PAFs, chronic myeloid leukemia, 2000–2019. 72
Figure S25. Age-standardised attributable YLL rates and PAFs, other leukemia, 2000–2019 73
Figure S26. Age-standardised attributable YLL rates and PAFs, cardiovascular diseases, 2000–2019 74
Figure S27. Age-standardised attributable YLL rates and PAFs, ischemic heart disease, 2000–2019 75
Figure S28. Age-standardised attributable YLL rates and PAFs, stroke, 2000–2019 76
Figure S29. Age-standardised attributable YLL rates and PAFs, ischemic stroke, 2000–2019 77
Figure S30. Age-standardised attributable YLL rates and PAFs, intracerebral hem, 2000–2019 78
Figure S31. Age-standardised attributable YLL rates and PAFs, subarachnoid hem, 2000–2019 79
Figure S32. Age-standardised attributable YLL rates and PAFs, hypertensive heart disease, 2000–2019 80
Figure S33. Age-standardised attributable YLL rates and PAFs, atrial fibrillation, 2000–2019 81
Figure S34. Age-standardised attributable YLL rates and PAFs, chronic respiratory, 2000–2019 82
Figure S35. Age-standardised attributable YLL rates and PAFs, asthma, 2000–2019 83
Figure S36. Age-standardised attributable YLL rates and PAFs, digestive diseases, 2000–2019 84

Figure S37. Age-standardised attributable YLL rates and PAFs, gallbladder & biliary, 2000–2019..........85

Figure S38. Age-standardised attributable YLL rates and PAFs, neurological disorders, 2000–2019 86

Figure S39. Age-standardised attributable YLL rates and PAFs, Alzheimer's disease, 2000–2019...........87

Figure S40. Age-standardised attributable YLL rates and PAFs, diabetes & CKD, 2000–2019 88

Figure S41. Age-standardised attributable YLL rates and PAFs, diabetes, 2000–2019............................89

Figure S42. Age-standardised attributable YLL rates and PAFs, diabetes type 2, 2000–2019 90

1 GATHER Checklist

Item #	Checklist item	Description of Compliance
Objectives and funding		
1	Define the indicator(s), populations (including age, sex, and geographic entities), and time period(s) for which estimates were made.	Methods section
2	List the funding sources for the work.	Summary, acknowledgments
Data Inputs		
For all data inputs from multiple sources that are synthesized as part of the study:		
3	Describe how the data were identified and how the data were accessed.	Methods section, acknowledgements, Additional file 1: section 2 and Tables S3–S5
4	Specify the inclusion and exclusion criteria. Identify all ad-hoc exclusions.	Methods section, Additional file 1: section 2 and Table S6
5	Provide information on all included data sources and their main characteristics. For each data source used, report reference information or contact name/institution, population represented, data collection method, year(s) of data collection, sex and age range, diagnostic criteria or measurement method, and sample size, as relevant.	Methods section, acknowledgements, Additional file 1: section 2 and Tables S3–S5
6	Identify and describe any categories of input data that have potentially important biases (eg, based on characteristics listed in item 5).	Methods section, discussion, Additional file 1: section 2
For data inputs that contribute to the analysis but were not synthesized as part of the study:		
7	Describe and give sources for any other data inputs.	Methods section, Additional file 1: section 2.
For all data inputs:		
8	Provide all data inputs in a file format from which data can be efficiently extracted (eg, a spreadsheet rather than a PDF), including	GHDx link

	all relevant meta-data listed in item 5. For any data inputs that cannot be shared because of ethical or legal reasons, such as third-party ownership, provide a contact name or the name of the institution that retains the right to the data.	
Data analysis		
9	Provide a conceptual overview of the data analysis method. A diagram may be helpful.	Methods section, Additional file 1: section 2 and Figure S1
10	Provide a detailed description of all steps of the analysis, including mathematical formulae. This description should cover, as relevant, data cleaning, data pre-processing, data adjustments and weighting of data sources, and mathematical or statistical model(s).	Methods section, Additional file 1: section 2
11	Describe how candidate models were evaluated and how the final model(s) were selected.	Additional file 1: section 2
12	Provide the results of an evaluation of model performance, if done, as well as the results of any relevant sensitivity analysis.	N/A
13	Describe methods for calculating uncertainty of the estimates. State which sources of uncertainty were, and were not, accounted for in the uncertainty analysis.	Methods section, Additional file 1: section 2
14	State how analytic or statistical source code used to generate estimates can be accessed.	GitHub
Results and Discussion		
15	Provide published estimates in a file format from which data can be efficiently extracted.	GHDx link
16	Report a quantitative measure of the uncertainty of the estimates (e.g. uncertainty intervals).	Results section; GHDx link
17	Interpret results in light of existing evidence. If updating a previous set of estimates, describe the reasons for changes in estimates.	Discussion section
18	Discuss limitations of the estimates. Include a discussion of any modelling assumptions or data limitations that affect interpretation of the estimates.	Discussion section, Additional file 1: section 2

2 Supplementary Methods

The methods used for this analysis are an extension of methods for estimating cause-specific mortality[19] and healthy life expectancy[26] by US county and racial and/or ethnic population and for calculating risk-attributable burden.[1]

2.1 Data sources and processing

2.1.1 Population data

We processed population data from various sources (Table S4) to derive three sets of population estimates by county, age group (20–24, …, 80–84, 85+ years of age), sex (male or female), year (2000–19), and either (1) racial and/or ethnic population, (2) educational attainment (four categories: Less than High School, High School, Some College, and BA Degree or Higher), or (3) marital status (three categories: Currently Married, Formerly Married, and Never Married). Population by racial and/or ethnic population was derived from data from the National Center for Health Statistics (NCHS). Population by educational attainment and population by marital status were derived from county-level population data from the US Census and American Community Survey (ACS), stratified separately by age, sex, and educational attainment or marital status, plus state-level population distributions by marital status, educational attainment, age, sex, and year, from Public Use Microdata Series (PUMS) files. The creation of the joint population file is described in section 2.4.4.

We classified racial and/or ethnic populations in accordance with the standards for federal data collection on race and/or ethnicity issued in 1977 by the Office of Management and Budget (OMB).[21] Although more detailed race categories are available for most years in the Behavioral Risk Factor Surveillance System (BRFSS) and Gallup, we used the 1977 OMB standards for all analyses so that we could calculate attributable burden, which uses Years of Life Lost (YLLs) as a primary input. There are several significant challenges to estimating mortality (and thus YLLs) using the 1997 OMB standards. Although these standards were updated in 1997 to require that federal data collection create distinct Asian and Native Hawaiian or Pacific Islander (NHPI) populations and the option to identify as multiple races, those changes were not fully realised on death certificates until 2018, and data on misclassification of racial and/or ethnic population on death certificates are only available using the 1977 OMB standards. It is also not possible to disaggregate Asian and NHPI populations on death certificates prior to 2011 due to the use of a combined Other Asian and Pacific Islander residual category. We therefore categorised racial and/or ethnic populations as American Indian or Alaska Native (AIAN), Asian or Pacific Islander (Asian), Black,

Latino or Hispanic of any race (Latino), and White. The mortality analysis used the imputed or "bridged" race estimates from the National Center for Health Statistics, which were derived from models[27] to predict "primary race" for individuals who report multiple races, thereby bridging Census responses made using the 1997 race categories to the simpler 1977 categories that we used for our analysis.

2.1.2 Prevalence data

We extracted microdata (ie, unaggregated individual-level data) from the National Health and Nutrition Examination Survey (NHANES),[18] BRFSS,[16] and Gallup Daily.[17] These surveys employ complex designs to produce samples that are representative of the non-institutionalised US population across all ages (NHANES) or only adults (ages 18+, BRFSS and Gallup).[28-30] Gallup provided full geographical coverage for the USA from 2008 to 2017 at the county level, but data resolution for BRFSS was more variable, with county-level identifiers not uniformly provided in all states and years (Table S6). NHANES data were only available at the national level. We used the versions of racial and/or ethnic population variables provided by the survey series that conformed to the 1977 OMB guidelines, when possible, but otherwise combined separately reported Asian and NHPI populations. Gallup reported all race and/or ethnicity populations selected by individuals identifying as multiracial; we imputed a "primary race" for each of these individuals using bridging models developed by Liebler.[25] We restricted the sample to respondents 20 years of age or older with complete responses related to body-mass index (BMI) and sociodemographic variables used in this analysis. We excluded respondents who reported being currently pregnant in BRFSS and NHANES, but not in Gallup due to its lack of pregnancy status variable.

Each survey included self-reported height and weight, which are used to calculate self-reported BMI. NHANES also included objectively measured height and weight. We collapsed the survey microdata to produce stratum-level estimates of overweight and obesity (BMI ≥ 25) and obesity alone (BMI ≥ 30). For each outcome and data source, we derived counts and sample sizes stratified by county, year, age, sex, racial and/or ethnic population, educational attainment, and marital status. We produced survey-weighted counts for each stratum to account for unequal sampling probability and nonresponse bias. We did not adjust uncertainty within these stratification categories because sample sizes were insufficient to consistently derive design effects at this high level of stratification.

2.1.3 Mortality data

We obtained YLL estimates by county, race and/or ethnicity, age, sex, year, and cause of death, which were produced in previous work.[1] These estimates were used to calculate cause-specific YLLs attributable to non-optimal BMI and the population attributable fraction (sections 2.7.2, 2.7.3, Table S1).

2.2 Spatial units

Our analysis focused on county-level estimates of non-optimal BMI and attributable burden. However, a small number of counties underwent boundary changes during our modeling time frame. We therefore combined a small number of counties into merged geographic entities with stable boundaries over time; we refer to these 3,110 counties or merged-county units as "counties," collectively encompassing all 3,143 counties or county equivalents existing in 2019 (the merged units are detailed in Table S2).

BRFSS sometimes reported microdata at a spatial resolution that is coarser than county level. BRFSS microdata were obtained through a combination of public or limited use files, Selected Metropolitan/Micropolitan Area Risk Trends (SMART) BRFSS, and state-provided files (Table S5). These sources variously provided identifiers for counties, Core-Based Statistical Areas (CBSAs, including Metropolitan and Micropolitan Statistical Areas), metropolitan divisions, or states as the smallest geographic areas reported, depending on the source, state, and year. We used the source that gave the highest geographical resolution for a given location and year. We developed aggregated geographic units for each model year to harmonise the spatial resolution of the available BRFSS data with the county-level spatial units that we were modelling. BRFSS microdata were then collapsed by the resultant "CBSA-county" units. In some state-years, this process resulted in greater spatial detail in urban areas, with the bulk of rural areas falling into "state remainder" spatial units. We designed our modelling framework cognisant of this varied spatial resolution, using a regression approach (section 2.4.3) that retains as much information as possible about spatial variation in overweight and obesity.

2.3 Self-report bias correction

BMI calculated from objectively measured height and weight ("measured BMI") is the gold standard for the population distribution of BMI, but sources with measured BMI had limited sample sizes and geographic resolutions. We developed a model to correct self-reported BMI from BRFSS and Gallup for self-report bias. Our approach accounted for differences in self-report bias across the BMI distribution, interview mode, and key sociodemographic characteristics. NHANES, BRFSS, and Gallup had the same target population. We assumed that differences between measured BMI in NHANES and self-reported BMI in Gallup and BRFSS resulted from self-report bias. The adjustment was based on respondents' positions in the BMI distributions, rather than their self-reported BMI, because self-report bias tends to be larger in magnitude in telephone surveys than in face-to-face interviews.[31] Using measured BMI from NHANES to adjust for self-report bias in BRFSS and Gallup allowed us to leverage the larger sample sizes and better geographic resolution of these surveys.

The BMI self-report correction model had three main steps. First, we calculated observed self-report bias by comparing measured BMI in NHANES to self-reported BMI in BRFSS and Gallup for comparable populations. Then, we fitted a model estimating the self-report bias (log-ratio of measured to self-reported BMI). Finally, we estimated the self-report bias for all rows in the BRFSS and Gallup microdata and predicted measured BMI.

2.3.1 Rationale

We adjusted for systematic biases of self-reported BMI to accurately characterise the population distribution of BMI. Self-reported BMI is generally lower and has a narrower distribution compared to measured BMI.[32-35] Individuals in the overweight and obese categories (BMI ≥ 25) tend to underreport their BMI, while those in the underweight category (BMI < 18.5) tend to overreport.[32,34] This bias varies by sex, age, racial and/or ethnic population, and interview mode (eg, face-to-face or telephone).[31,34-36] While biases in self-reported BMI are well-documented, most bias correction techniques do not capture the complex patterns of self-report bias.[34-39]

Our model used random effects on age group and racial and/or ethnic population, fitted separately by sex, to account for differences in self-report bias across demographic groups. Additionally, the self-report correction varied by self-reported BMI quantile. We assumed that the relative ordering of respondents' self-reported BMI was consistent across interview modes (adjusted for demographic groups), without assuming a similar magnitude of bias in NHANES, BRFSS, and Gallup.

There was considerable uncertainty in the self-report correction. Differences between a respondent's self-reported and measured BMI vary stochastically and systematically, meaning that individuals with the same self-reported BMI have a wide range of actual BMIs.[36,37,40] We generated ten predictions of measured BMI for each individual to propagate uncertainty from the self-report correction to subsequent steps.

2.3.2 Method details

Calculating observed self-report bias

We defined self-report bias as the log-ratio of measured to self-reported BMI, using BRFSS and Gallup for self-reported values and NHANES for measured values. We estimated self-report bias between surveys rather than estimating self-report bias within NHANES due to differences in the interview modes, which affect self-report bias.[31]

We stratified the microdata by source, sex, race and/or ethnicity, age group, period, and quantile of self-reported BMI. Within each stratum, we calculated the mean and standard error of BMI using survey weights. We assumed the weighted sample mean ($\overline{X_i}$) was normally distributed with mean μ_i and standard deviation $\frac{\sigma_i}{\sqrt{n_i}}$, the weighted standard deviation of the data divided by the square root of the sample size.

Next, we paired NHANES measured BMI data with BRFSS and Gallup self-reported BMI data for comparable populations. We calculated the log-ratio of measured to self-reported mean BMI using the delta method and assuming independence between the means. The log-sample mean had the following distribution:

$$\log(\overline{X_i}) \sim \text{Normal}\left(\log(\mu_i), \frac{\sigma_i}{\mu_i\sqrt{n_i}}\right) \quad (1)$$

The log-ratio of the paired means was:

$$\log(R) = \log\left(\frac{\overline{X_2}}{\overline{X_1}}\right) \sim \text{Normal}\left((\log(\mu_2) - \log(\mu_1), \sqrt{\left(\frac{\sigma_2}{\mu_2\sqrt{n_2}}\right)^2 + \left(\frac{\sigma_1}{\mu_1\sqrt{n_1}}\right)^2}\right) \quad (2)$$

where indices 1 and 2 refer to self-reported and measured BMI, respectively. We selected stratification variables to account for variations in self-report bias across populations while maintaining sufficient sample sizes in each stratum to reliably calculate mean BMI. These stratifications differ from the main analysis due to the less detailed racial and/or ethnic populations in NHANES and our interest in maintaining sufficient sample sizes in each stratum.

We collapsed the microdata by age group, sex, and four racial and/or ethnic populations that correspond to those reported by NHANES for the entire study period: Latino, Black only, White only, and combined remaining populations (Asian, Native Hawaiian or Pacific Islander [NHPI], AIAN, Multiracial, and self-reported "other"). We also collapsed the data by period. Each two-year period of BRFSS and Gallup data was matched to three waves of NHANES data. For example, the mean BMI calculated from the 2009 and 2010 BRFSS datasets was compared to the mean BMI for the equivalent population in NHANES calculated across the 2007–2008, 2009–2010, and 2011–2012 waves. Following NHANES analytical guidelines,[28,41] we pooled waves of NHANES data to increase the stability of estimates. We included BRFSS data from 1999 and 2020 to align with NHANES years, even though we did not produce estimates for 1999 and 2020. We created 15 groups of self-reported BMI by quantile because self-report bias varied by BMI level.

Using 15 groups provided flexibility in self-report correction across the BMI distribution, while maintaining sufficient sample sizes in most stratum to calculate its mean BMI. Empirical survey-weighted quantiles of self-reported BMI were calculated separately by source, 20-year age group, sex, and period.

Modeling self-report bias

We fitted the following model of self-report bias in R-INLA[42] separately by sex:

$$\log(R_{a,r,p,t,d}) \sim \text{Normal}(\mu_{a,r,p,t,d}, s_{a,r,p,t,d} \cdot \sigma_\mu) \quad (3)$$

$$\mu_{a,r,p,t,d} = \beta_0 + \gamma_{1,a} + \gamma_{2,r} + \gamma_{3,a,r} + \gamma_{4,p,a} + \gamma_{5,d} + \gamma_{6,d,r} \quad (4)$$

The log-ratio of measured to self-reported mean BMI ($\log(R_{a,r,p,t,d})$) had a normal distribution with mean $\mu_{a,r,p,t,d}$ and standard deviation σ_μ, scaled by $s_{a,r,p,t,d}$, the standard error of $\log(R_{a,r,p,t,d})$. The log-ratio was calculated by age group (a), race and/or ethnicity population (r), BMI percentile bin (p), period (t), and data source (d).

The model included the following terms:

- β_0: global intercept
- $\gamma_{1,a}$: random intercept for age groups (10-year bins from 20–29 to 70–79, and 80+) with a first-order random walk prior
- $\gamma_{2.r}$: random intercept on racial and/or ethnic population
- $\gamma_{3,a,r}$: random intercept on racial and/or ethnic population and age group combinations
- $\gamma_{4,p,a}$: random intercept on the self-reported BMI percentile bin with a second-order random walk, replicated by age group
- $\gamma_{5,d}$: random intercept on data source
- $\gamma_{6,d,r}$: random intercept on combinations of data source and racial and/or ethnic population

The following priors were used:

- $\beta_0 \sim \text{Normal}(\text{mean} = 0.0, \text{precision} = 0.0)$
- $\gamma_{1,a} \sim RW1(\sigma_1)$
- $\gamma_{2.r} \sim IID(\sigma_2)$
- $\gamma_{3,a,r} \sim IID(\sigma_3)$
- $\gamma_{4,p,a} \sim RW2{:}\,IID(\sigma_4)$
- $\gamma_{5,d} \sim IID(\sigma_5)$
- $\gamma_{6,r,d} \sim IID(\sigma_6)$

The precision parameters had the following hyperpriors:

- $\sigma_1^{-2}, \sigma_2^{-2}, \sigma_3^{-2}, \sigma_5^{-2}, \sigma_6^{-2} \sim PC(\sigma_0 = 5, \alpha = 0.05) \rightarrow Pr(\sigma > 5) = 0.05$
- $\sigma_4^{-2} \sim PC(\sigma_0 = 0.5, \alpha = 0.05) \rightarrow Pr(\sigma > 0.5) = 0.05$
- $\sigma_\mu^{-2} \sim \text{Gamma}(\text{shape} = 1, \text{inverse-scale} = 0.01)$

We used weakly informative penalized complexity (PC) hyperpriors for the precision of each random effect to favour smaller random effects (ie, less variation between populations) in the absence of contrary evidence.[43] The precision of the Gaussian response had a Gamma hyperprior. To improve numerical stability, we scaled the response variable by 100 before estimation and adjusted the precision accordingly. The response was transformed back into log-ratio space during the prediction step.

We included source effects to account for consistent differences in coverage bias between data sources. In 2011, BRFSS introduced a combined landline and cell phone sampling frame, which abruptly changed coverage bias compared to the landline-only sampling frame.[44] We treated the BRFSS combined landline and cell phone sample and the BRFSS landline-only sample as different sources. We did not include year parameters in the model to ensure that self-reported trends from BRFSS and Gallup were maintained rather than shifted to match NHANES exactly. Differences between BRFSS and Gallup relative to NHANES changed over time, which is not entirely due to changes in self-report bias. For example, differences between self-reported BRFSS and measured NHANES estimates of overweight and obesity prevalence increased between 1999 and 2020 for Latino males. This could indicate an increase in self-report bias for this population. However, the ratio of self-reported and measured overweight and obesity prevalence within NHANES remained fairly stable over time, suggesting only modest changes in self-report bias. A more likely explanation for the increasing divergence between BRFSS and NHANES for Latino males is changes in the NHANES survey design, which oversampled Mexican-Americans from 1999 to 2006 and oversampled the entire Latino population starting in 2007.[45] By excluding time parameters in our self-report correction model, we conservatively assumed that self-report bias did not change (on a multiplicative scale) over time. This assumption allowed our estimates to reflect time trends in self-reported BMI in BRFSS and Gallup, which have much larger sample sizes than NHANES.

Predicting measured BMI

Ten samples from the approximate posterior distribution of the log-ratio of measured to self-reported BMI were taken for each respondent in BRFSS and Gallup. The estimated log-ratio of self-report bias was used to predict the self-report adjusted BMI for all respondents. Each sample represented one imputation

of the dataset. We ran subsequent models separately on the ten imputed datasets to propagate uncertainty from the self-report correction into the estimates of overweight and obesity prevalence.

2.3.3 Exclusions

We excluded strata where the assumption that NHANES represented the same population as BRFSS and Gallup was violated. This was primarily due to methodological changes in the surveys. Most discrepancies concerned the broad "Other" category in NHANES, which included the Asian, NHPI, AIAN, Multiracial, and other populations. This category had small sample sizes and was composed of populations with heterogeneous BMI distributions, making the estimates sensitive to changes in NHANES survey design or weighting.

We excluded all data for Asian, NHPI, AIAN, Multiracial, and other populations in Gallup. These populations were inconsistently defined over time in the survey series, and the relative survey weights of each population did not align with the relative weights of these populations in BRFSS, nor with the relative weight of the Asian population in NHANES, in the waves where that population was reported.

We excluded certain strata from BRFSS for fitting the self-report adjustment model, although we did not apply these exclusions to the inputs of the small-area estimation models because concerns about the survey representativeness were focused on NHANES. We excluded the Asian, NHPI, AIAN, Multiracial, and other populations from 1999 to 2002 because of large changes in overweight and obesity prevalence corresponding to a change in the NHANES target population over this period.[45] We also excluded this population from 2017 to 2020 because differences between BRFSS and NHANES prevalence estimates for these populations were significantly larger than in previous waves, which likely resulted from COVID-19-related disruptions to the NHANES survey design.[28] We also excluded Asian, NHPI, AIAN, Multiracial, and other populations of adults over the age of 60 from 2003 to 2010 because the estimates were unstable over time and the sample sizes were very small. Lastly, strata with fewer than five respondents were excluded because the mean BMI could not be precisely calculated.

2.4 Estimation of overweight and obesity prevalence

2.4.1 Outcome definition

Prevalence of overweight and obesity and of obesity alone are defined as having a BMI greater than or equal to 25 and 30, respectively. To ensure that prevalence of obesity is consistently lower than

prevalence of overweight and obesity combined, we modelled obesity conditional on overweight and obesity prevalence. We multiplied the obesity proportion with the prevalence of overweight and obesity to obtain the prevalence of obesity:

$$P(X \geq 30) = P(X \geq 30 | X \geq 25) \cdot P(X \geq 25) \tag{5}$$

2.4.2 Overweight and obesity model specification

We used small-area estimation models to estimate prevalence of overweight and obesity and of obesity alone. Models were fitted separately by sex on ten imputations of self-report adjusted overweight and obesity data (sections 2.1.2 and 2.3). We created predictions by county, race and/or ethnicity, sex, age group, year, source, educational attainment, and marital status. One hundred draws per imputation were generated from the approximated posterior distribution, totalling 1,000 draws per stratum. Draws were then collapsed across education and marital status variables by calculating the weighted-mean prevalence of overweight and obesity by age, sex, race and/or ethnicity, and year at the county-level to improve population representativeness (section 2.4.4). We fitted these models with TMB because TMB is very flexible with respect to model specifications,[46] for example by enabling us to incorporate higher-order interaction terms and assimilate input data with different granularity (section 2.4.3).

$$Y_{j,t,a,r,e,m,d} \sim \text{Binomial}\left(p_{j,t,a,r,e,m,d}, n_{j,t,a,r,e,m,d}\right) \tag{6}$$

$$\text{logit}\left(p_{j,t,a,r,e,m,d}\right) = \beta_0 + \boldsymbol{\beta}_1 \cdot \boldsymbol{X}_{1,j,t,r} + \sum_{t'=1}^{k_t} \sum_{a'=1}^{k_a} \left(\gamma_{1,j,t',a',r} \cdot S_{t'}(t) \cdot S_{a'}(a)\right) \tag{7}$$

$$+ \gamma_{2,j} + \sum_{t''=1}^{k_t} \boldsymbol{\beta}_{2,t''} \cdot S_{t''}(t) + \sum_{a''=1}^{k_a} \boldsymbol{\beta}_{3,a''} \cdot S_a(a) + \gamma_{3,a,r,e,m} + \gamma_{4,r,d|d \neq BRFSS\_LLCP}$$

$$+ \gamma_{5,a,d|d \neq BRFSS\_LLCP}$$

Priors:

- $\gamma_1 \sim$ LCAR: LCAR: LCAR: IID$(\rho_{1,j}, \rho_{1,t'}, \rho_{1,a'}, \sigma_1)$
- $\gamma_2 \sim$ LCAR$\left(\rho_{2,\mathrm{j}}, \sigma_2\right)$
- $\gamma_3 \sim$ LCAR: IID: LCAR: IID$\left(\rho_{3,a}, \rho_{3,e}, \sigma_3\right)$
- $\gamma_4 \sim IID(\sigma_4)$
- $\gamma_5 \sim LCAR: IID(\rho_{5,a}, \sigma_5)$

Hyperpriors:

- $\sigma^{-2} \sim \text{PC}(\sigma_0 = 5, \alpha = 0.05) \rightarrow \Pr(\sigma > 5) = 0.05$

- $\text{logit}(\rho) \sim \text{Normal}(0, \text{variance} = 1.5^2)$

where $Y_{j,t,\text{a},r,e,m,d}$ is the cases of BMI ≥ 25 or BMI ≥ 30 in county j, year t, age group a, racial and/or ethnic population r, educational attainment e, marital status m, and data source d, with prevalence p. n is the sample size of the stratum in the overweight and obesity model, and n is the number of respondents with BMI ≥ 25 in the stratum in the obesity proportion model. We treated the combined landline and cell phone sample of BRFSS as the gold-standard source because it has more consistent definitions of race and/or ethnicity populations than Gallup and less coverage bias than the landline-only sample of BRFSS used prior to 2011.[44] Estimating source effects also improved our identification of the time trend, as each source had different temporal coverage.

- β_0: global intercept
- $\mathbf{X}_{1,j,t,r}$: vector of covariates (poverty rate, percentage born outside the US, population density) for county j, year t, and racial and/or ethnic population r (for poverty rate and percentage born outside the US)
- $\boldsymbol{\beta}_1$: the associated vector of fixed effect regression coefficients
- $\gamma_{1,j,t',a',r}$: random intercept for county, racial and/or ethnic population, year spline basis, and age spline basis combinations
- k_t: the number of time knots (five knots, evenly spaced from 2000 to 2019)
- t': the corresponding index
- k_a: the number of age knots (four knots, placed at age groups 20–24, 40–44, 65–69, and 85+
- a': the corresponding index
- $S_{t'}(t)$: the value of linear spline basis t' on year, evaluated at year t
- $S_{a'}(a)$: the value of linear spline basis a' on age, evaluated at age group a
- $\gamma_{2,j}$: random intercept for county
- $S_{t''}(t)$: the value of natural spline basis t'' on year, evaluated at year t
- $S_{a''}(a)$: the value of natural spline basis a'' on age, evaluated at age group a
- $\boldsymbol{\beta}_{2,t''}$: the vector of fixed effect regression coefficients corresponding to the natural spline basis for time
- $\boldsymbol{\beta}_{3,a''}$: the vector of fixed effect regression coefficients corresponding to the natural spline basis for age group

- $\gamma_{3,a,r,e,m}$: random intercept for county, age group, racial and/or ethnic population, educational attainment, and marital status
- $\gamma_{4,r,d|d\neq BRFSS\_LLCP}$: random intercept for race and data source (not evaluated for the BRFSS combined landline and cell phone sample, which is the gold-standard source)
- $\gamma_{5,a,d|d\neq BRFSS\_LLCP}$: random intercept for age and data source (not evaluated for the BRFSS combined landline and cell phone sample)

The following prior distributions were assigned for each random component in the above model:

- Random effects on racial and/or ethnic population, marital status, or data source (ie, the relevant components of γ_1, γ_3, γ_4, and γ_5) were assumed to follow independent and identically distributed (IID) mean-0 Normal distributions, with variation specified by hyperparameters σ.
- Effects on county, year, age group, or educational attainment (ie, the relevant components of γ_1, γ_2, γ_3, and γ_5) were assumed to follow conditional autoregressive distributions (specifically, LCAR, as described in section **Error! Reference source not found.**), with adjacency between neighbouring counties, consecutive years, age groups, and educational attainment categories (Less than High School, High School, Some College, BA or Higher), respectively. Unlike other model terms on age and year, γ_1 used conditional autoregressive distributions on age and year linear spline basis functions, rather than on the full age and year indices.
- Fixed effects were used on the natural spline basis functions on year and age in $\boldsymbol{\beta}_2$ and $\boldsymbol{\beta}_3$, respectively, because, unlike in the case of linear basis splines, the natural spline basis functions do not correspond directly to "neighbouring" time or age groups.
- Random effects consisting of interactions among two or more dimensions (ie, γ_1, γ_3, γ_4, and γ_5) were assumed to follow mean-0 multivariate Normal distributions with separable covariance structures defined via Kronecker products of the precision matrices of a combination of IID or LCAR priors. These random intercept terms were associated with hyperparameters for variation (σ).

Finally, hyperpriors were defined for the standard deviation (σ) and autocorrelation (ρ) hyperparameters:

- Penalised complexity (PC) priors[43] were specified for the inverse variance ($1/\sigma^2$) of each random effect (described in section) with parameters $\sigma_0 = 5; \Pr(\sigma > \sigma_0) = 0.05$.
- $\mathrm{Normal}(0, \mathrm{variance} = 1.5^2)$ priors were specified for the logit-transformation of the correlation parameters (ρ).

These model specifications defined the log-odds ratio of underlying prevalence (p) as a function of covariates and county, year, age, racial and/or ethnic population, educational attainment, marital status, and data source. We selected the covariates included in this model—poverty rate, proportion born outside the US, and population density—based on data availability and their previously observed associations with health. We specified random effects to capture location- and population-specific patterns in the data while pooling information across strata to stabilise estimates. For example, γ_1 allowed for spatial (ie, between-county) variation in prevalence, shared across age, year, and racial and/or ethnic population. This particular random intercept incorporated a linear spline in the age and time dimensions to reduce computational complexity; the equivalent model for all age groups and years was found to be computationally infeasible in earlier analyses.[47] We chose the numbers of age and year knots to maximise flexibility while maintaining reasonable model runtimes. Although the splines in this random intercept were linear, we did not assume that the time or age trends for $\text{logit}(p)$ are linear, as the contributions from the covariates and natural spline main effect on time in other model terms allowed for non-linear variation.

2.4.3 Addressing data misalignment

Our prevalence models were designed to produce prevalence estimates simultaneously for all combinations of 3,110 counties, 14 age groups, twenty years, five racial and/or ethnic populations, four educational attainment categories, three marital status categories, and two sexes. Modelling variation at this high degree of stratification ultimately required model inputs to be collectively stratified to that same level of resolution. Survey data for the present study were available from BRFSS and Gallup with the demographic detail that we required for modelling prevalence in most dimensions, but there were two primary exceptions which required additional methodological development. One of these exceptions was spatial: as previously described (section 2.2), the BRFSS data to which we had access were variously reported by county, CBSA, metropolitan division, or state, necessitating the development of synthetic spatial units (CBSA-counties) whose boundaries corresponded with those of one or more aggregated counties. The second exception was related to age groups: starting in 2013, BRFSS used ages 80+ as the oldest age group for reporting purposes, rather than 85+ (the latter being the oldest age group in our analysis).

We used disaggregation regression[48-50] to incorporate aggregated inputs during maximum likelihood optimization. In this approach, the prevalence of an aggregate stratum was assumed to be the population-weighted average of its constituent strata. For example, the estimated prevalence in a

hypothetical CBSA-county, composed of two counties and one CBSA, would be the average prevalence estimated from the model linear predictor for each of the two counties, weighted by their populations. When fitting the model in TMB, the link-transformed linear predictor (ie, prevalence estimate) would be calculated for a given demographic stratum within each of the two counties, and these estimates would be weighted by the respective populations in each county-stratum. Finally, the negative log likelihood was calculated for the aggregated input data (eg, cases in the CBSA), sample size, and the combined prediction from constituent strata. This process is generalised as follows, for an aggregate stratum k consisting of g constituent strata:

$$Y_k \sim \text{Binomial}(p_k, n_k) \tag{8}$$

$$p_k = \frac{\sum_{i=1}^{g}(p_i \cdot w_i)}{\sum_{i=1}^{g} w_i} \tag{9}$$

$$n_k = \sum_{i=1}^{g} n_i \tag{10}$$

where w_i is the proportion of the population in k that is represented by stratum i, Y_k and n_k are the cases and sample size, respectively, across all constituent strata, and p_i is the prevalence estimate for a constituent stratum. Population weights were derived from the post-stratification frame (section 2.4.4). Disaggregation regression is highly flexible in its application, being agnostic of the dimensionality of the aggregation within the input data. We used this approach for both spatially-aggregated and age-aggregated inputs in the prevalence models, as described above.

In our study, variation at the county level in models using BRFSS data was facilitated by county-level covariates, partial availability of county-level resolution in BRFSS in some states and years, and joint modelling with Gallup data (which uniformly had county identifiers). The inclusion of aggregate age group 80+ data in the inputs to models for BRFSS variables likely had only minor impacts on accuracy and precision because BRFSS disaggregated data for ages 80–84 and 85+ prior to 2013, and Gallup disaggregated data for ages 80–84 and 85+ in all years it was available (2008–2017).

An important assumption underlying our use of disaggregation regression for survey data is that the combination of sampled individuals from constituent strata are population-representative of the larger aggregate stratum. This assumption requires that the sampling rates among constituent strata, appropriately weighted to account for survey design and non-response, are proportional to their populations. Such a scenario is unlikely to be realised exactly in most instances, with the consequent

potential for bias to enter our model estimates. We acknowledge this as a limitation of our approach but believe that excluding the aggregate data would likely introduce greater bias or loss of precision.

2.4.4 Post-stratification frame

We used multilevel regression with post-stratification (MrP)[51-53] to adjust for non-response bias in survey data. This procedure adjusts final model estimates by weighting modelled strata, such that they have the same demographic distribution as the target population. While sample weights are commonly used to adjust for non-response bias in survey data, sample weights were calculated differently in our data sources (BRFSS and Gallup) and even within the same data source over time (eg, BRFSS added a cell phone sample frame and redesigned its weighting methodology in 2011).[44] These inconsistencies in survey weights limit the comparability of temporal and combined trends by data source.[44] Moreover, these surveys adjusted the survey weights only to sub-state regions and states, and were not designed to adjust for sampling bias at the county level.

As described in section 2.4.2, we included educational attainment and marital status as dimensions in our models. After model-fitting, we generated predictions at the full stratification present in the models, then collapsed predictions across educational attainment and marital status to derive final estimates by county, age, sex, year, and race and/or ethnicity. This aggregation was performed by taking the weighted average of the education and marital-specific estimates, using population estimates from the post-stratification frame as weights.

In the absence of county-level population data simultaneously stratified across all the dimensions of our final estimates, in addition to educational attainment and marital status, we needed to estimate the detailed population distribution (ie, the post-stratification frame) to be able to apply the MrP method. The post-stratification frame estimation method was adapted from Leeman et al,[54] with some technical alterations such as the use of raking to allow for the multi-dimensional split of population data.

The steps we used (iterative proportional fitting) to produce the post-stratification frame can be summarized as follows: (1) compiling and adjusting the population data to serve as raking margins; (2) obtaining a raw initial population distribution; and (3) raking the initial distribution to the known population margins to obtain the post-stratification frame.

Compiling the raking margins: We obtained population estimates from ACS at the county level by age, sex, year, and each of the following variables separately: racial and/or ethnic population, marital status, and educational attainment. Additionally, we used the population distribution from IPUMS at the state

and national levels stratified jointly by age, sex, racial and/or ethnic population, educational attainment, and marital status, by year. Of note, one condition for raking is that the marginal distributions should sum to the same total overall and across overlapping margins (eg, age and sex). This was not the case for the marginal distributions we used, given differences in data sources, suppression rules at the county level, and data processing. Thus, we opted to scale the county level marginal distributions so that they sum to the state-level distribution across all overlapping variables (ie, age, sex, racial and/or ethnic population, marital status, educational attainment, and year).

Obtaining an initial population distribution: We obtained an initial joint distribution frame by applying the same joint distribution observed at the state level to all counties in a state adjusted to the county population size by age, sex, and race.

Raking: We then raked the joint distribution iteratively by each of the county, state, and national population marginal distributions listed above, until convergence was achieved or after 40 iterations. Convergence was defined as having a maximum difference of 10^{-5} between the sum of the joint cells and each of the raking marginal distributions. The resulting frame had 5,224,800 strata per year and was vetted to ensure alignment with each of the marginal distributions in all years.

2.5 Covariates

The small-area estimation models used in the present study leveraged observed relationships between modelled outcomes and sociodemographic and socioeconomic factors (covariates) to improve estimates for strata lacking robust outcome data. The underlying data for the covariates stratified by both county and racial and/or ethnic population contained missing values and displayed instability and low precision for some strata with small populations. We used small-area estimation models to smooth covariate estimates in all years, counties, and racial and/or ethnic populations prior to including these covariates in overweight and obesity models.

We fitted small-area estimation models to estimate poverty rate and percentage born outside the US, stratified by race and/or ethnicity, using tabulated data from the 2000 decennial population census and the American Community Survey (ACS, 5-year files from 2010 to 2022, with midpoints treated as data years for modelling). The data processing and modelling approach was identical for these covariates and is described in detail below. We also used population density by county (Table S3), which was incorporated in these models in its original (not modeled) form due to its complete spatial and temporal coverage.

Decennial census data (2000)

We aggregated census data on poverty rate and birth outside the US across sex to obtain summed counts (populations in poverty or with birth outside the US, and their total population denominators) for 2000 by county and racial and/or ethnic population. We similarly combined estimates for the Asian and NHPI populations using summed counts.

The long-form decennial census questionnaire was completed by a 17% sample of the population in 2000.[55] We therefore assumed sample sizes ($\widehat{\mathrm{N}}$) to be 17% of the total population for each combination of county, year, and racial and/or ethnic population in 2000.

American Community Survey data

Observations for years 2008–20 were aggregated as described above for the 2000 census data. Due to instability in effective sample sizes calculated from ACS-provided margin of error estimates, we derived sample sizes ($\widehat{\mathrm{N}}$) by multiplying annual national ACS sampling rates by stratum-specific population estimates; case counts were then calculated by multiplying these sample sizes by observed stratum-specific prevalence estimates.

Small-area estimation of proportions

We modelled proportions in poverty or with birth outside the US using small-area estimation models with the following model specification:

$$Y_{j,k,t,r} \sim \mathrm{Binomial}\left(p_{j,k,t,r}, n_{j,k,t,r}\right) \tag{11}$$

$$\mathrm{logit}\left(p_{j,k,t,r}\right) = \boldsymbol{\beta}_0 + \gamma_{1,j} + \gamma_{2,j,t,r} + \gamma_{3,k,t,r} \tag{12}$$

Priors:

$\gamma_1 \sim \mathrm{LCAR}(\rho_{1,j}, \sigma_1)$

$\gamma_2 \sim \mathrm{IID:LCAR:IID}\left(\rho_{2,t}, \sigma_2\right)$

$\gamma_3 \sim \mathrm{IID:LCAR:IID}(\rho_{2,t}, \sigma_3)$

Hyperpriors:

$\sigma^{-2} \sim \mathrm{PC}(\sigma_0 = 5, \alpha = 0.05) \rightarrow \Pr(\sigma > 5) = 0.05$

$\mathrm{logit}(\rho) \sim \mathrm{Normal}(0, \mathrm{variance} = 1.5^2)$

where $Y_{j,k,t,r}$ is the cases in county j, state k, year t, and racial and/or ethnic population r among a sample of n individuals with prevalence p. Model terms consist of:

- β_0: global intercept
- $\gamma_{1,j}$: random intercept for county
- $\gamma_{2,j,t,r}$: random intercept for county, year, and racial and/or ethnic population
- $\gamma_{3,k,t,r}$: random intercept for state, year, and racial and/or ethnic population

The following prior distributions were assigned for each random and fixed component:

- Effects on county in γ_1 and on year in γ_2 and γ_3 were assumed to follow conditional autoregressive distributions of the form described by Leroux, Lei, and Breslow (known as an LCAR prior).[56] LCAR priors correspond with the following full conditional distribution for each individual element, for example, for effects by county j:

$$\gamma_j | \gamma_{k\sim j}, \sigma^2, \rho \sim \text{Normal}\left(\frac{\rho \cdot \sum_{k\sim j} \gamma_k}{n_j \cdot \rho + 1 - \rho}, \frac{\sigma^2}{n_j \cdot \rho + 1 - \rho}\right) \quad (13)$$

  where $k \sim j$ indicates the set of counties that are adjacent to county j and n_j is the number of counties in $k \sim j$. In this distribution, the σ^2 parameter controls the degree of spatial variation and the ρ parameter, which varies between 0 and 1, represents correlation between neighbours. Connections between counties were determined by geographic adjacency, with adjacency enforced manually for some geographically isolated locations to stabilise estimates: Nantucket Island (connected to Barnstable County, MA), Hawai'i (Honolulu County was connected to San Diego County, CA), and Alaska (Anchorage Municipality was joined to King County, WA). The analogous LCAR specification is used for effects on year, with adjacency between consecutive years.
- Random effects on county, state, or racial and/or ethnic population in γ_2 and γ_3 were assumed to follow independent and identically distributed (IID) mean-0 Normal distributions.
- Random effects consisting of interactions among two or more dimensions (ie, γ_2 and γ_3) were assumed to follow mean-0 multivariate Normal distributions with separable covariance structures defined via Kronecker products of the precision matrices of a combination of IID or LCAR priors. These random intercept terms were associated with hyperparameters for variation (σ).

Finally, hyperpriors were defined for the standard deviation (σ) and autocorrelation (ρ) hyperparameters:

- Penalised complexity (PC) priors were specified for the inverse variance ($1/\sigma^2$) of each random effect.[43] PC priors shrink toward a base model, which here is a marginal variance of 0. They are specified by setting the tail probability on each hyperparameter. We set $\sigma_0 = 5; \Pr(\sigma > \sigma_0) = 0.05$.
- $\text{Normal}(0, \text{variance} = 1.5^2)$ priors were specified for the logit-transformation of the correlation parameters (ρ).

These model specifications define the log-odds ratio of underlying prevalence (p) as a function of county, state, year, and racial and/or ethnic population. We fitted these models with the Template Model Builder (TMB) package[57] in R version 3.6.1[58] using an empirical Bayes approach. TMB calculates analytic approximations to the posterior distribution based on Laplace approximations.

2.6 Population distribution of BMI

The health risks and benefits associated with BMI exist on a continuum. We generated the continuous population distribution of BMI to calculate population attributable fractions (PAFs). We estimated the population distribution of BMI separately by age, sex, race, year, and county in three steps. First, we estimated the mean BMI of a population based on the prevalence of overweight and obesity. Next, we characterised the shape of the population distribution of BMI as a mixture of known probability distributions, which we call the "ensemble distribution."[1] Lastly, we calculated the optimal population variance of BMI for each stratum so that the resulting ensemble distribution matched the previously estimated mean BMI and prevalence of overweight and obesity. This general process is described in more depth in the Global Burden of Diseases, Injuries, and Risk Factors Study (GBD) 2021 and Fitzgerald et al.[1,59]

2.6.1 Mean BMI

We estimated mean BMI indirectly through prevalence estimation. We first collapsed the adjusted individual-level observations of BMI to derive estimates of mean BMI by state, sex, year, age, and racial and/or ethnic population using survey weights. Separate collapsed datasets were produced for each of ten imputations of self-report adjusted BMI. Due to the high frequency of observed strata with only one or a few sampled individuals, stratum-specific BMI standard error estimates were unstable and unreliable and were not used in the modelling process; instead, we used sample sizes to scale stratum-specific Gaussian precisions during model fitting (see below).

We fitted Bayesian models of mean BMI in R-INLA,[42] with separate models by sex, using the following specification:

$$Y_{k,t,r} \sim \text{Normal}\left(\mu_{k,t,r}, \frac{\sigma}{\sqrt{N_{k,t,r}}}\right) \tag{14}$$

$$\log(\mu_{k,t,r}) = \beta_0 + \gamma_{1,b,r} + \gamma_{2,v,r} + \gamma_{3,t,r} \tag{15}$$

where $Y_{k,t,r}$ is the estimated mean BMI in state *k*, year *t*, and racial and/or ethnic population *r*, N is sample size (used to scale precision), *b* is obesity category, and *v* is overweight category. Obesity and overweight prevalences were discretised into 24 bins by quantile. Model terms consisted of:

- β_0: global intercept
- $\gamma_{1,b,r}$: random intercept for obesity prevalence bin with a second-order random walk (RW2) prior, replicated by racial and/or ethnic population
- $\gamma_{2,v,r}$: random intercept for overweight prevalence bin with a second-order random walk (RW2) prior, replicated by racial and/or ethnic population
- $\gamma_{3,t,r}$: random intercept for year, with a first-order autoregressive (AR1) prior, replicated by racial and/or ethnic population

Default INLA priors were used for the parameters and hyperparameters: a Normal (mean = 0.0, precision = 0.0) prior was used for the global intercept, while the RW2 models had Gamma (shape = 1.0, inverse-scale = 5×10^{-5}) priors on precision*.* These default priors were used as we considered them suitably weak in the absence of *a priori* information with which to establish more informative priors. A Gaussian approximation strategy and empirical Bayes integration strategy were used for model fitting.

We took 100 draws from the estimated joint posterior distribution of the fixed and random effects for each of the ten fitted models. These parameter draws were then used to build predictions of mean BMI by county, year, sex, age, and racial and/or ethnic population, using draws of obesity and overweight prevalence from the small-area estimation models (section 2.4).

2.6.2 Ensemble weights

We applied a previously-developed approach for estimating the population distribution of BMI based on the population mean and variance of BMI.[1,59] The ensemble distribution is the weighted average of the

probability density functions of each component distribution. We considered the following distributions: Gamma, inverse-gamma, log-Logistic, Gumble, Weibull, inverse-Weibull, log-normal, mirrored Gamma, mirrored Gumble, and Beta (with a location-scale transformation). The parameters of each distribution were solved from the population mean and variance.

We optimised the weight on each distribution to fit the age, sex, and race-specific distributions of measured BMI from NHANES. Following the approach in Fitzgerald,[59] we ran the optimiser with 100 different starting sets of ensemble weights. We optimized the set of weights that maximised goodness of fit on the right side of the BMI distribution. Goodness of fit was defined as the sum of squared errors of the prevalence of overweight and obesity and obesity alone implied by the ensemble distribution relative to the prevalences based on NHANES microdata. We optimised the distribution around the overweight and obesity thresholds to prioritise parts of the distribution that are common and associated with excess risk of mortality, which is most relevant for accurately estimating the PAF. As a sensitivity analysis, we included the underweight BMI threshold in the optimiser and found that optimising the fit around the high end of the BMI distribution did not substantially sacrifice fit on the left side of the distribution. We fitted different sets of ensemble weights by sex, race and/or ethnicity, and age group to reflect different population distribution shapes. The weights are shown in Table S8.

2.6.3 Standard deviation optimisation

The ensemble weights defined the form of the BMI distribution in the population, but the distribution for BMI in each stratum was estimated separately based on the population mean and standard deviation of BMI. For each stratum, we optimised the population standard deviation to generate a distribution that best fitted the combination of overweight and obesity, obesity, and mean BMI estimates. We optimized the population standard deviation to minimise the sum of squared errors in the prevalence of overweight and obesity implied by the ensemble distribution compared to the same values from the small-area estimation models, holding mean BMI constant at the previously estimated level.

2.7 Calculation of Attributable Burden

Mortality attributable to non-optimal BMI was estimated using the comparative risk assessment (CRA) framework used in GBD 2021.[1,60] In short, we estimated the rates of YLLs that could have been avoided if the population distribution of BMI was brought to the level associated with the lowest mortality. There are three main components to this calculation.

2.7.1 Relative risk curves and theoretical minimum risk exposure level

We utilised the risk-outcome relationships from GBD 2021 to estimate the PAFs in this study. Dose-response relationships between non-optimal BMI and causes of death were identified and extracted through separate systematic reviews. The dose-response curve, with between-study heterogeneity, was estimated using a flexible Bayesian meta regression model. The theoretical minimum risk exposure level (TMREL) is the level of BMI associated with the lowest all-cause mortality, which was estimated to be uniformly distributed between 20 and 21 kg/m^2 in GBD 2021.

2.7.2 Population attributable fraction

We calculated the PAF for each cause of mortality associated with non-optimal BMI. The PAF formula, adapted from GBD 2021, is:

$$PAF_{c,a,s,r,j,t} = \frac{\int_{x=10}^{50} RR_{c,a,s}(x) P_{a,s,r,j,t}(x) dx - RR_{c,a,s}(TMREL)}{\int_{x=10}^{50} RR_{c,a,s}(x) P_{a,s,r,j,t}(x) dx} \tag{16}$$

where $PAF_{j,t,a,r,\mathrm{s},\mathrm{c}}$ is the PAF for cause c, age group a, sex s, race and/or ethnicity r, county j, and year t. $RR_{c,a,s}(x)$ is the relative risk of cause c at a BMI of x for age group a and sex s, which is multiplied by $P_{a,s,r,j,t}$, the probability density function of BMI exposure at x for age group a, sex s, race and/or ethnicity r, county j, and year t. Following the approach in GBD 2021, $RR_{c,a,s}(x)$ was modified so that the relative risk for BMI values below the TMREL were set equal to the relative risk at the TMREL. We truncated the BMI distribution to the interval from 10 to 50, representing almost the entire distribution of BMI observed in NHANES; the density function for the distribution was rescaled after truncation to ensure that it integrated to one over the support. The product of the relative risk and BMI distribution was integrated from 10 to 50. PAFs were only directly computed for the most detailed demographic strata.

2.7.3 Attributable burden

Attributable burden (AB) is the amount of disease burden that would have been avoided if the population-level BMI was reduced to the TMREL. We estimated attributable burden using the following formula:

$$AB_{c,a,s,r,j,t} = PAF_{c,a,s,r,j,t} \times YLL_{c,a,s,r,j,t} \tag{17}$$

Where AB for cause c for age group a, sex s, race and/or ethnicity r, county j, and year t was the product of the PAF and YLLs for the corresponding populations. AB was directly calculated for the most-detailed

27 causes of YLLs that we estimated; for aggregate causes (eg, all cardiovascular diseases combined, which is composed of more-detailed causes such as ischemic heart disease and hypertensive heart disease), we summed up the attributable burden for specific causes. Similarly, attributable burden for aggregate populations (eg, all counties combined) were summed up from the most-detailed categories. Aggregate PAFs were back-calculated by dividing aggregated attributable YLL rates by the combined rate of attributable and not attributable YLLs.

Supplementary Tables

Table S1. Cause hierarchy for attributable burden

Cause Name	Level
All causes	0
Non-communicable diseases	1
Neoplasms	2
Colon and rectum cancer	3
Liver cancer	3
Gallbladder and biliary tract cancer	3
Pancreatic cancer	3
Breast cancer	3
Uterine cancer	3
Ovarian cancer	3
Kidney cancer	3
Thyroid cancer	3
Non-Hodgkin's lymphoma	3
Burkitt lymphoma	4
Other non-Hodgkin lymphoma	4
Multiple myeloma	3
Leukaemia	3
Acute lymphoid leukaemia	4
Chronic lymphoid leukaemia	4
Acute myeloid leukaemia	4
Chronic myeloid leukaemia	4
Other leukaemia	4
Cardiovascular diseases	2
Ischaemic heart disease	3
Stroke	3
Ischaemic stroke	4
Intracerebral hemorrhage	4
Subarachnoid hemorrhage	4
Hypertensive heart disease	3

Cause Name	Level
Atrial fibrillation and flutter	3
Chronic respiratory diseases	2
Asthma	3
Digestive diseases	2
Gallbladder and biliary diseases	3
Neurological disorders	2
Alzheimer's disease and other dementias	3
Diabetes and kidney diseases	2
Diabetes mellitus	3
Diabetes mellitus type 2	4

GBD 2021 cause hierarchy, restricted to causes estimated in the present analysis.

Table S2. Counties combined to create historically stable units of analysis

State	Group	Counties (FIPS code)
Alaska	1	Chugach Census Area (2063), Copper River Census Area (2066), Valdez-Cordova Census Area (2261)*
Alaska	2	Kusilvak Census Area (2158), Wade Hampton Census Area (2270)*
	3	Kobuk Census Area (2140)*, Northwest Arctic Borough (2188)
	4	Aleutian Islands Census Area (2010)*, Aleutians East Borough (2013), Aleutians West Census Area (2016)
	5	Dillingham Census Area (2070), Lake and Peninsula Borough (2164)
	6	Denali Borough (2068), Yukon-Koyukuk Census Area (2290)
	7	Hoonah-Angoon Census Area (2105), Skagway Municipality (2230), Skagway-Yakutat-Angoon Census Area (2231)*, Skagway-Hoonah-Angoon Census Area (2232)*, Yakutat City and Borough (2282)
	8	Ketchikan Gateway Borough (2130), Petersburg Borough (2195), Prince of Wales-Hyder Census Area (2198), Prince of Wales-Outer Ketchikan Census Area (2201)*, Wrangell City and Borough (2275), Wrangell-Petersburg Census Area (2280)*
Arizona	1	La Paz County (4012), Yuma County (4027)
Colorado	1	Adams County (8001), Arapahoe County (8005), Boulder County (8013), Broomfield County (8014), Denver County (8031), Jefferson County (8059), Weld County (8123)
Florida	1	Dade County (12025)*, Miami-Dade County (12086)
Hawaii	1	Kalawao County (15005), Maui County (15009)
Maryland	1	Montgomery County (24031), Prince George's County (24033)
Montana	1	Park County (30067), Yellowstone National Park (30113)*
New Mexico	1	Cibola County (35006), Valencia County (35061)
South Dakota	1	Oglala Lakota County (46102), Shannon County (46113)*
	2	Jackson County (46071), Washabaugh County (46131)*
Virginia	1	Fairfax County (51059), Fairfax City (51600)
	2	Rockingham County (51165), Harrisonburg City (51660)
	3	James City County (51095), Williamsburg City (51830)
	4	Prince William County (51153), Manassas City (51683), Manassas Park City (51685)
	5	Rockbridge County (51163), Buena Vista City (51530)
	6	Spotsylvania County (51177), Fredericksburg City (51630)
	7	Augusta County (51015), Staunton City (51790), Waynesboro City (51820)
	8	Pittsylvania County (51143), Danville City (51590)
	9	Greensville County (51081), Emporia City (51595)
	10	Albemarle County (51003), Charlottesville City (51540)
	11	Bedford County (51019), Bedford City (51515)*
	12	Halifax County (51083), South Boston City (51780)*
	13	Southampton County (51175), Franklin City (51620)
	14	Alleghany County (51005), Clifton Forge City (51560)*
	15	York County (51199), Newport News City (51700)

*County no longer exists due to boundary or name change.
FIPS = Federal Information Processing Standards.

Table S3. Covariate data sources

Covariate	Data sources	Data processing	Citations
Population density	2000–2019 NCHS bridged race files [1–2]; 2020 cartographic boundary file, state-county for United States [3] accessed using the tigris package [4]	The area of each county was calculated using an Albers Equal Area Conic projection. The total population of each county was divided by the total area of the county and was then log-transformed.	[1] National Center for Health Statistics, Centers for Disease Control and Prevention, US Census Bureau. United States Bridged-Race Intercensal Population Estimates 2000–2009. Hyattsville, United States: National Center for Health Statistics, Centers for Disease Control and Prevention, 2012. https://www.cdc.gov/nchs/nvss/bridged_race.htm. Accessed October 30, 2012.<br><br>[2] National Center for Health Statistics, Centers for Disease Control and Prevention, US Census Bureau. United States Vintage 2020 Bridged-Race Postcensal Population Estimates 2010–2020. Hyattsville, United States: National Center for Health Statistics, Centers for Disease Control and Prevention, 2020. https://www.cdc.gov/nchs/nvss/bridged_race.htm. Accessed February 17, 2022.<br><br>[3] US Census Bureau. TIGER/Line Shapefile, 2020 Cartographic Boundary File, State-County for United States, 1:20,000,000. https://www.census.gov/geographies/mapping-files/time-

Covariate	Data sources	Data processing	Citations
			series/geo/tiger-line-file.2020.html#list-tab-790442341. Accessed October 12, 2022.<br><br>[4] Walker K (2022). *tigris: Load Census TIGER/Line Shapefiles*. R package version 2.0, <https://github.com/walkerke/tigris>.
Percent of the population below the poverty line by race and/or ethnicity	1990 census [5]; 2000 census [6]; 2010–2022 ACS [7]	ACS estimates for American Indian or Alaskan Native (AIAN), Asian or Pacific Islander (Asian), and Black were not available stratified by Latino ethnicity and were used as proxies for AIAN, Asian, and Black, respectively. Imputation via a small-area estimation model was used to generate and smooth missing values.	[5] Minnesota Population Center. 1990 Census Summary Tape File 4, Table NPB100. IPUMS National Historical Geographic Information System: Version 15.0. Minneapolis, MN: IPUMS 2020. https://www.nhgis.org/. Accessed August 30, 2020.<br><br>[6] Minnesota Population Center. 2000 Census Summary File 4, Table NPCT142A. IPUMS National Historical Geographic Information System: Version 15.0. Minneapolis, MN: IPUMS 2020. https://www.nhgis.org/. Accessed August 30, 2020.<br><br>[7] US Census Bureau. American Community Survey, 2010–2022 American Community Survey 5-Year Estimates, Tables B17001A-B17001I; using Census data portal; https://data.census.gov/cedsci/. Accessed March 6, 2024.

Covariate	Data sources	Data processing	Citations
Percent of the population that is born outside the US by race and/or ethnicity	1990 census [8]; 2000 census [9]; 2010–2022 ACS [10]	ACS estimates for American Indian or Alaskan Native (AIAN), Asian or Pacific Islander (Asian), and Black were not available stratified by Latino ethnicity and were used as proxies for AIAN, Asian, and Black, respectively. Imputation via a small-area estimation model was used to generate and smooth missing values.	[8] Minnesota Population Center. 1990 Census Summary Tape File 4, Table NPB28. IPUMS National Historical Geographic Information System: Version 15.0. Minneapolis, MN: IPUMS 2020. https://www.nhgis.org/. Accessed October 8, 2020.<br>[9] Minnesota Population Center. 2000 Census Summary File 4, Table NPCT043A. IPUMS National Historical Geographic Information System: Version 15.0. Minneapolis, MN: IPUMS 2020. https://www.nhgis.org/. Accessed October 8, 2020.<br>[10] US Census Bureau. American Community Survey, 2010-2022 American Community Survey 5-Year Estimates, Tables B05003A-B05003I; using Census data portal; https://data.census.gov/cedsci/. Accessed March 6, 2024.

Covariate	Data sources	Data processing	Citations
Percent land on federal reservations	2012 TIGER/Line shape files [11]; 2013 TIGER/Line shape files [12]	Using ArcPy, the 2012 Native areas shape file was overlaid on the 2013 US county boundary file to determine overlap between federal reservations and counties.	[11] US Census Bureau. 2012 TIGER/Line Shapefiles: American Indian Area Geography. https://www.census.gov/cgi-bin/geo/shapefiles/index.php. Accessed March 5, 2015.<br>[12] US Census Bureau. 2013 TIGER/Line Shapefiles: Counties (and equivalent). https://www.census.gov/cgi-bin/geo/shapefiles/index.php. Accessed March 5, 2015.

Table S4. Population data sources

Data source and citation	Description
National Center for Health Statistics, Centers for Disease Control and Prevention, US Census Bureau. United States Bridged-Race Intercensal Population Estimates 2000-2009. Hyattsville, United States: National Center for Health Statistics, Centers for Disease Control and Prevention, 2012. https://www.cdc.gov/nchs/nvss/bridged_race.htm. Accessed October 30, 2012.	Population estimates by county, sex, age, and race and/or ethnicity for 2000-2009, used in the creation of the post-stratification frame and for aggregating estimates.
National Center for Health Statistics, Centers for Disease Control and Prevention, US Census Bureau. United States Vintage 2020 Bridged-Race Postcensal Population Estimates 2010-2020. Hyattsville, United States: National Center for Health Statistics, Centers for Disease Control and Prevention, 2020. https://www.cdc.gov/nchs/nvss/bridged_race.htm. Accessed February 17, 2022.	Population estimates by county, sex, age, and race and/or ethnicity for 2010-2020, used in the creation of the post-stratification frame and for aggregating estimates.
Steven Ruggles, Sarah Flood, Matthew Sobek, Danika Brockman, Grace Cooper, Stephanie Richards, and Megan Schouweiler. IPUMS USA: Version 13.0 American Community Survey 1-Year Files, 2009–2021. Minneapolis, MN: IPUMS, 2023. https://doi.org/10.18128/D010.V13.0. Accessed March 8, 2023.	Population by PUMA, sex, age, and educational attainment or marital status for 2007-2019, used in the creation of the post-stratification frame.
Minnesota Population Center. 2000 Census Summary File 3, Table PCT025. IPUMS National Historical Geographic Information System: Version 15.0. Minneapolis, MN: IPUMS 2020. https://www.nhgis.org/. Accessed September 15, 2020.	Population by county, sex, broader age groups (25-34, 35-44, 45-64, 65+), and educational attainment or marital status for 2000, used in the creation of the post-stratification frame.
Steven Ruggles, Sarah Flood, Matthew Sobek, Danika Brockman, Grace Cooper, Stephanie Richards, and Megan Schouweiler. IPUMS USA: Version 13.0 IPUMS Census 5% Sample, 2000. Minneapolis, MN: IPUMS, 2023. https://doi.org/10.18128/D010.V13.0. Accessed August 23, 2022.	Population by PUMA, sex, age, and educational attainment or marital status for 2000, used in the creation of the post-stratification frame.

Data source and citation	Description
US Census Bureau. American Community Survey, 2009–2021 American Community Survey 5-Year Estimates, Table B15001; using Census data portal; https://data.census.gov/cedsci/. Accessed April 26, 2023.	Population by county, sex, broader age groups (25-34, 35-44, 45-64, 65+), and educational attainment for 2007-2019, used in the creation of the post-stratification frame.
US Census Bureau. American Community Survey, 2009–2021 American Community Survey 5-Year Estimates, Table B12002; using Census data portal; https://data.census.gov/cedsci/. Accessed April 26, 2023.	Population by county, sex, broader age groups (25-34, 35-44, 45-64, 65+), and marital status for 2007-2019, used in the creation of the post-stratification frame.

Table S5. Sources of survey data

Title	Years covered	Citation
Alaska Behavioral Risk Factor Surveillance System	2014–2019	Alaska Department of Health, Division of Public Health, Section of Chronic Disease Prevention and Health Promotion, Centers for Disease Control and Prevention (CDC). United States – Alaska Behavioral Risk Factor Surveillance System 2014–2019. Anchorage, AK, United States of America: Alaska Department of Health and Social Services.
Arizona Behavioral Risk Factor Surveillance System	2006, 2007, 2009–2019	Arizona Department of Health Services (ADHS), Centers for Disease Control and Prevention (CDC). United States – Arizona Behavioral Risk Factor Surveillance System 2006, 2007, 2009–2019. Phoenix, AZ, United States of America: Arizona Department of Health Services (ADHS).
Arkansas Behavioral Risk Factor Surveillance System	2011–2019	Arkansas Department of Health, Centers for Disease Control and Prevention (CDC). United States – Arkansas Behavioral Risk Factor Surveillance System 2011–2019. Little Rock, United States of America: Arkansas Department of Health.
California Behavioral Risk Factor Surveillance System	2013–2019	California Behavioral Risk Factor Surveillance System Program, California Department of Public Health, Centers for Disease Control and Prevention (CDC), Public Health Survey Research Program (California State University, Sacramento). United States – California Behavioral Risk Factor Surveillance System 2013–2019.
Connecticut Behavioral Risk Factor Surveillance System	2013–2019	Centers for Disease Control and Prevention (CDC), Connecticut Department of Public Health. United States – Connecticut Behavioral Risk Factor Surveillance System 2013–2019.

Title	Years covered	Citation
Delaware Behavioral Risk Factor Surveillance System	2004–2019	Delaware Division of Public Health (United States), Delaware Health and Social Services. United States – Delaware Behavioral Risk Factor Surveillance System 2004–2019.
Florida Behavioral Risk Factor Surveillance System	2007, 2010, 2013, 2016	Centers for Disease Control and Prevention (CDC), Florida Department of Health. United States – Florida Behavioral Risk Factor Surveillance System 2007, 2010, 2013, 2016. Tallahassee, FL, United States of America: Florida Department of Health.
Gallup Daily	2008–2017	Gallup. United States – Gallup Daily 2008–2017. Washington, D.C., United States of America: Gallup.
Hawaii Behavioral Risk Factor Surveillance System	2005–2019	Centers for Disease Control and Prevention (CDC), Hawaii State Department of Health. United States – Hawaii Behavioral Risk Factor Surveillance System 2005–2019.
Illinois Behavioral Risk Factor Surveillance System	2000–2019	Centers for Disease Control and Prevention (CDC), Illinois Department of Public Health. United States – Illinois Behavioral Risk Factor Surveillance System 2000–2019.
Kansas Behavioral Risk Factor Surveillance System	2001–2019	Centers for Disease Control and Prevention (CDC), Kansas Department of Health and Environment. United States – Kansas Behavioral Risk Factor Surveillance System 2001–2019.
Massachusetts Behavioral Risk Factor Surveillance System	2000–2019	Centers for Disease Control and Prevention (CDC), Massachusetts Executive Office of Health and Human Services (EOHHS). United States – Massachusetts Behavioral Risk Factor Surveillance System 2000–2019.

Title	Years covered	Citation
Mississippi Behavioral Risk Factor Surveillance System	2008–2012	Centers for Disease Control and Prevention (CDC), Mississippi State Department of Health. United States – Mississippi Behavioral Risk Factor Surveillance System 2008–2012. Jackson, MS, United States of America: Mississippi State Department of Health.
New Mexico Behavioral Risk Factor Surveillance System	2000–2019	Centers for Disease Control and Prevention (CDC), New Mexico Department of Health. United States – New Mexico Behavioral Risk Factor Surveillance System 2000–2019.
New York Behavioral Risk Factor Surveillance System	2011–2019	Centers for Disease Control and Prevention (CDC), New York State Department of Health. United States – New York Behavioral Risk Factor Surveillance System 2011–2019.
Ohio Behavioral Risk Factor Surveillance System	2006, 2007, 2013–2019	Centers for Disease Control and Prevention (CDC), Ohio Department of Health. United States – Ohio Behavioral Risk Factor Surveillance System 2006, 2007, 2013–2019.
Pennsylvania Behavioral Risk Factor Surveillance System	2000–2019	Centers for Disease Control and Prevention (CDC), Pennsylvania Department of Health. United States – Pennsylvania Behavioral Risk Factor Surveillance System 2000–2019.
South Dakota Behavioral Risk Factor Surveillance System	2003–2015, 2017–2019	Centers for Disease Control and Prevention (CDC), South Dakota Department of Health. United States – South Dakota Behavioral Risk Factor Surveillance System 2003–2015, 2017–2019.
Texas Behavioral Risk Factor Surveillance System	2007, 2008, 2010, 2011, 2013–2019	Centers for Disease Control and Prevention (CDC), Texas Department of State Health Services. United States – Texas Behavioral Risk Factor Surveillance System 2007, 2008, 2010, 2011, 2013–2019.

Title	Years covered	Citation
United States Behavioral Risk Factor Surveillance System	1999–2020	Centers for Disease Control and Prevention (CDC). United States Behavioral Risk Factor Surveillance System 1999–2020. Atlanta, Georgia: CDC, US Department of Health and Human Services.
United States National Health and Nutrition Examination Survey	1999–2018	National Center for Health Statistics (NCHS), Centers for Disease Control and Prevention (CDC). United States National Health and Nutrition Examination Survey 1999–2018. Hyattsville, United States: National Center for Health Statistics, Centers for Disease Control and Prevention.
United States National Health and Nutrition Examination Survey Pre–Pandemic	2017–2020	National Center for Health Statistics, Centers for Disease Control and Prevention (CDC). United States National Health and Nutrition Examination Survey Pre-Pandemic 2017–2020. Hyattsville, United States of America: National Center for Health Statistics, Centers for Disease Control and Prevention.
United States National Health and Nutrition Examination Survey August 2021–August 2023	2021–2023	National Center for Health Statistics, Centers for Disease Control and Prevention (CDC). United States National Health and Nutrition Examination Survey August 2021–August 2023. Hyattsville, United States of America: National Center for Health Statistics, Centers for Disease Control and Prevention.
Vermont Behavioral Risk Factor Surveillance System	2002–2019	Centers for Disease Control and Prevention (CDC), Vermont Department of Health. United States – Vermont Behavioral Risk Factor Surveillance System 2002–2019.
Virginia Behavioral Risk Factor Surveillance System	2011–2019	Centers for Disease Control and Prevention (CDC), Virginia Department of Health (United States). United States – Virginia Behavioral Risk Factor Surveillance System 2011–2019.

Title	Years covered	Citation
Washington Behavioral Risk Factor Surveillance System	2008	Washington State Department of Health, Center for Health Statistics, Behavioral Risk Factor Surveillance System, supported in part by the Centers for Disease Control and Prevention, Cooperative Agreement U58/CCU022819-5 (2008).
Washington Behavioral Risk Factor Surveillance System	2009	Washington State Department of Health, Center for Health Statistics, Behavioral Risk Factor Surveillance System, supported in part by the Centers for Disease Control and Prevention, Cooperative Agreement U58/DP001996-1 (2009).
Washington Behavioral Risk Factor Surveillance System	2010	Washington State Department of Health, Center for Health Statistics, Behavioral Risk Factor Surveillance System, supported in part by the Centers for Disease Control and Prevention, Cooperative Agreement U58/DP001996-2 (2010).
Washington Behavioral Risk Factor Surveillance System	2011	Washington State Department of Health, Center for Health Statistics, Behavioral Risk Factor Surveillance System, supported in part by the Centers for Disease Control and Prevention, Cooperative Agreement U58/SO000047-1 (2011).
Washington Behavioral Risk Factor Surveillance System	2012	Washington State Department of Health, Center for Health Statistics, Behavioral Risk Factor Surveillance System, supported in part by the Centers for Disease Control and Prevention, Cooperative Agreement U58/SO000047-2 (2012).
Washington Behavioral Risk Factor Surveillance System	2013	Washington State Department of Health, Center for Health Statistics, Behavioral Risk Factor Surveillance System, supported in part by the Centers for Disease Control and

Title	Years covered	Citation
		Prevention, Cooperative Agreement U58/SO000047-3 and 3U58SO000047-02W1 (2013).
Washington Behavioral Risk Factor Surveillance System	2014	Washington State Department of Health, Center for Health Statistics, Behavioral Risk Factor Surveillance System, supported in part by the Centers for Disease Control and Prevention, Cooperative Agreement U58/SO000047-4 and 3U58SO000047-03W1 (2014).
Washington Behavioral Risk Factor Surveillance System	2015	Washington State Department of Health, Center for Health Statistics, Behavioral Risk Factor Surveillance System, supported in part by the Centers for Disease Control and Prevention, Cooperative Agreement NU58/DP006066-01 (2015).
Washington Behavioral Risk Factor Surveillance System	2016	Washington State Department of Health, Center for Health Statistics, Behavioral Risk Factor Surveillance System, supported in part by the Centers for Disease Control and Prevention, Cooperative Agreement NU58/DP006066-02 (2016).
Washington Behavioral Risk Factor Surveillance System	2017	Washington State Department of Health, Center for Health Statistics, Behavioral Risk Factor Surveillance System, supported in part by the Centers for Disease Control and Prevention, Cooperative Agreement NU58/DP006066-04 (2017).
Washington Behavioral Risk Factor Surveillance System	2018	Washington State Department of Health, Center for Health Statistics, Behavioral Risk Factor Surveillance System, supported in part by the Centers for Disease Control and Prevention, Cooperative Agreement NU58/DP006066-04 (2018).

Title	Years covered	Citation
Washington Behavioral Risk Factor Surveillance System	2019	Washington State Department of Health, Center for Health Statistics, Behavioral Risk Factor Surveillance System, supported in part by the Centers for Disease Control and Prevention, Cooperative Agreement U58/DP006066-05 (2019).
Wisconsin Behavioral Risk Factor Surveillance System	2000–2010, 2012–2019	Centers for Disease Control and Prevention (CDC), Wisconsin Department of Health Services. United States - Wisconsin Behavioral Risk Factor Surveillance System 2000– 2010, 2012–2019. Madison, WI, United States of America: Wisconsin Department of Health Services.
Wyoming Behavioral Risk Factor Surveillance System	2000–2019	Centers for Disease Control and Prevention (CDC), Wyoming Department of Health. United States - Wyoming Behavioral Risk Factor Surveillance System 2000-2019.

Table S6. Summary of measurement types, years, and resolution of BMI data

Source	National Health and Nutrition Examination Survey (NHANES)	Behavioral Risk Factor Surveillance System (BRFSS)	Gallup Daily
Years extracted	1999–2023 (available in two-year waves. 2020–21 wave was disrupted due to the COVID-19 pandemic, so the 2017–20 (pre-pandemic) release was used. The 2021–2023 wave spanned August 2021–August 2023.	1999–2020. 1999 and 2020 data were only used in the self-report correction model.	2008–2017.
Finest geographic resolution	National.	Variously, counties, Core-Based Statistical Areas (including Metropolitan and Micropolitan Statistical Areas), metropolitan divisions, or state.	County.
Interview mode	In-person interview followed by a physical examination.	Telephone interview.	Telephone interview.
Measurement of BMI	Calculated from self-reported and measured height and weight.	Calculated from self-reported height and weight.	Calculated from self-reported height and weight.
Study-specific exclusion criteria applied	Asian, AIAN, and Multiracial from 1999–2002 and 2017–2020, and 2003–2010 for ages 60+, due to instability in data.		Asian, AIAN, and Multiracial, due to inconsistencies is racial categorization compared to NHANES.

Sample size after exclusions	56,981	6,902,216	2,245,371

Table S7. Population mask

Stratum	AIAN			Asian			Black			Latino			White			Total		
	Counties masked	Person-years masked	Percent person-years in stratum	Counties masked	Person-years masked	Percent person-years in stratum	Counties masked	Person-years masked	Percent person-years in stratum	Counties masked	Person-years masked	Percent person-years in stratum	Counties masked	Person-years masked	Percent person-years in stratum	Counties masked	Person-years masked	Percent person-years in stratum
**Total**	2639 (84.9%)	9.1* (17.8%)		2441 (78.5%)	8.5* (2.6%)		1622 (52.2%)	7.5* (1.0%)		1634 (52.5%)	11.4* (1.2%)		60 (1.9%)	0.8* (0.0%)		31 (1.0%)	0.4* (0.0%)	
**Urban or rural code**																		
Large central metro	8 (10.1%)	0.1 (0.6%)	17.0%	0 (0.0%)	0.0 (0.0%)	52.2%	0 (0.0%)	0.0 (0.0%)	42.4%	0 (0.0%)	0.0 (0.0%)	49.5%	0 (0.0%)	0.0 (0.0%)	22.5%	0 (0.0%)	0.0 (0.0%)	30.9%
Large fringe metro	309 (78.0%)	1.6 (28.7%)	11.0%	184 (46.5%)	0.9 (1.2%)	25.0%	83 (21.0%)	0.6 (0.3%)	22.0%	104 (26.3%)	0.9 (0.5%)	17.8%	0 (0.0%)	0.0 (0.0%)	25.7%	0 (0.0%)	0.0 (0.0%)	23.8%
Medium metro	361 (74.0%)	1.5 (15.2%)	19.5%	239 (49.0%)	0.9 (1.7%)	15.6%	115 (23.6%)	0.6 (0.4%)	18.0%	121 (24.8%)	0.8 (0.4%)	19.7%	1 (0.2%)	0.0 (0.0%)	21.6%	0 (0.0%)	0.0 (0.0%)	20.5%
Small metro	358 (81.4%)	1.4 (22.2%)	12.4%	240 (54.5%)	1.1 (8.5%)	3.9%	127 (28.9%)	0.6 (1.1%)	7.1%	149 (33.9%)	0.9 (1.6%)	5.9%	2 (0.5%)	0.0 (0.0%)	10.9%	1 (0.2%)	0.0 (0.0%)	9.2%
Micropolitan	717 (87.5%)	2.2 (21.7%)	19.5%	692 (84.5%)	3.6 (40.9%)	2.7%	384 (46.9%)	2.5 (5.2%)	6.0%	306 (37.4%)	2.4 (5.1%)	4.8%	9 (1.1%)	0.1 (0.0%)	11.3%	6 (0.7%)	0.1 (0.0%)	9.2%
Noncore	1695 (92.8%)	2.4 (22.5%)	20.6%	1805 (98.8%)	2.0 (91.7%)	0.7%	1339 (73.3%)	3.3 (9.5%)	4.4%	1399 (76.6%)	6.4 (28.4%)	2.3%	60 (3.3%)	0.7 (0.2%)	8.1%	31 (1.7%)	0.4 (0.1%)	6.4%
**Census region**																		
South	1247 (88.6%)	3.9 (23.7%)	32.2%	1134 (80.6%)	4.0 (5.6%)	21.8%	449 (31.9%)	2.8 (0.6%)	56.3%	688 (48.9%)	5.5 (1.5%)	36.1%	20 (1.4%)	0.2 (0.0%)	34.9%	8 (0.6%)	0.1 (0.0%)	37.1%
West	269 (62.4%)	1.3 (5.6%)	44.3%	310 (71.9%)	1.2 (0.8%)	46.6%	320 (74.2%)	1.2 (1.7%)	9.2%	151 (35.0%)	0.9 (0.2%)	40.8%	18 (4.2%)	0.3 (0.0%)	19.6%	9 (2.1%)	0.1 (0.0%)	23.3%
Northeast	173 (79.7%)	1.0 (30.9%)	6.0%	110 (50.7%)	0.8 (1.3%)	19.9%	69 (31.8%)	0.6 (0.4%)	16.3%	67 (30.9%)	0.7 (0.5%)	14.0%	0 (0.0%)	0.0 (0.0%)	19.3%	0 (0.0%)	0.0 (0.0%)	18.0%
Midwest	950 (90.0%)	3.0 (33.7%)	17.5%	887 (84.1%)	2.5 (6.4%)	11.8%	784 (74.3%)	3.0 (2.1%)	18.2%	728 (69.0%)	4.4 (4.9%)	9.1%	22 (2.1%)	0.3 (0.0%)	26.2%	14 (1.3%)	0.2 (0.0%)	21.6%

*Person-years are given in millions.

Modelled prevalence and attributable YLL estimates were masked (not displayed) in all years for county and racial and/or ethnic populations with a mean annual population of fewer than 1000 people from 2000 to 2019. The number of unique counties masked (and corresponding percentage of all counties), the person-years in millions represented by these county-years (and corresponding percentage of all person-years), and the percentage of the person-years masked in each stratum are listed in this table. AIAN = American Indian or Alaska Native.

Table S8. Ensemble distribution weights

Sex	Age range	Racial and/or ethnic population	Gamma	Inverse-Gamma	Log-Logistic	Gumbel	Inverse-Weibull	Log-Normal	Beta†	Mirrored Gamma	Mirrored Gumbel	Weibull
Female	20 - 69	AIAN*	-	0.91	-	-	-	-	0.09	-	-	-
		Asian	-	-	-	0.67	0.33	-	-	-	-	-
		Black	-	-	-	0.65	-	-	0.32	-	0.04	-
		Latino	-	0.38	-	0.4	-	-	0.18	0.04	-	-
		White	-	-	-	1	-	-	-	-	-	-
	70+	AIAN	-	-	0.87	-	-	-	0.04	0.09	-	-
		Asian	-	-	0.05	0.7	0.24	-	-	-	-	-
		Black	-	0.2	0.16	-	-	-	0.64	-	-	-
		Latino	-	0.5	-	-	0.26	-	-	-	-	0.23
		White	-	-	0.03	-	0.53	-	0.37	0.06	-	-
Male	20 - 69	AIAN	-	0.96	-	-	-	0.04	-	-	-	-
		Asian	-	0.08	0.44	-	0.47	-	-	-	-	-
		Black	-	-	-	0.81	0.05	-	0.15	-	-	-
		Latino	-	0.2	-	-	0.52	-	-	-	0	0.28
		White	-	-	0.32	0.62	-	-	0.06	-	-	-
	70+	AIAN	-	-	-	-	-	-	-	0.53	0.47	-
		Asian	-	-	0.07	-	0.51	-	-	-	0.42	-
		Black	0.27	-	-	0.06	-	0.4	0.27	-	-	-
		Latino	-	0.25	-	-	0.48	-	-	-	-	0.27
		White	-	0	-	-	0.64	-	0	-	0.18	0.19

* AIAN = American Indian and Alaska Native

† Beta distribution is scaled and shifted over the support 10–50.

Normal distribution was candidate weight but received no weight in any population.

Supplementary Figures

Figure S1. Data and modelling flowchart

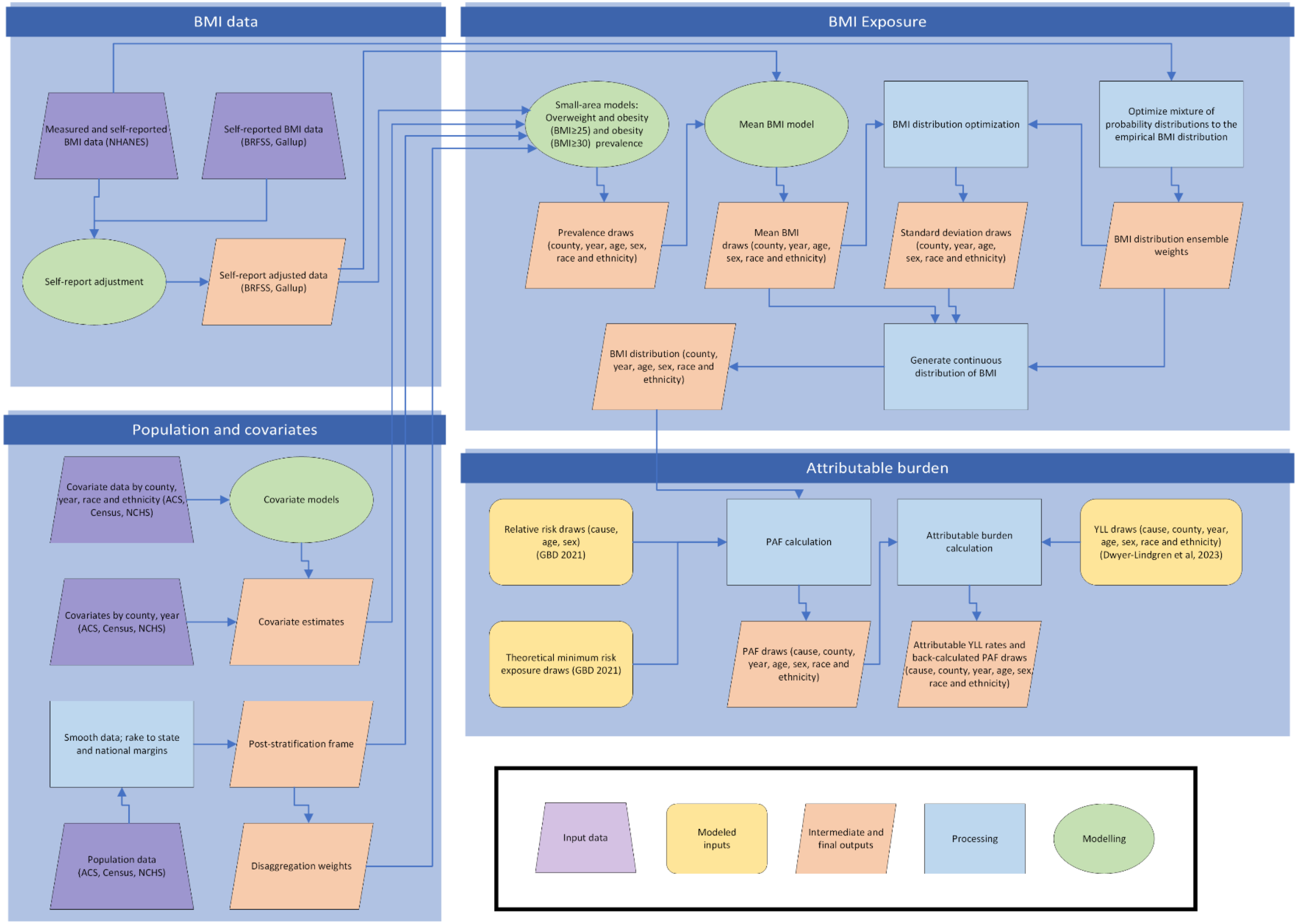

Figure S2. Age-standardised prevalence of obesity (BMI ≥ 30), 2019, male

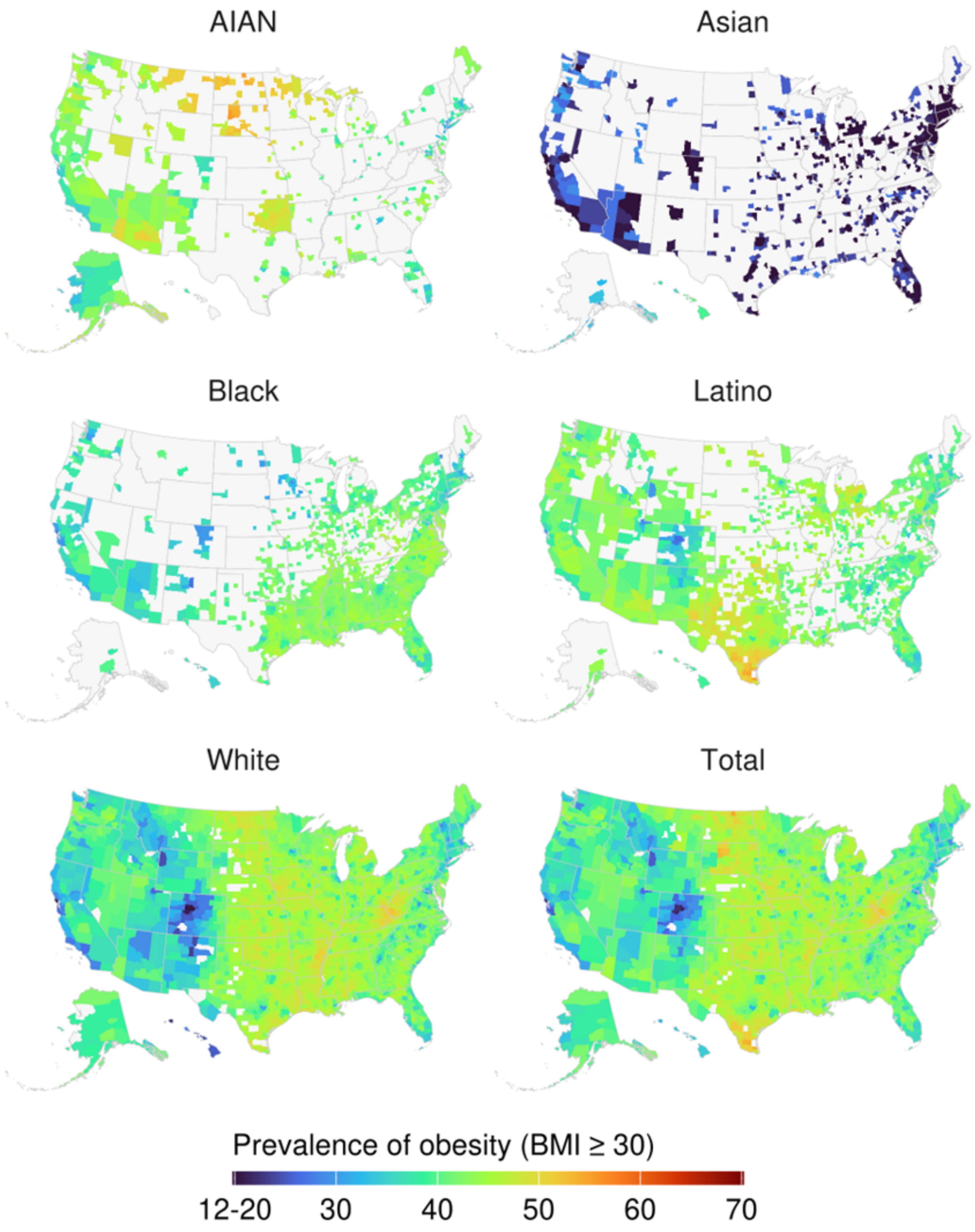

Note: estimates have been masked for county and racial and ethnic populations with a mean annual population fewer than 1000 people.

Figure S3. Age-standardised prevalence of obesity (BMI ≥ 30), 2019, female

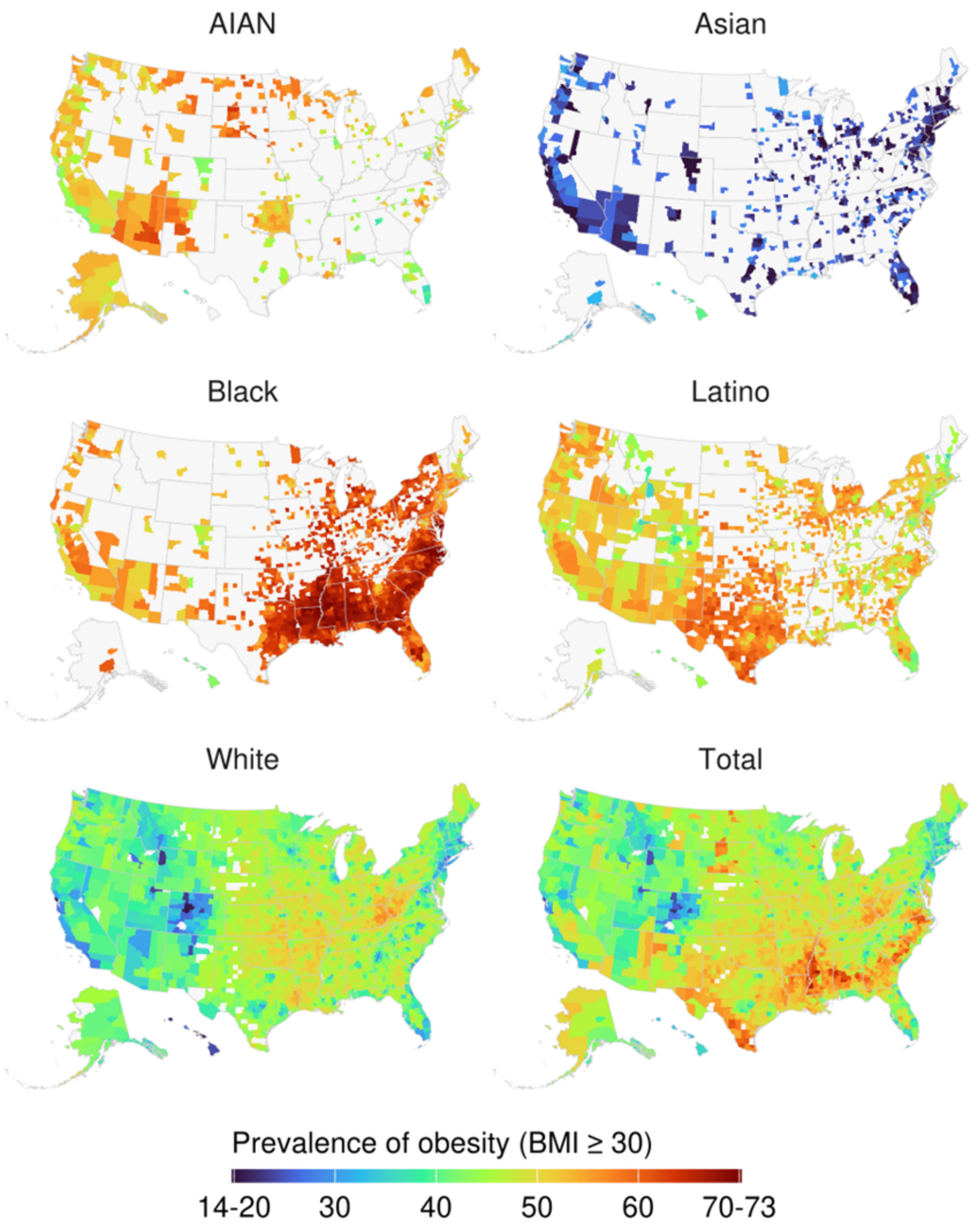

Note: estimates have been masked for county and racial and ethnic populations with a mean annual population fewer than 1000 people.

Figure S4. Age-standardised attributable YLL rates and PAFs, all causes, 2000–2019

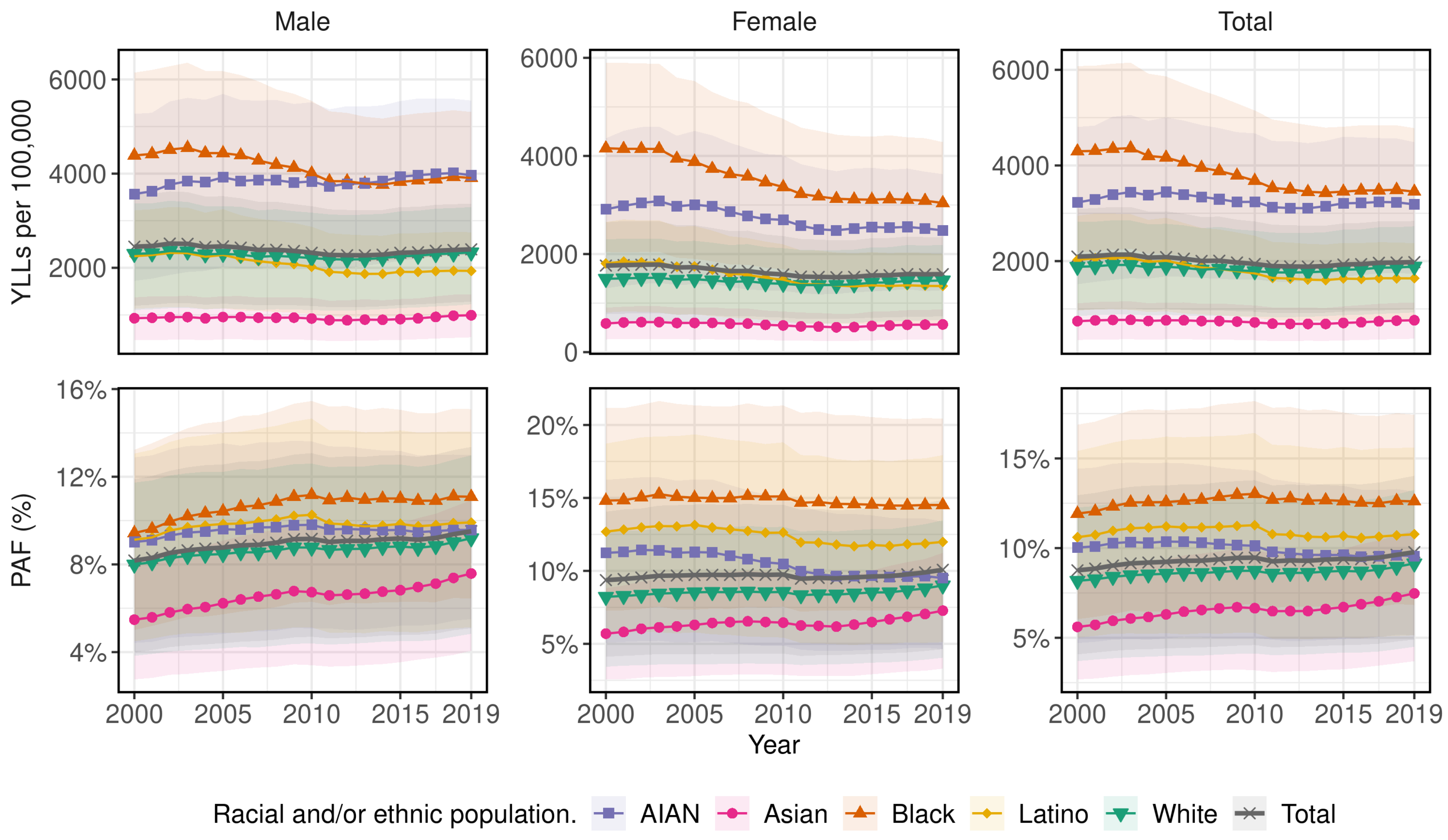

Note: shaded areas indicate the 95% confidence interval

Figure S5. Age-standardised attributable YLL rates and PAFs, non-communicable, 2000–2019

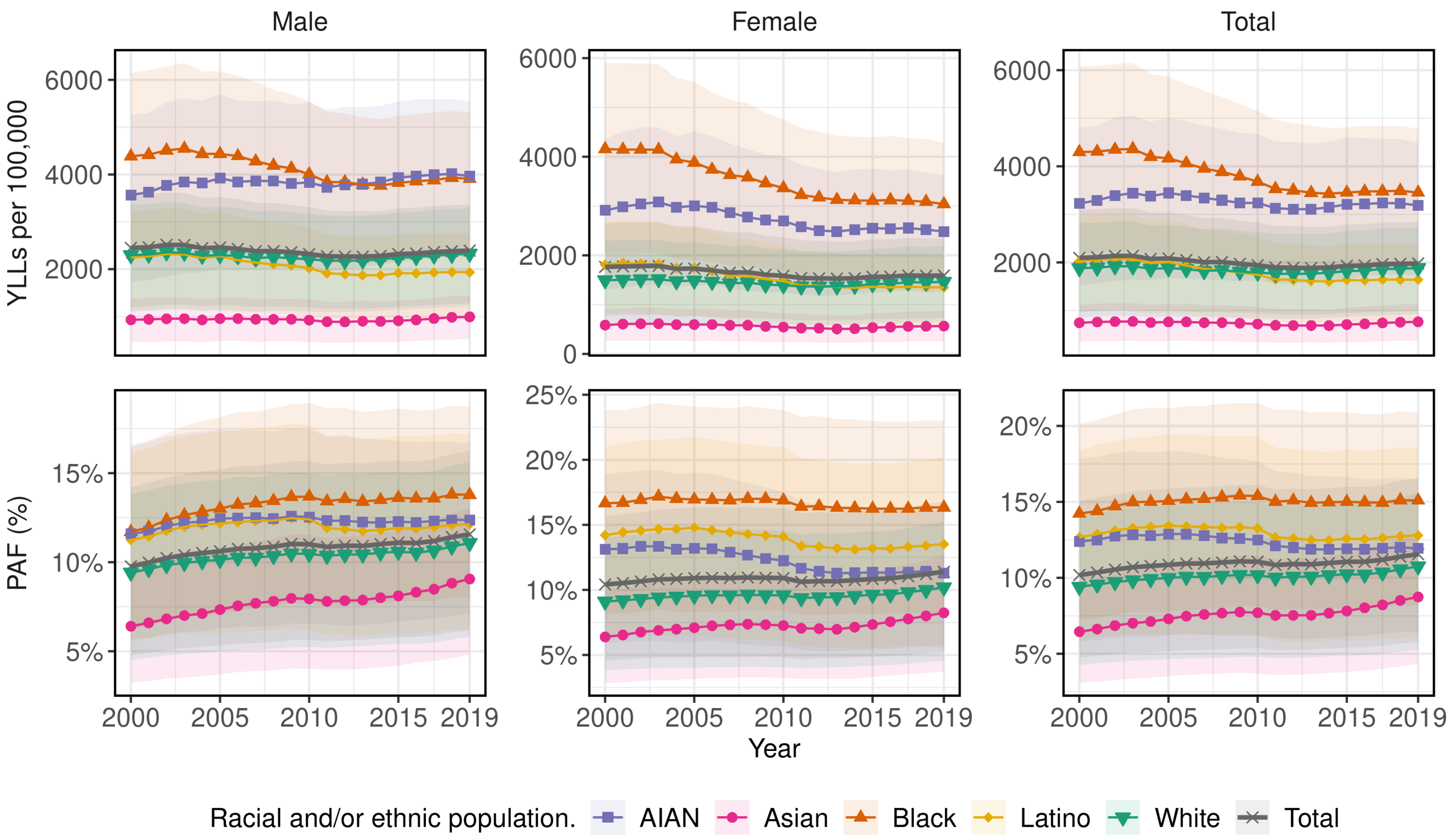

Note: shaded areas indicate the 95% confidence interval

Figure S6. Age-standardised attributable YLL rates and PAFs, neoplasms, 2000–2019

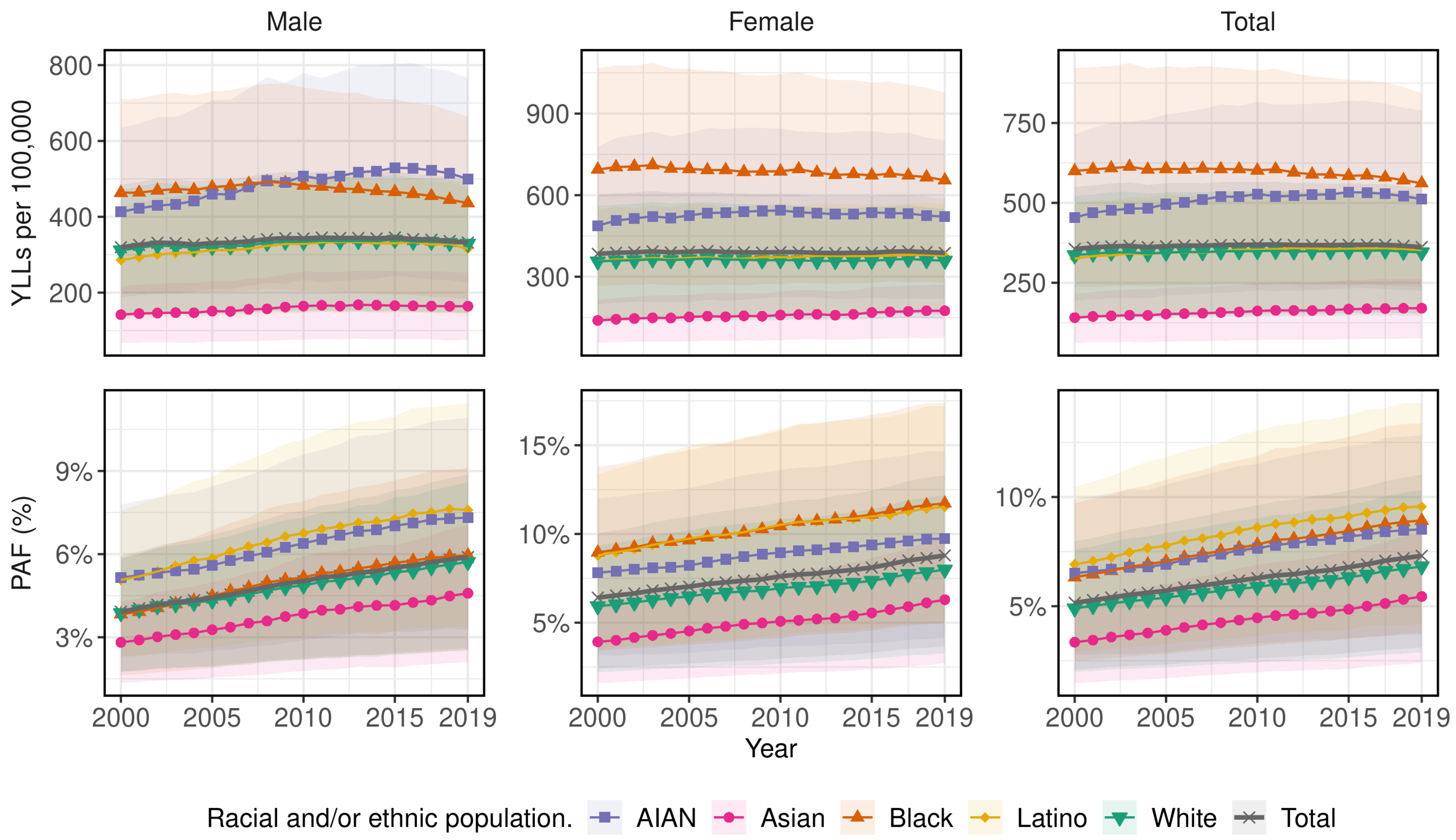

Note: shaded areas indicate the 95% confidence interval

Figure S7. Age-standardised attributable YLL rates and PAFs, colorectal cancer, 2000–2019

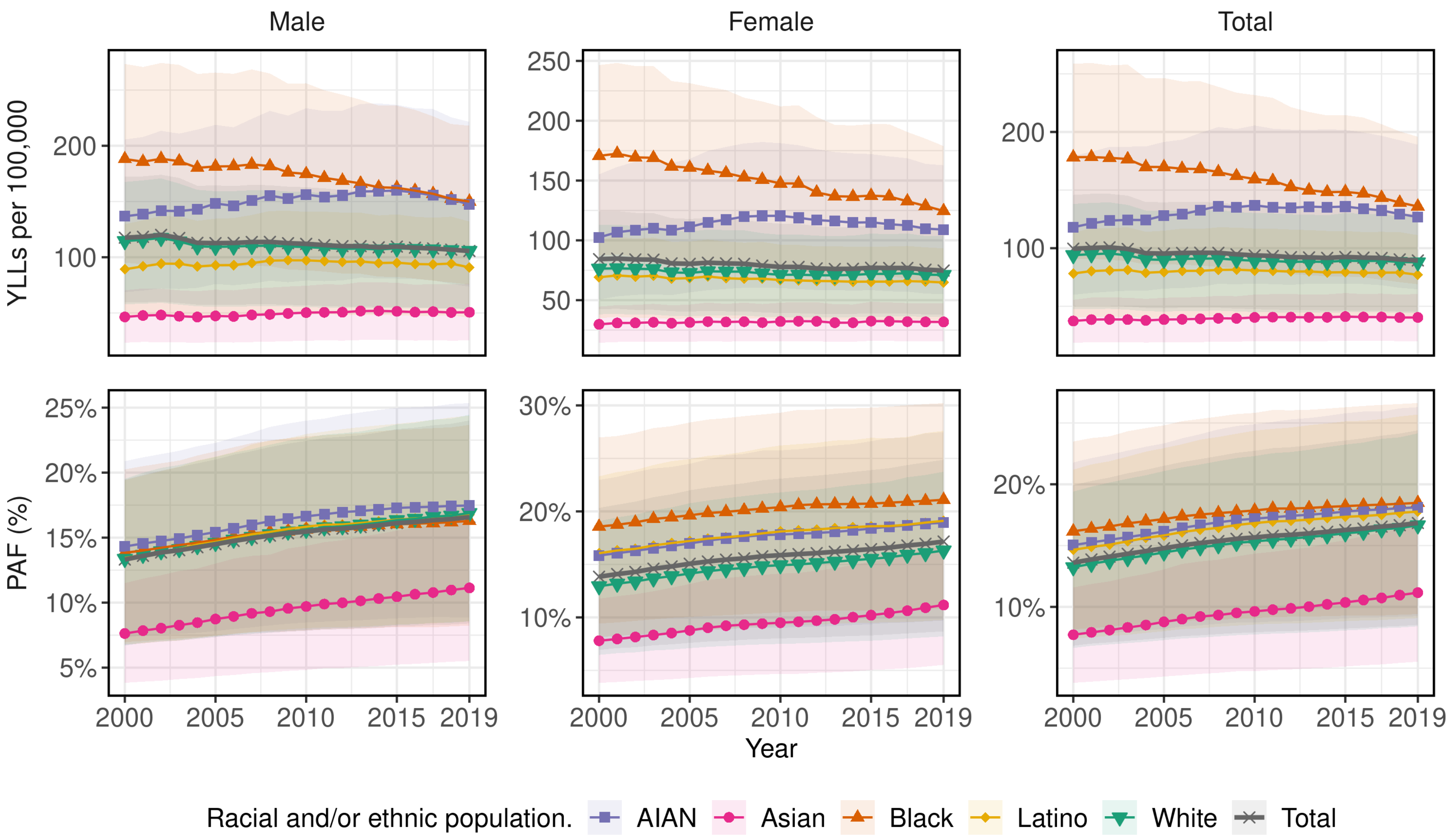

Note: shaded areas indicate the 95% confidence interval

Figure S8. Age-standardised attributable YLL rates and PAFs, liver cancer, 2000–2019

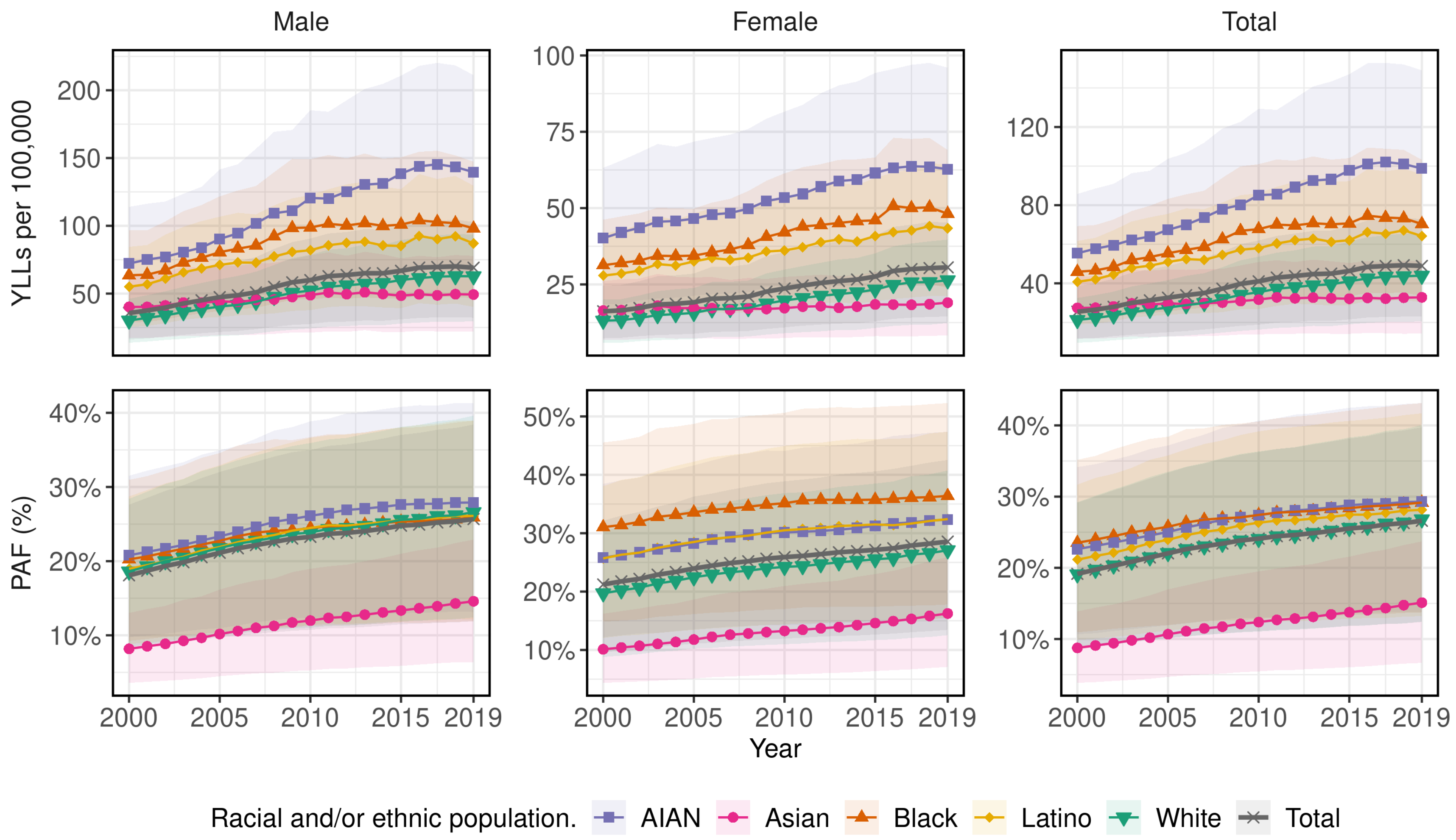

Note: shaded areas indicate the 95% confidence interval

Figure S9. Age-standardised attributable YLL rates and PAFs, gallbladder cancer, 2000–2019

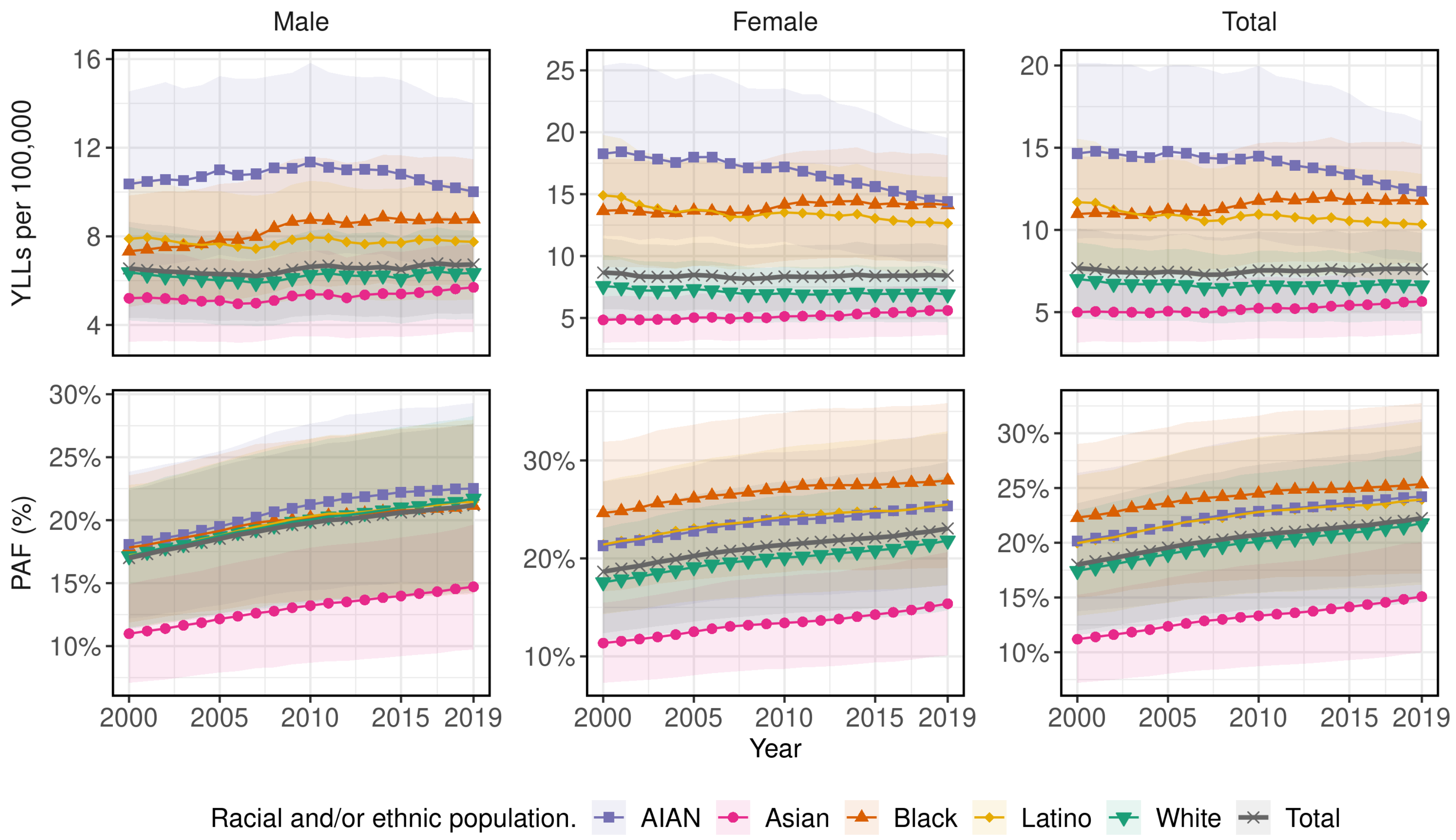

Note: shaded areas indicate the 95% confidence interval

Figure S10. Age-standardised attributable YLL rates and PAFs, pancreatic cancer, 2000–2019

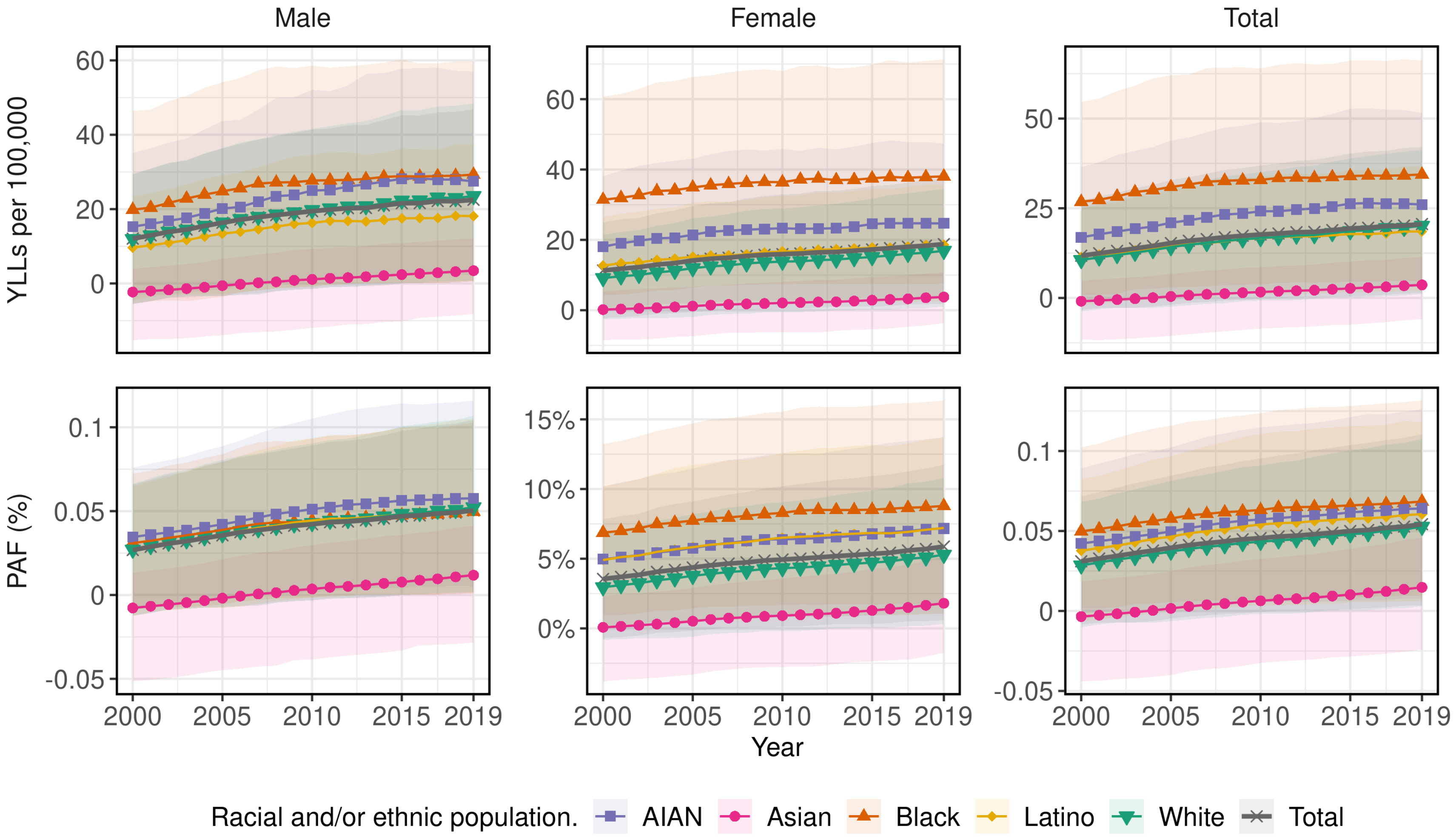

Note: shaded areas indicate the 95% confidence interval

Figure S11. Age-standardised attributable YLL rates and PAFs, breast cancer, 2000–2019

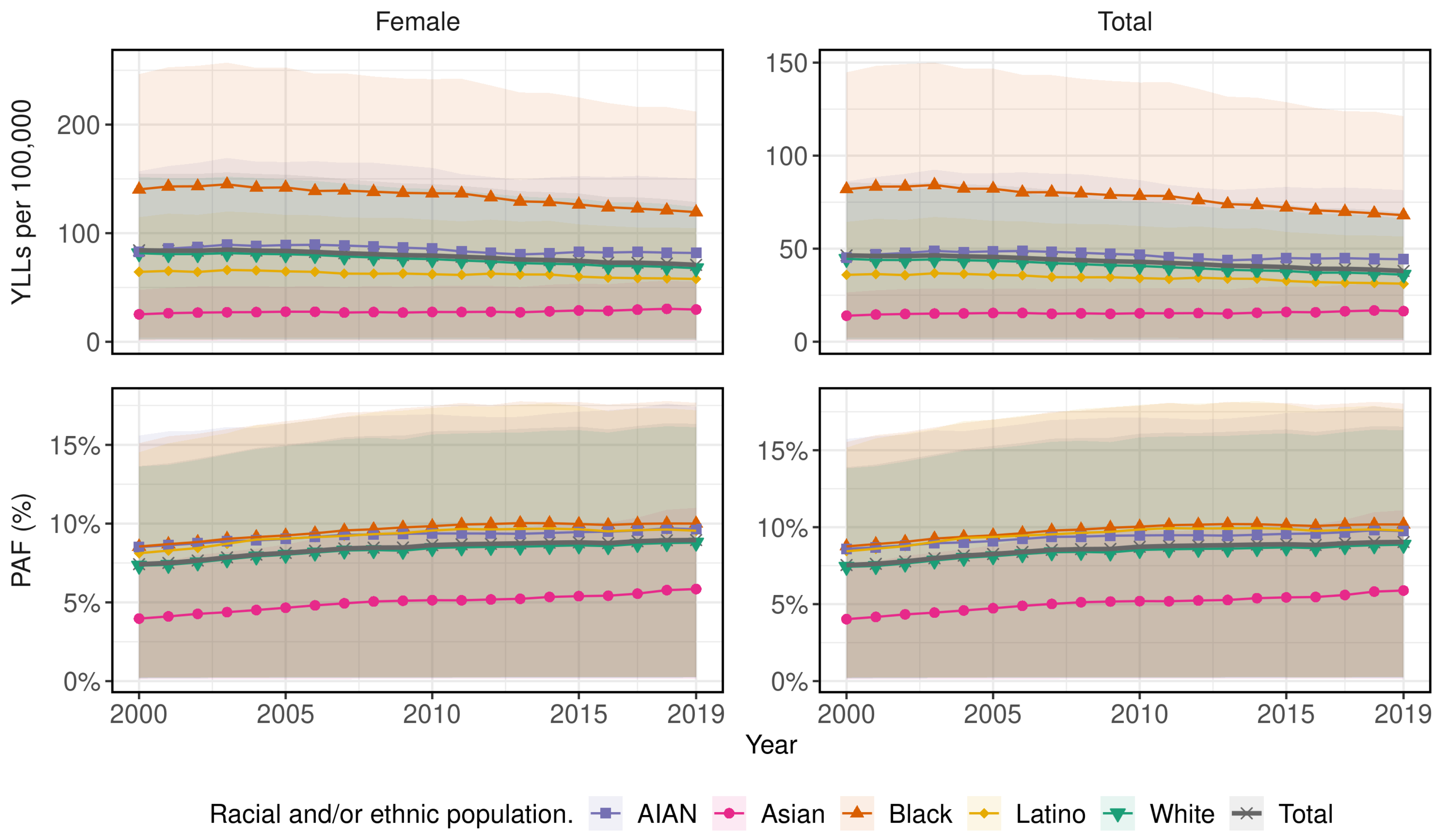

Note: shaded areas indicate the 95% confidence interval

Figure S12. Age-standardised attributable YLL rates and PAFs, uterine cancer, 2000–2019

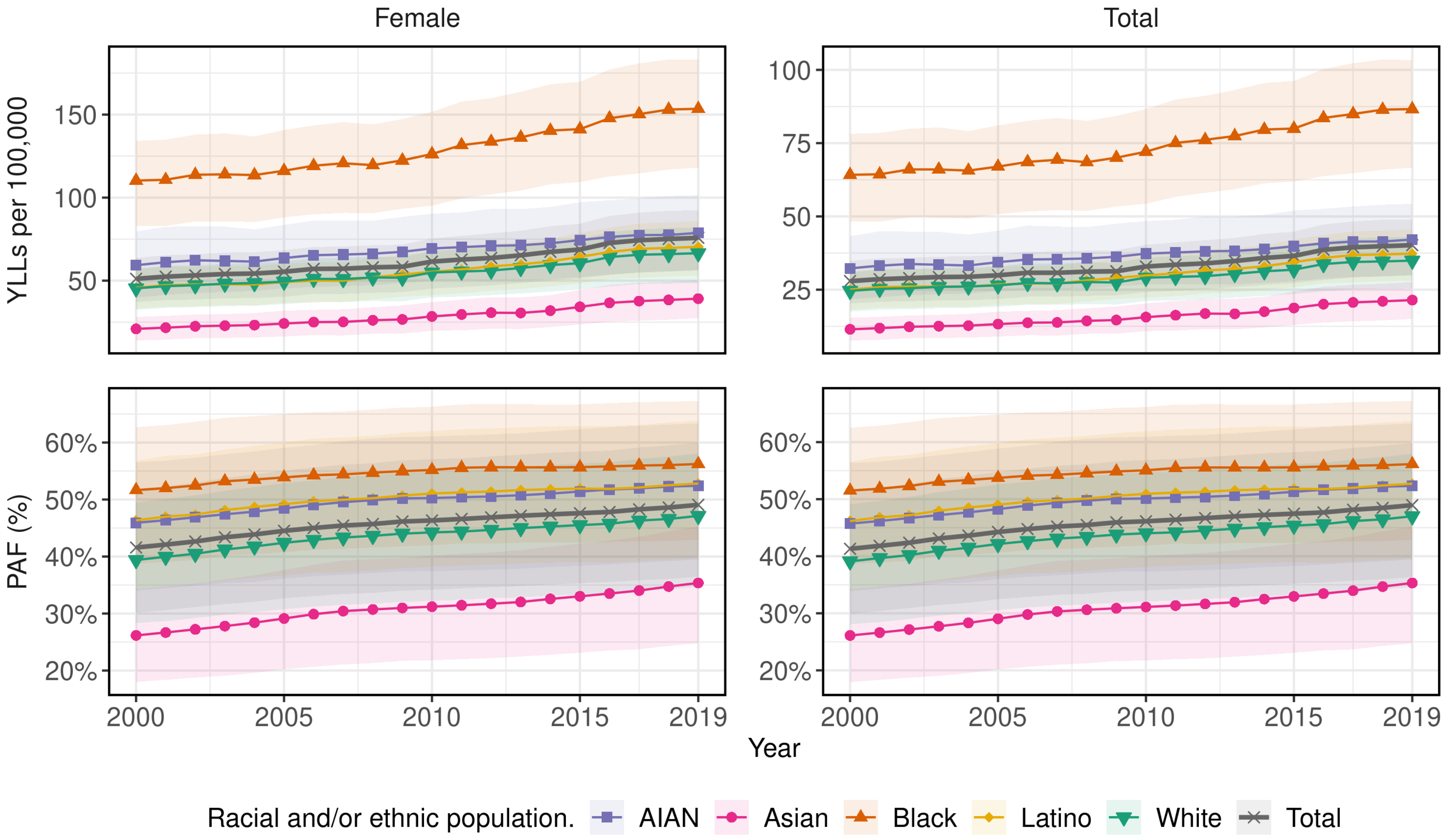

Note: shaded areas indicate the 95% confidence interval

Figure S13. Age-standardised attributable YLL rates and PAFs, ovarian cancer, 2000–2019

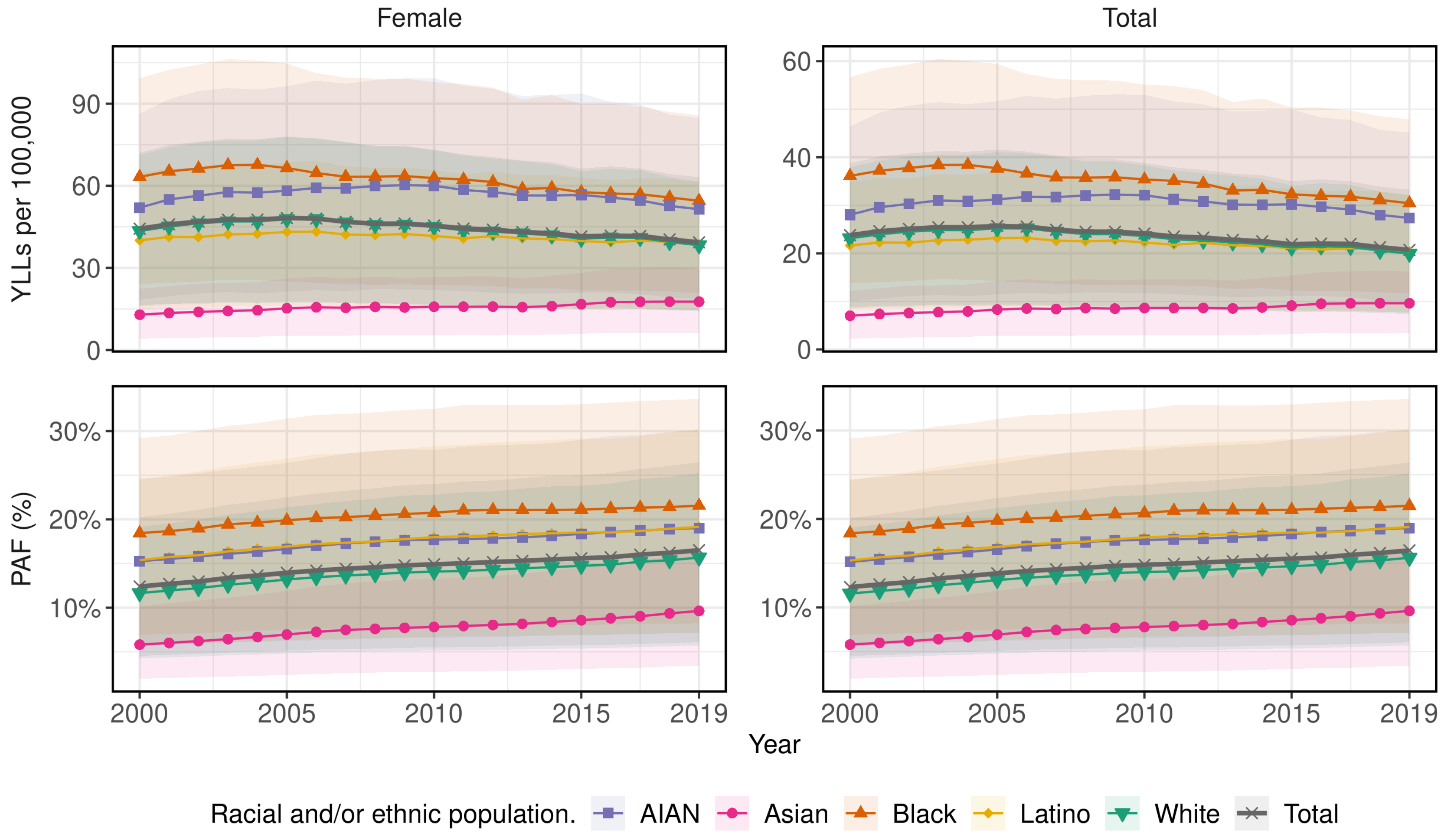

Note: shaded areas indicate the 95% confidence interval

Figure S14. Age-standardised attributable YLL rates and PAFs, kidney cancer, 2000–2019

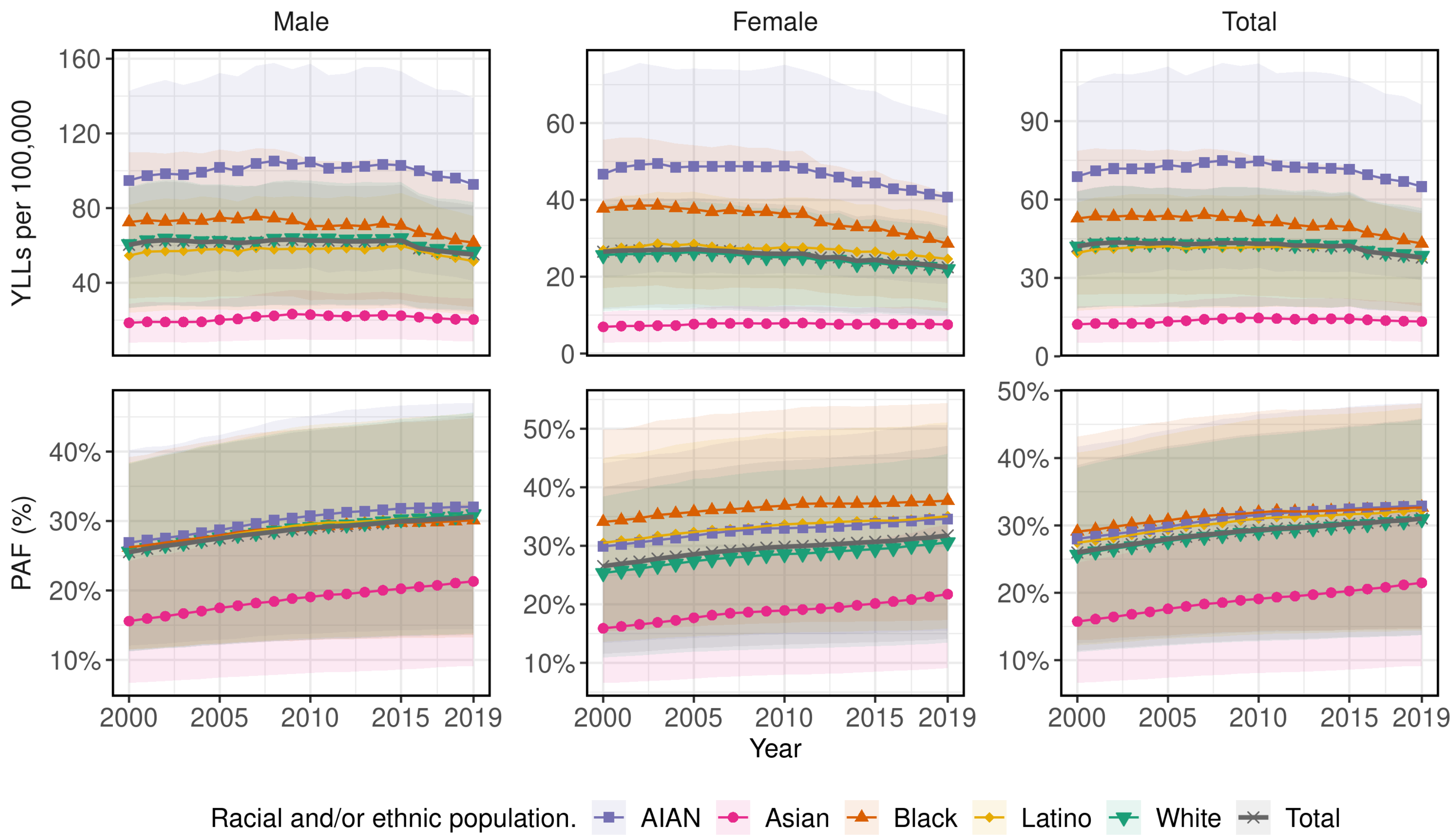

Note: shaded areas indicate the 95% confidence interval

Figure S15. Age-standardised attributable YLL rates and PAFs, thyroid cancer, 2000–2019

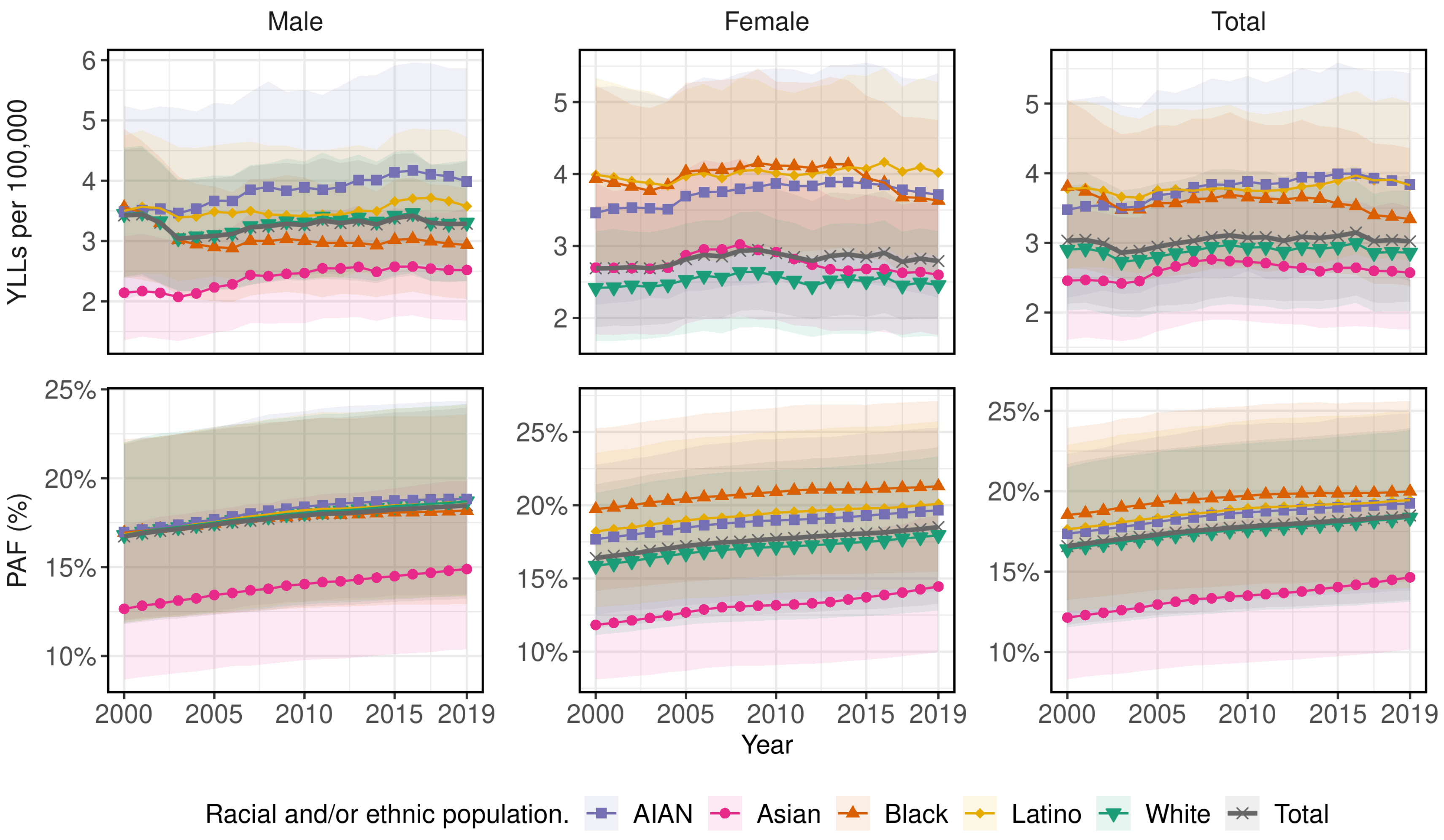

Note: shaded areas indicate the 95% confidence interval

Figure S16. Age-standardised attributable YLL rates and PAFs, lymphoma, 2000–2019

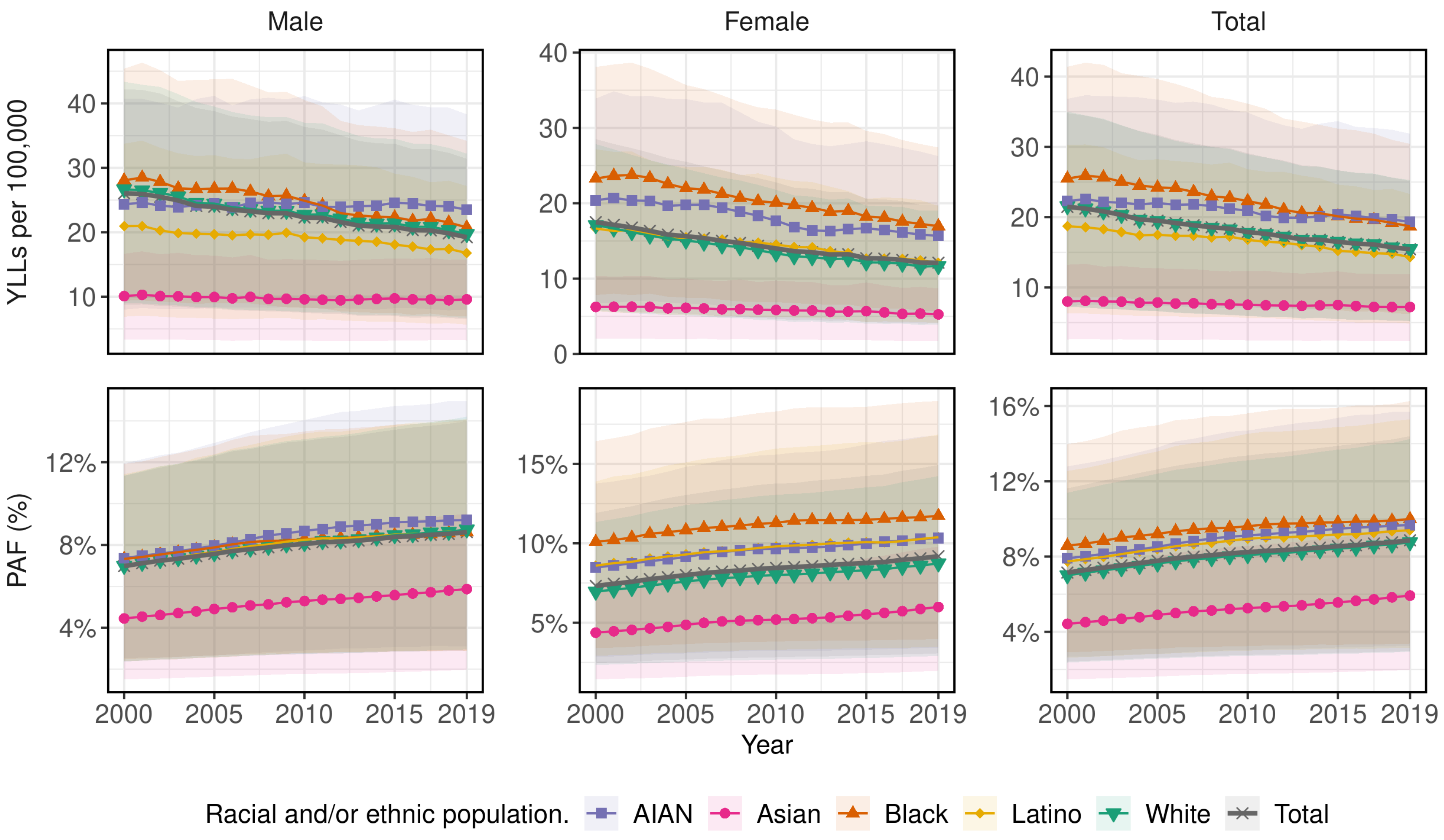

Note: shaded areas indicate the 95% confidence interval

Figure S17. Age-standardised attributable YLL rates and PAFs, Burkitt lymphoma, 2000–2019

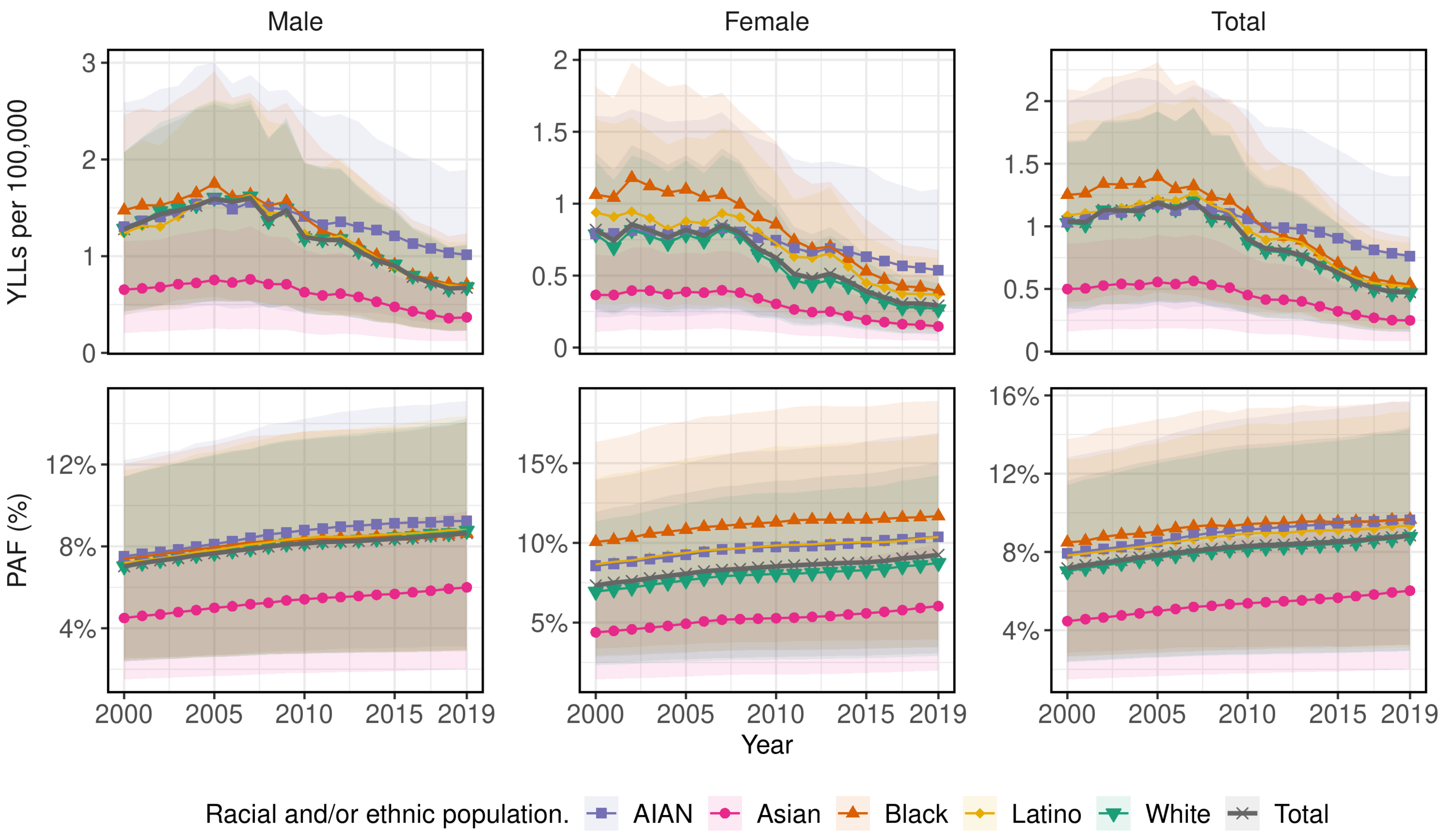

Note: shaded areas indicate the 95% confidence interval

Figure S18. Age-standardised attributable YLL rates and PAFs, other lymphoma, 2000–2019

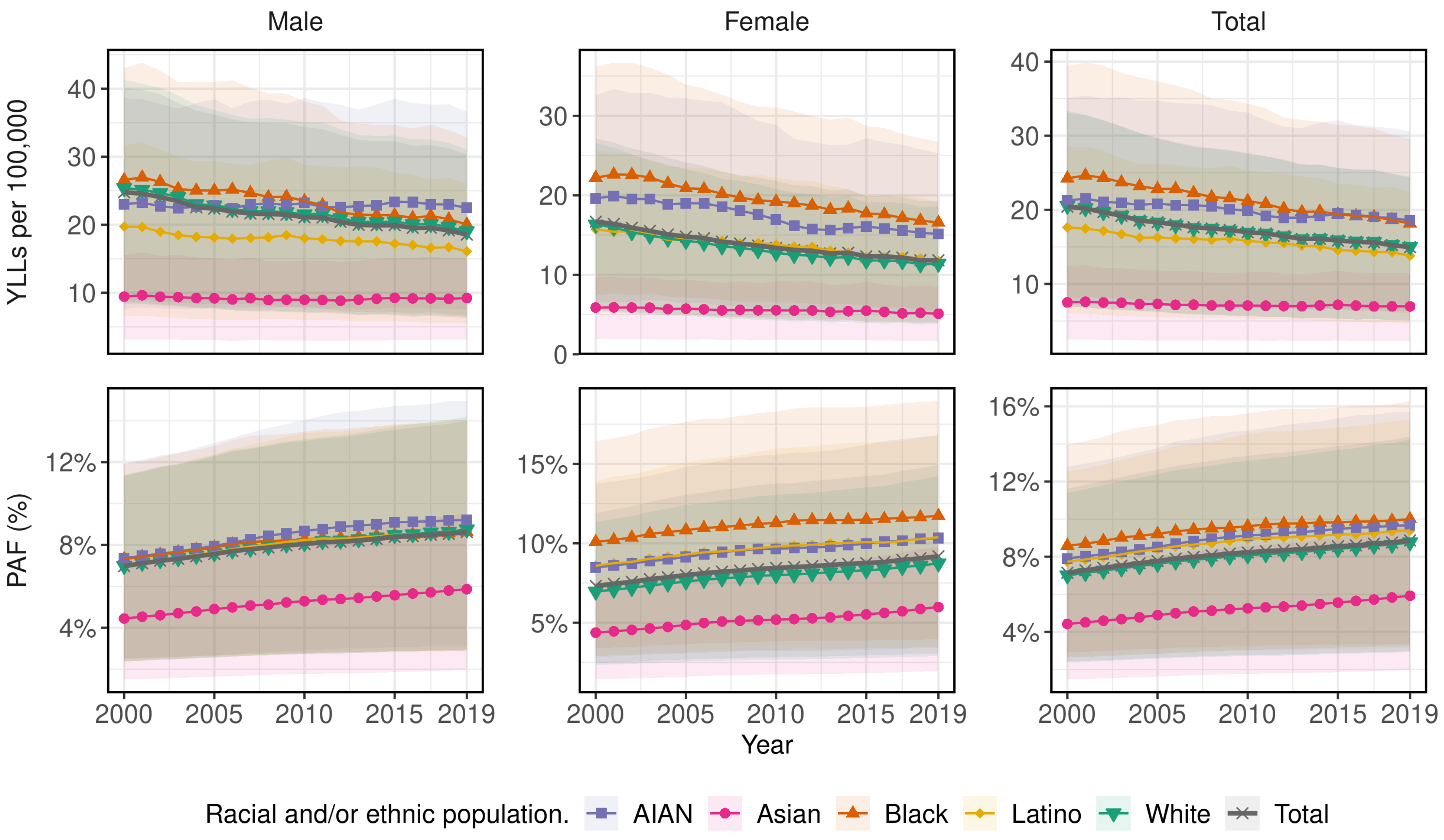

Note: shaded areas indicate the 95% confidence interval

Figure S19. Age-standardised attributable YLL rates and PAFs, myeloma, 2000–2019

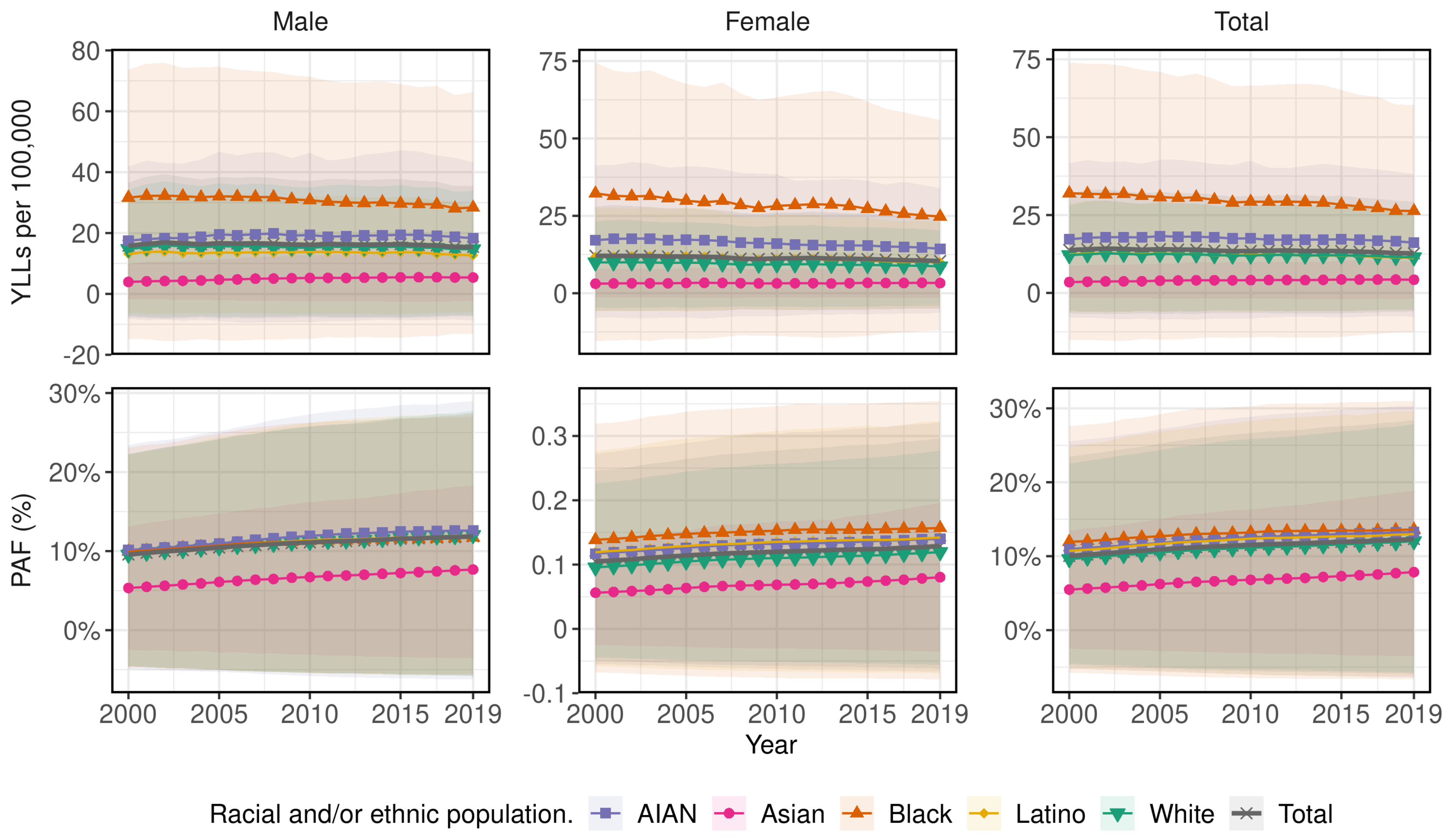

Note: shaded areas indicate the 95% confidence interval

Figure S20. Age-standardised attributable YLL rates and PAFs, leukemia, 2000–2019

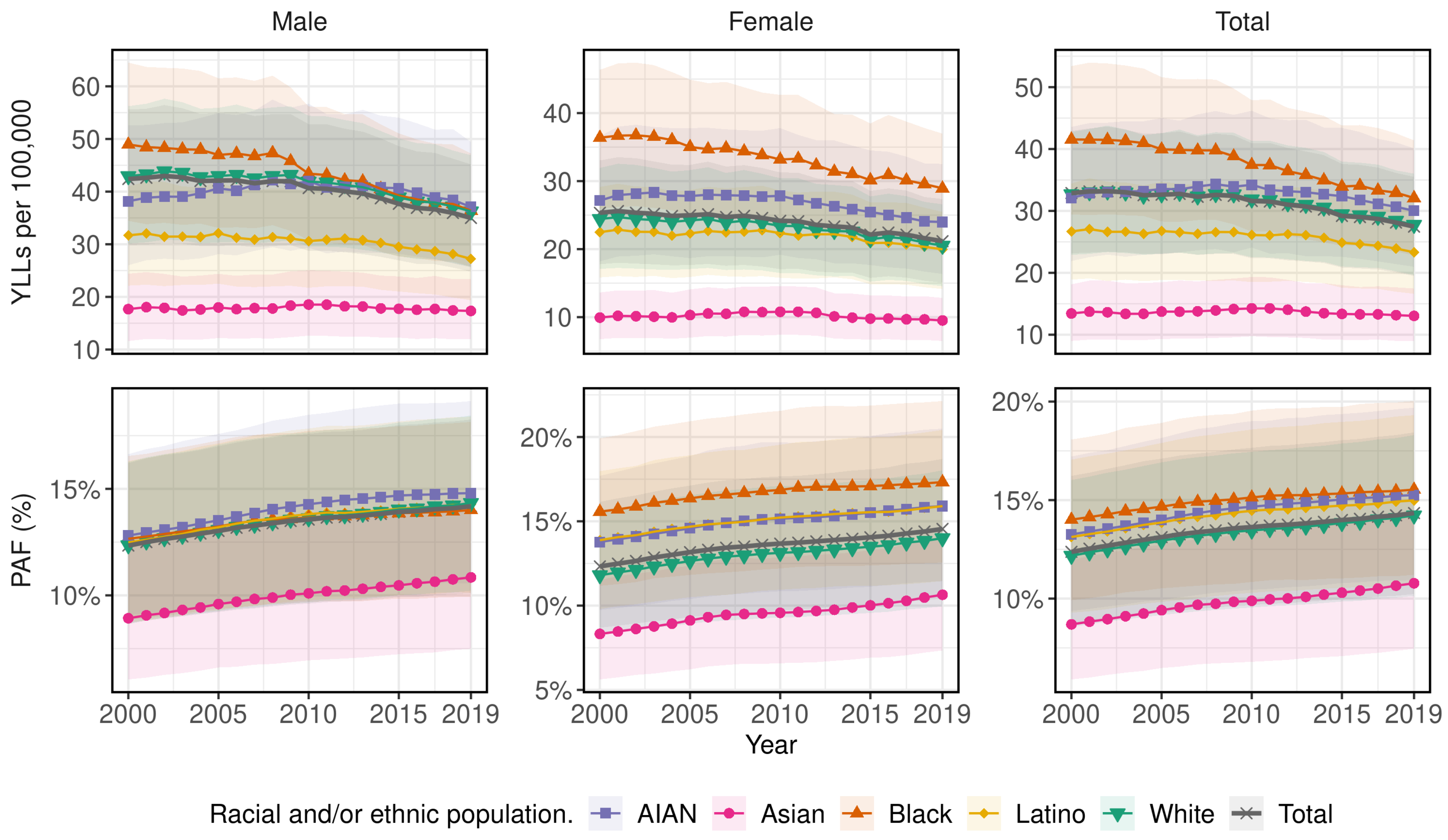

Note: shaded areas indicate the 95% confidence interval

Figure S21. Age-standardised attributable YLL rates and PAFs, acute lymphoid leukemia, 2000–2019

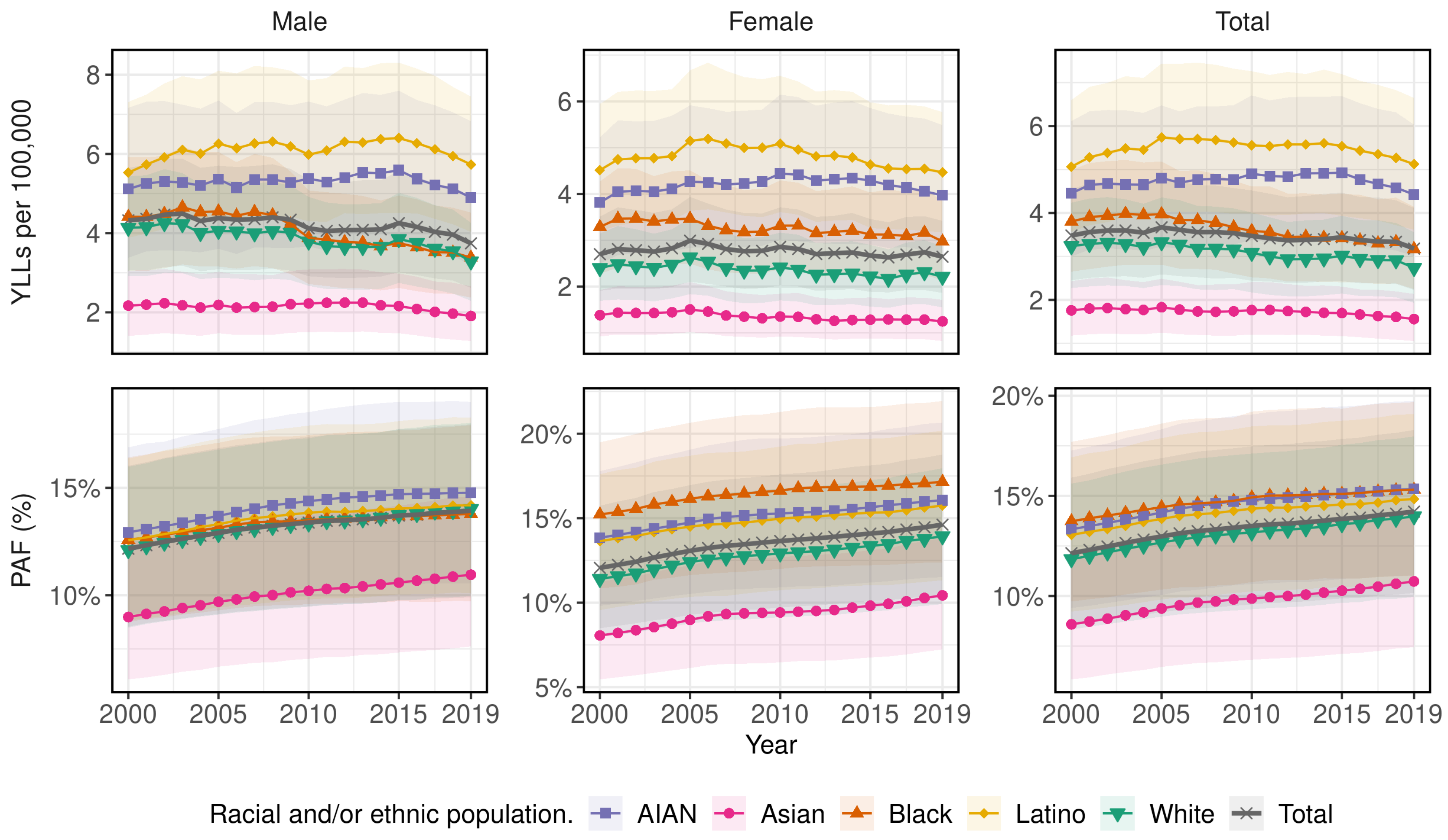

Note: shaded areas indicate the 95% confidence interval

Figure S22. Age-standardised attributable YLL rates and PAFs, chronic lymphoid leukemia, 2000–2019

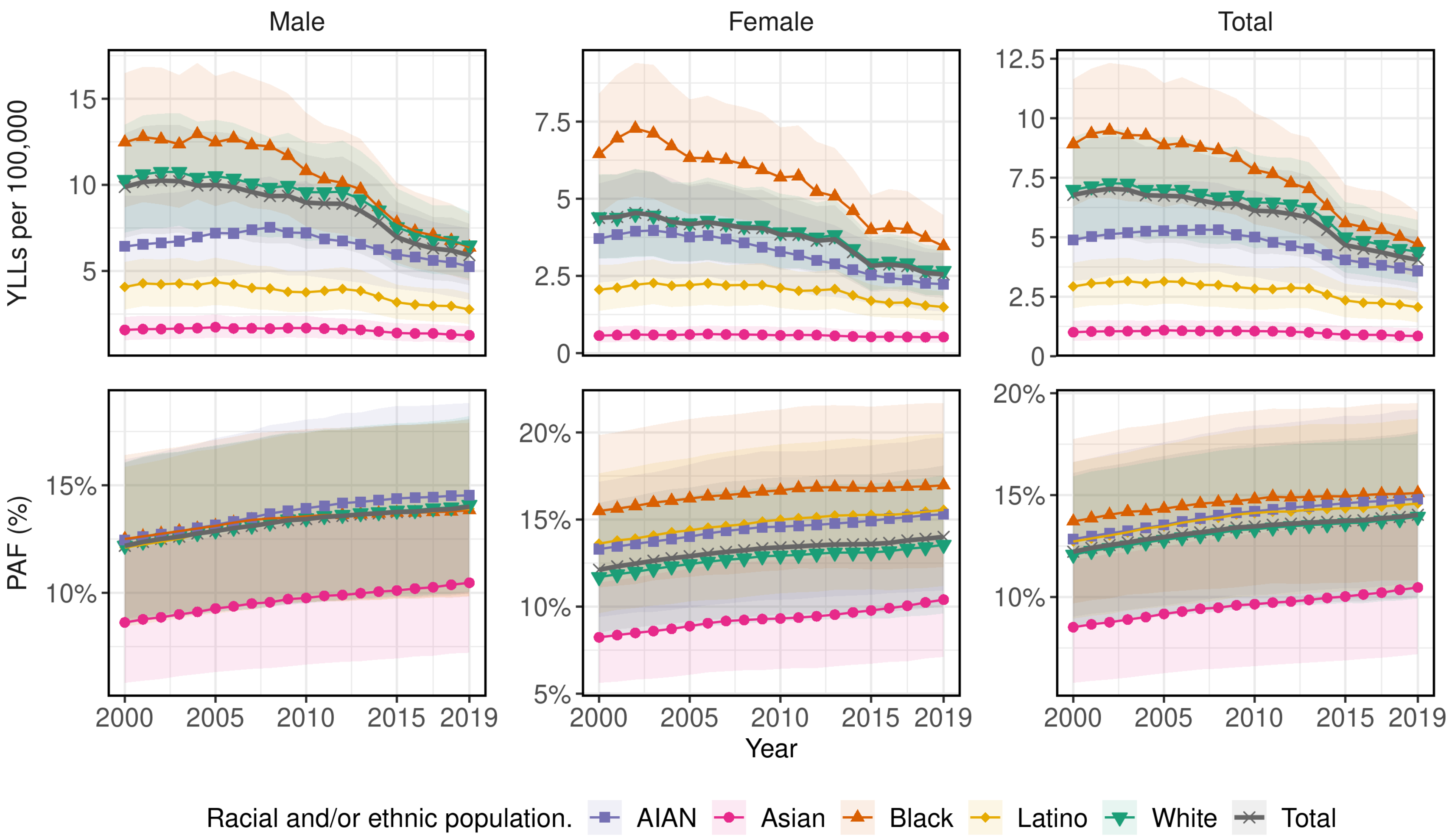

Note: shaded areas indicate the 95% confidence interval

Figure S23. Age-standardised attributable YLL rates and PAFs, acute myeloid leukemia, 2000–2019

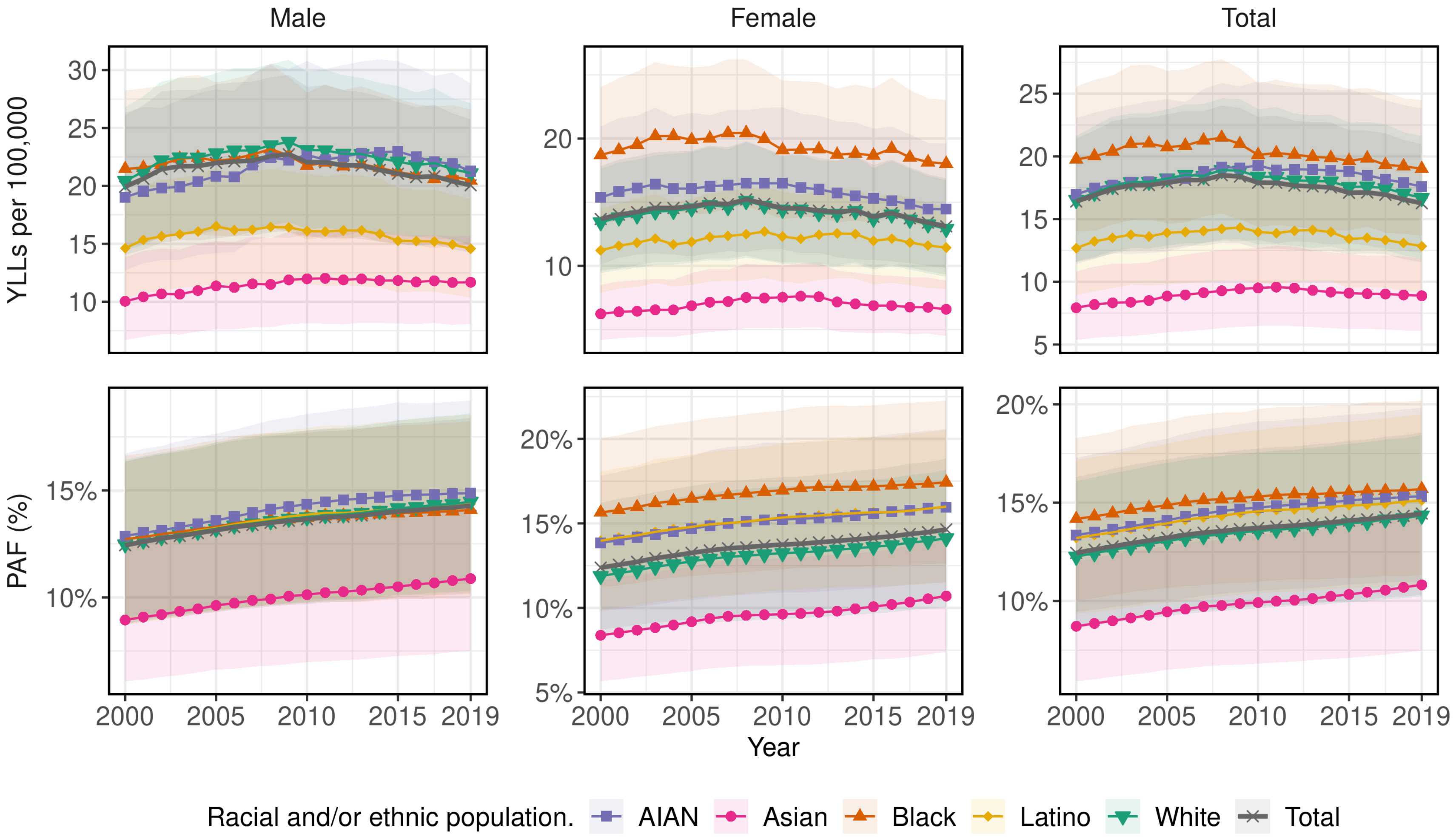

Note: shaded areas indicate the 95% confidence interval

Figure S24. Age-standardised attributable YLL rates and PAFs, chronic myeloid leukemia, 2000–2019

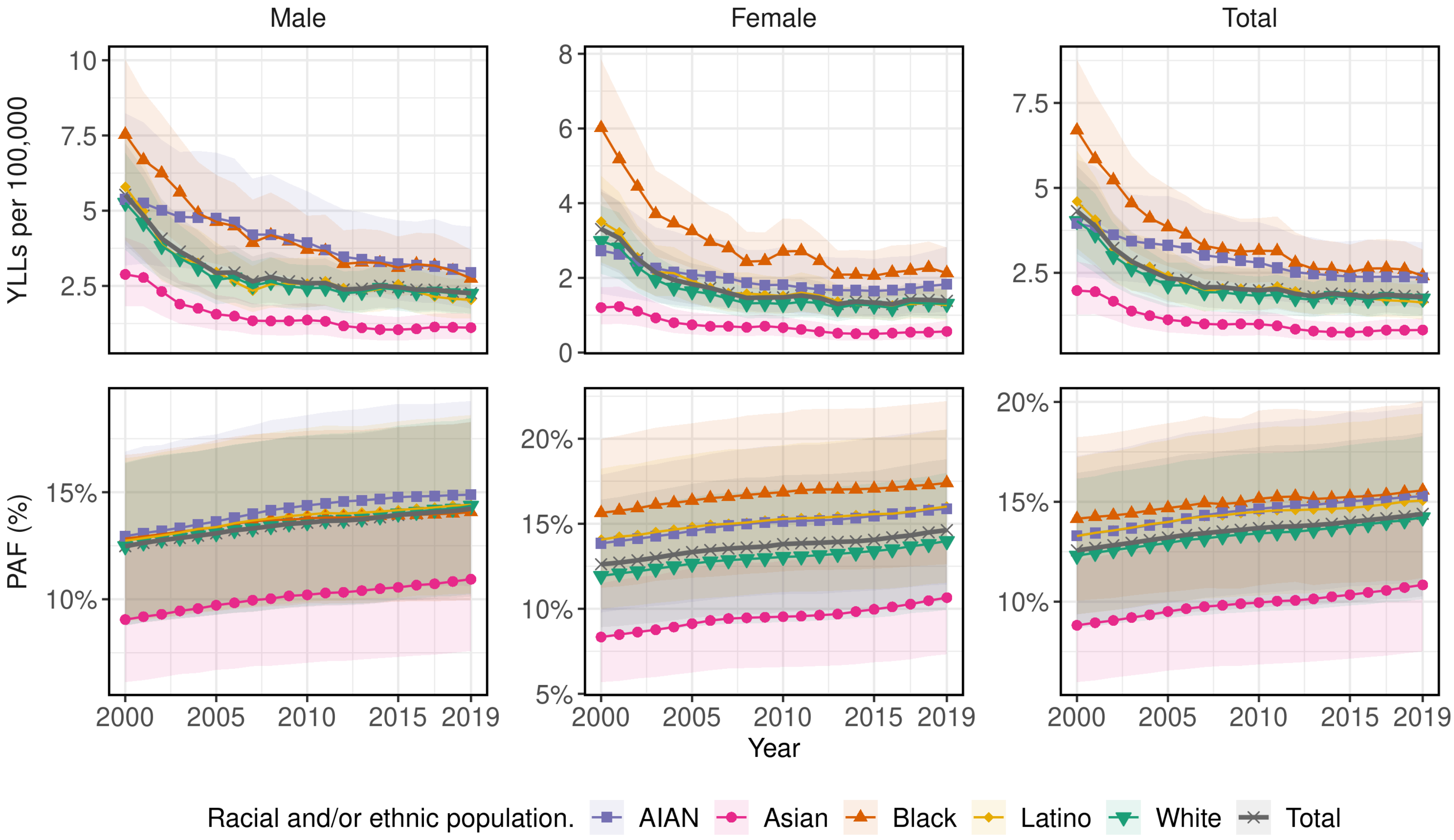

Note: shaded areas indicate the 95% confidence interval

Figure S25. Age-standardised attributable YLL rates and PAFs, other leukemia, 2000–2019

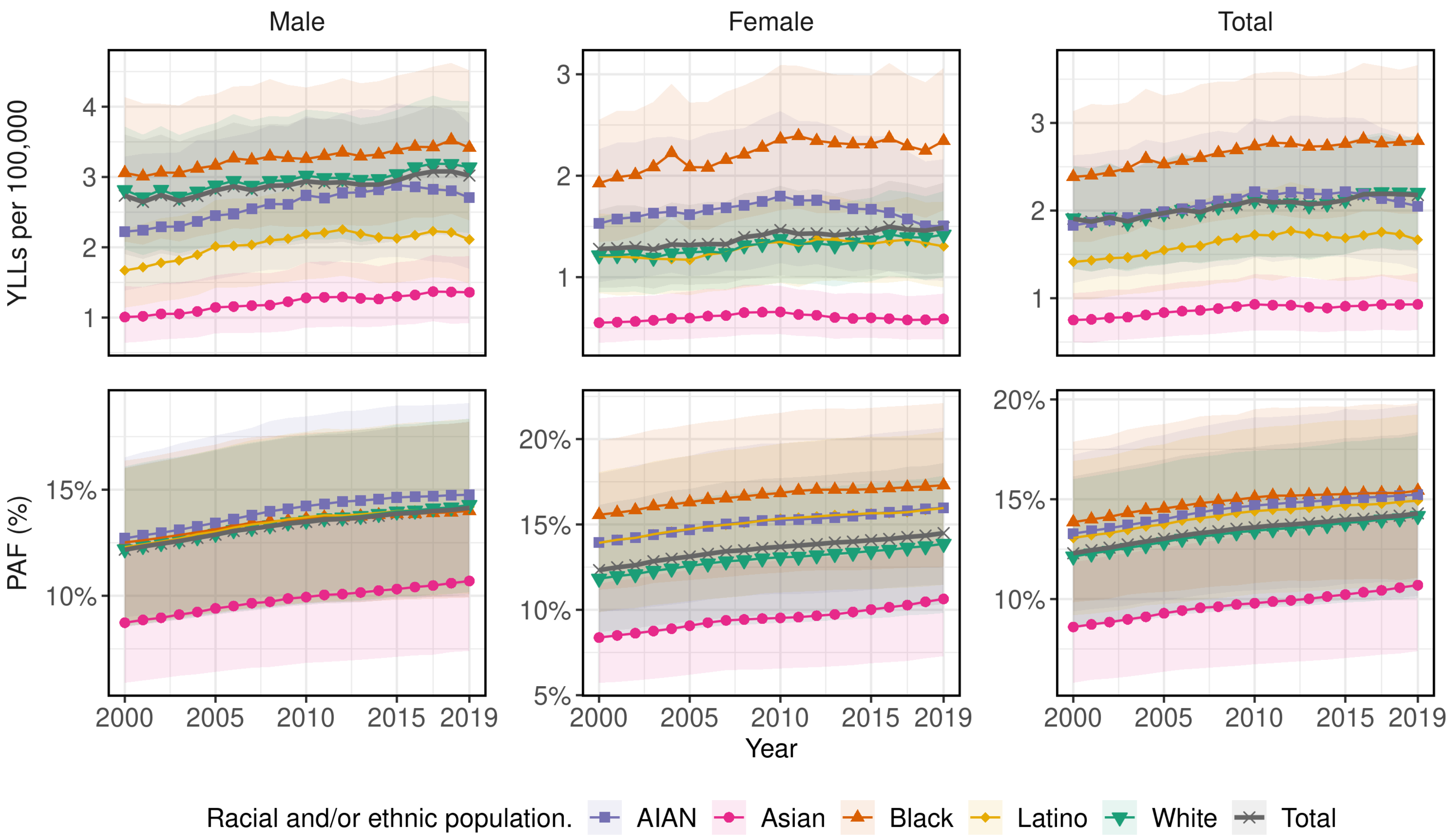

Note: shaded areas indicate the 95% confidence interval

Figure S26. Age-standardised attributable YLL rates and PAFs, cardiovascular diseases, 2000–2019

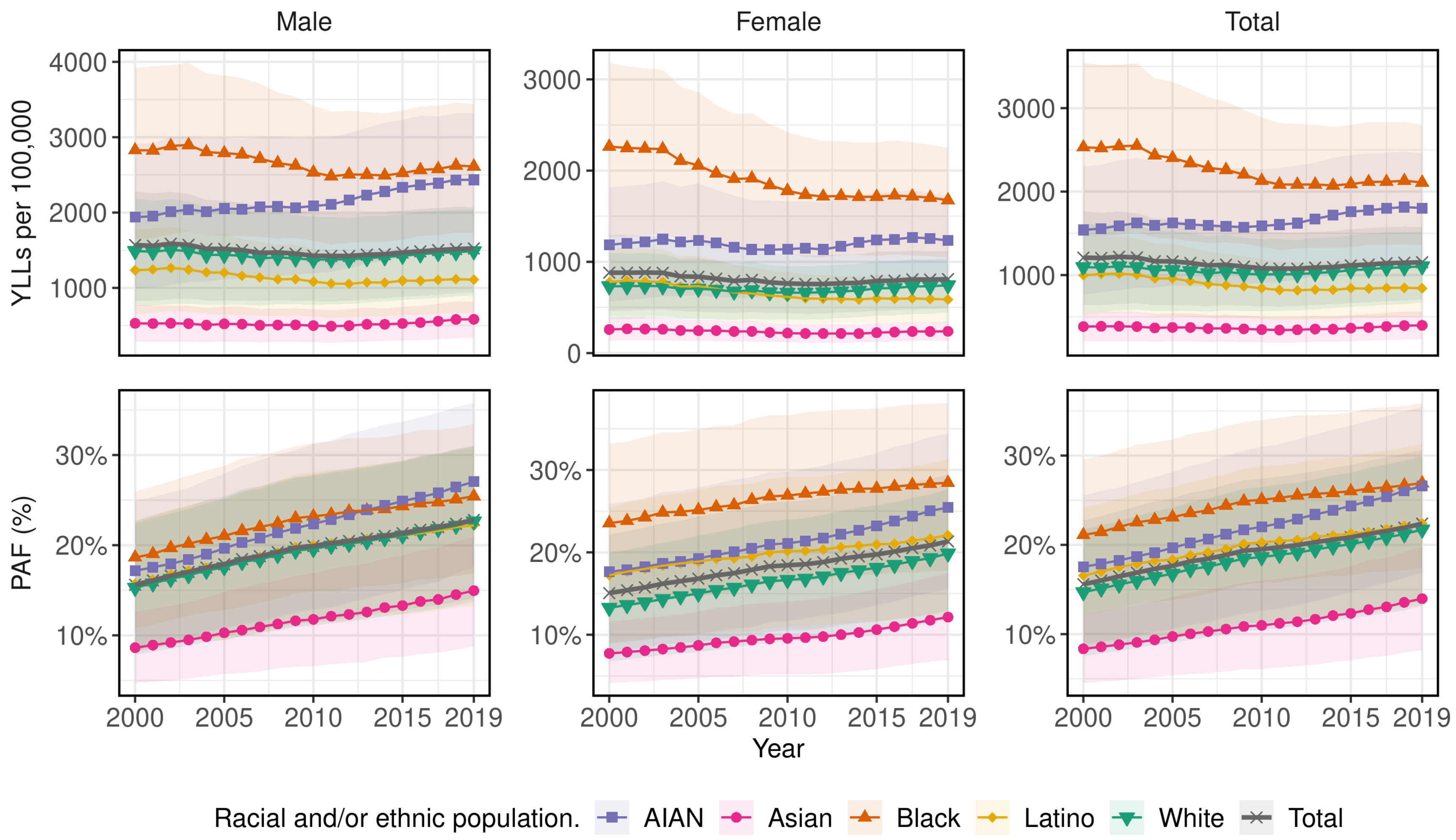

Note: shaded areas indicate the 95% confidence interval

Figure S27. Age-standardised attributable YLL rates and PAFs, ischemic heart disease, 2000–2019

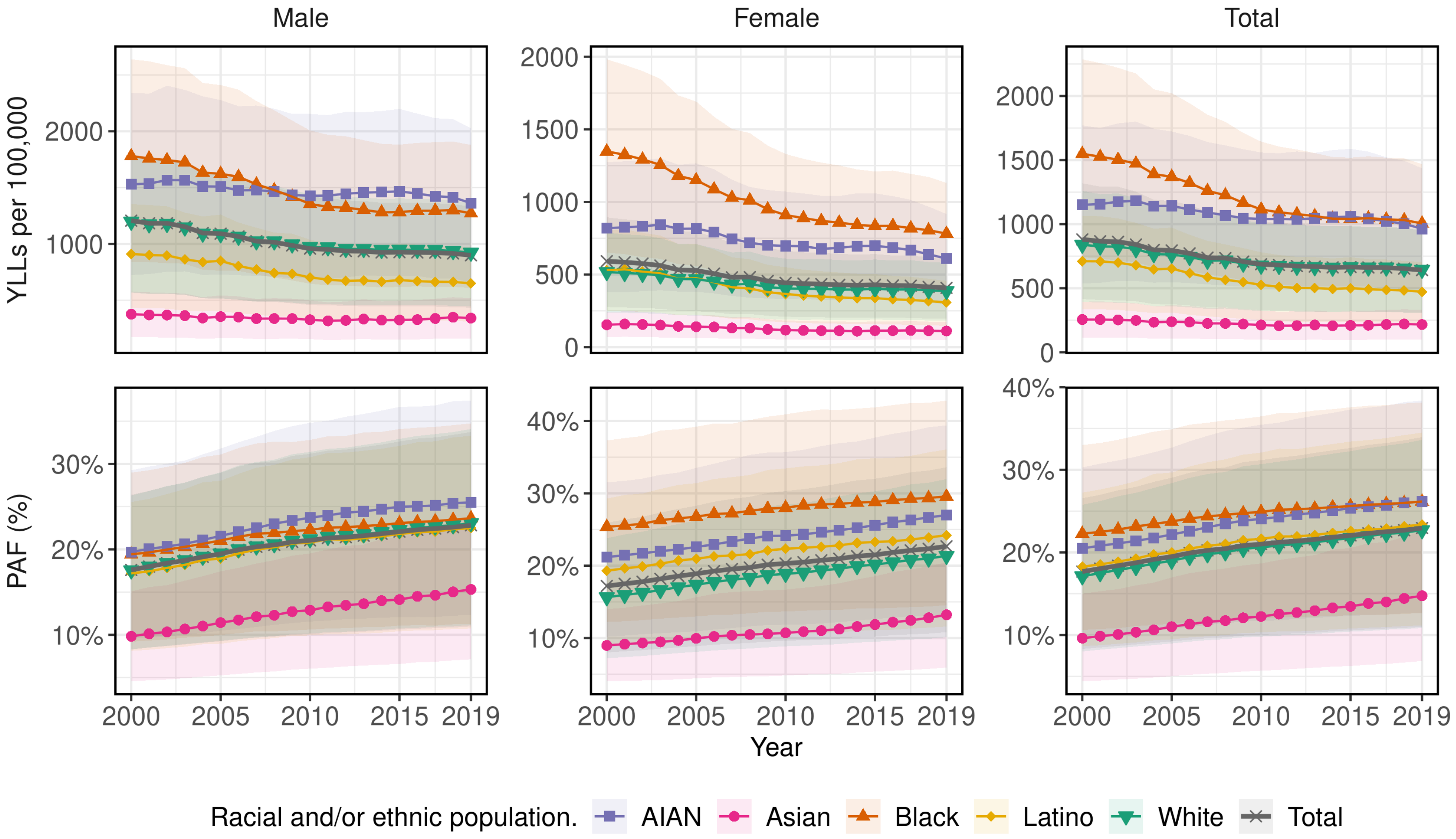

Note: shaded areas indicate the 95% confidence interval

Figure S28. Age-standardised attributable YLL rates and PAFs, stroke, 2000–2019

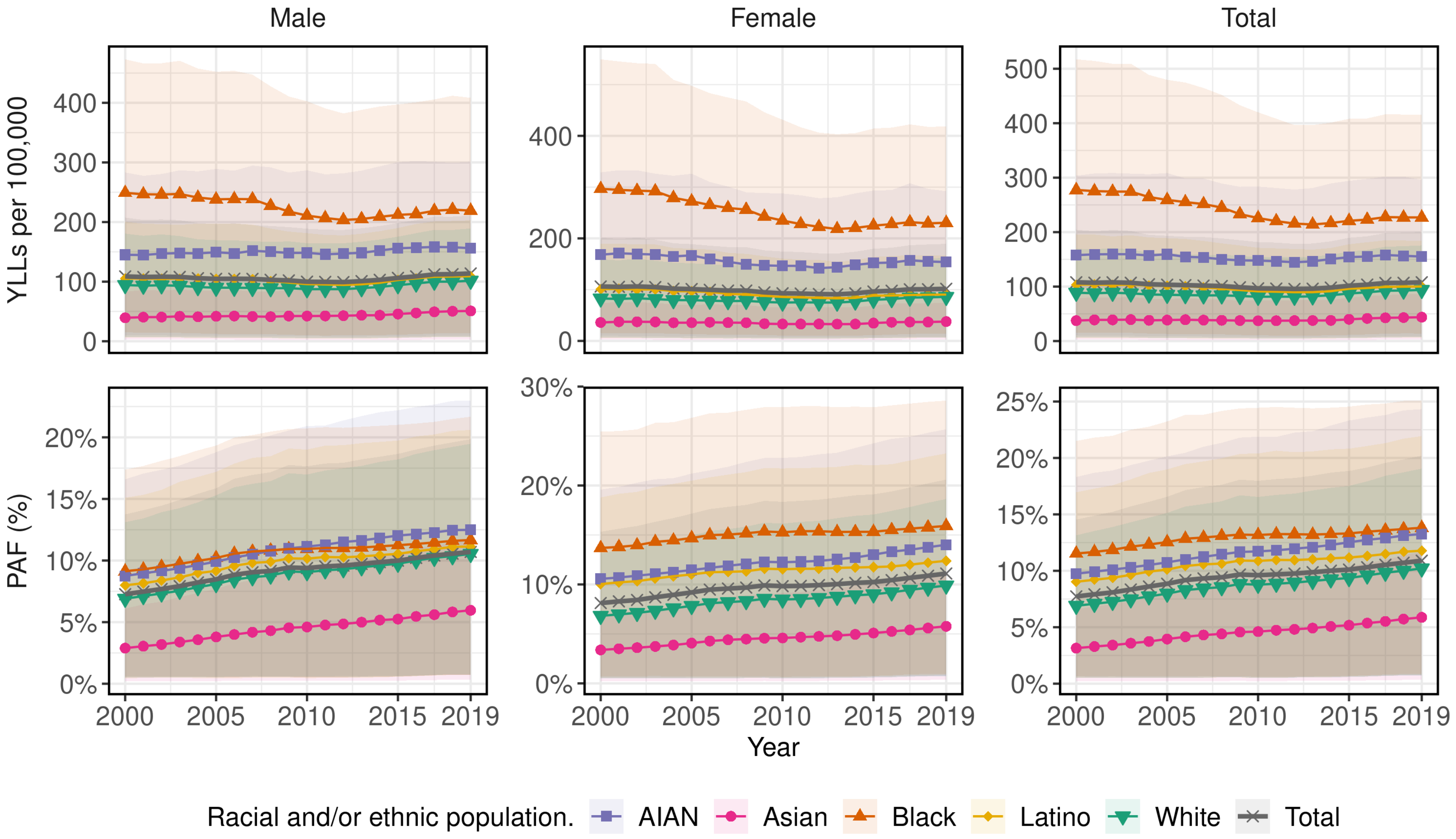

Note: shaded areas indicate the 95% confidence interval

Figure S29. Age-standardised attributable YLL rates and PAFs, ischemic stroke, 2000–2019

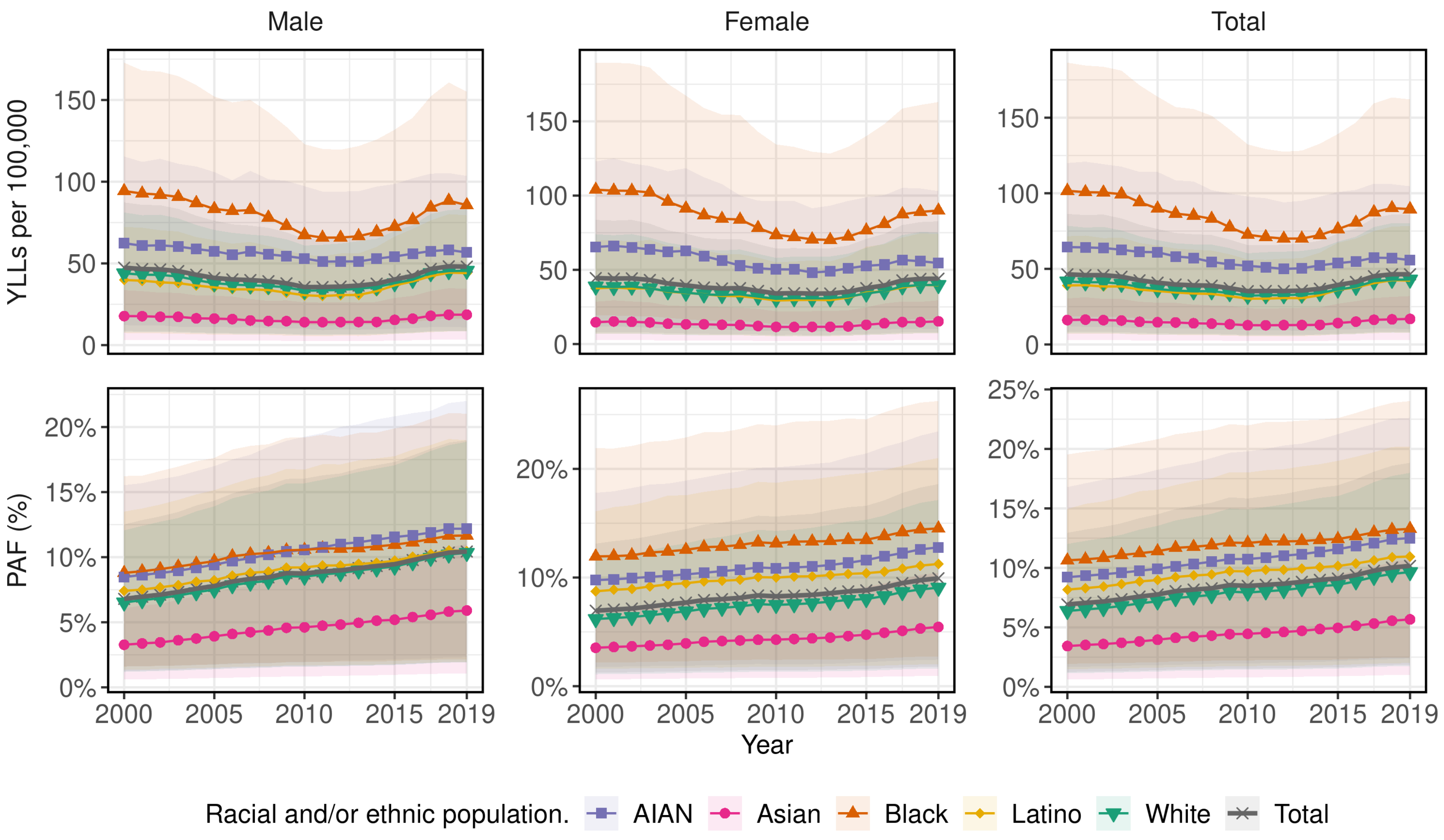

Note: shaded areas indicate the 95% confidence interval

Figure S30. Age-standardised attributable YLL rates and PAFs, intracerebral hem, 2000–2019

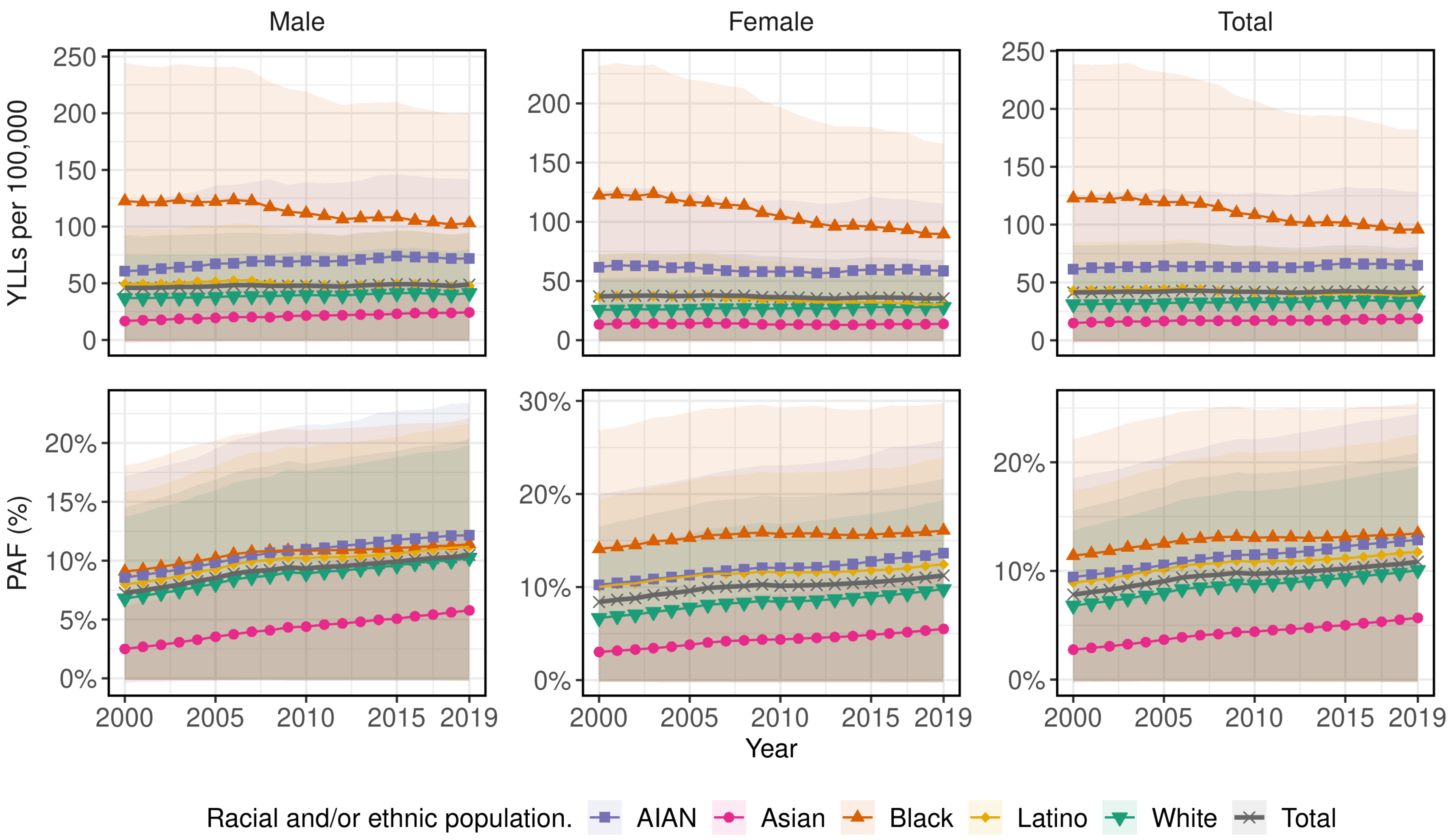

Note: shaded areas indicate the 95% confidence interval

Figure S31. Age-standardised attributable YLL rates and PAFs, subarachnoid hem, 2000–2019

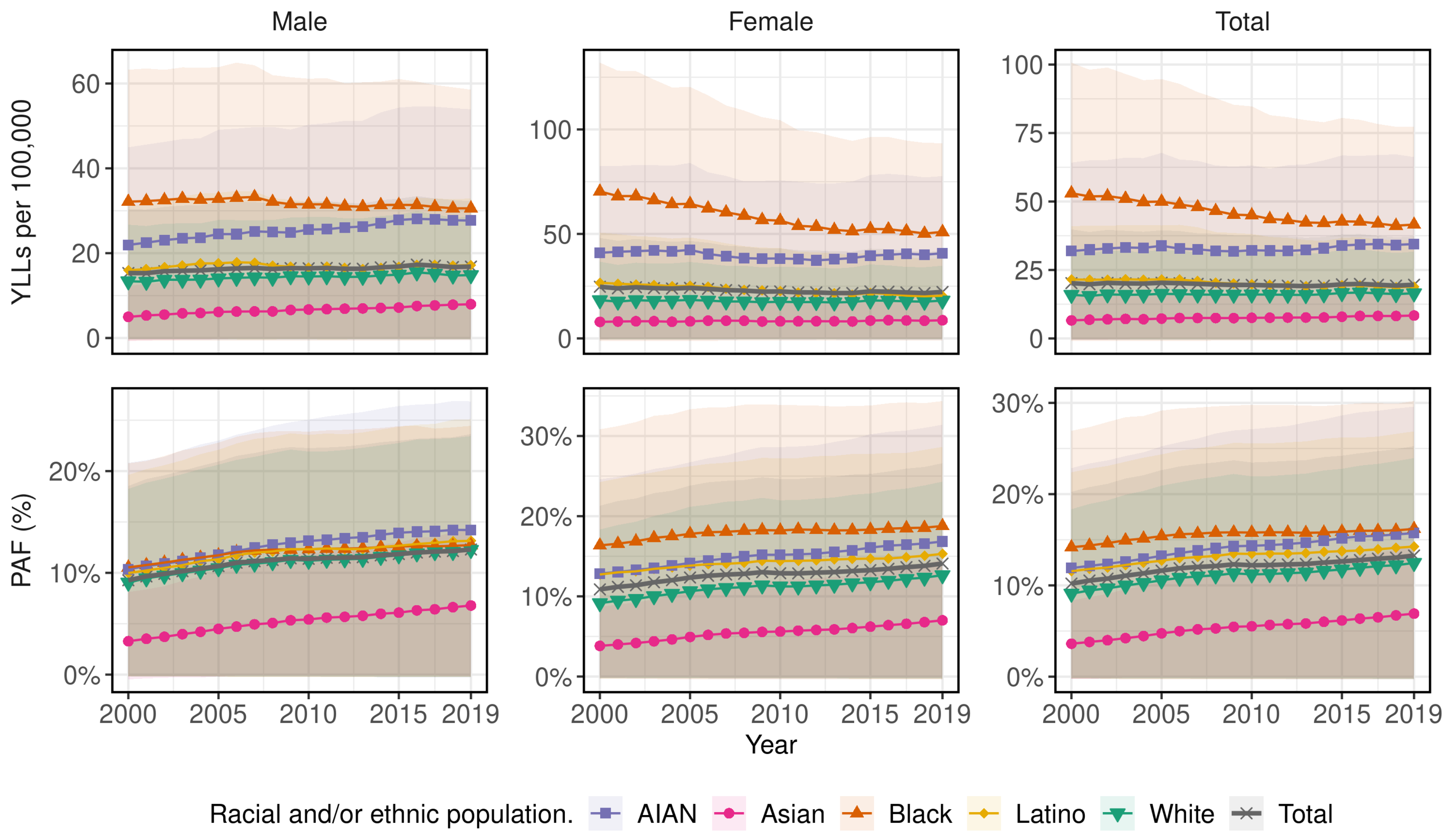

Note: shaded areas indicate the 95% confidence interval

Figure S32. Age-standardised attributable YLL rates and PAFs, hypertensive heart disease, 2000–2019

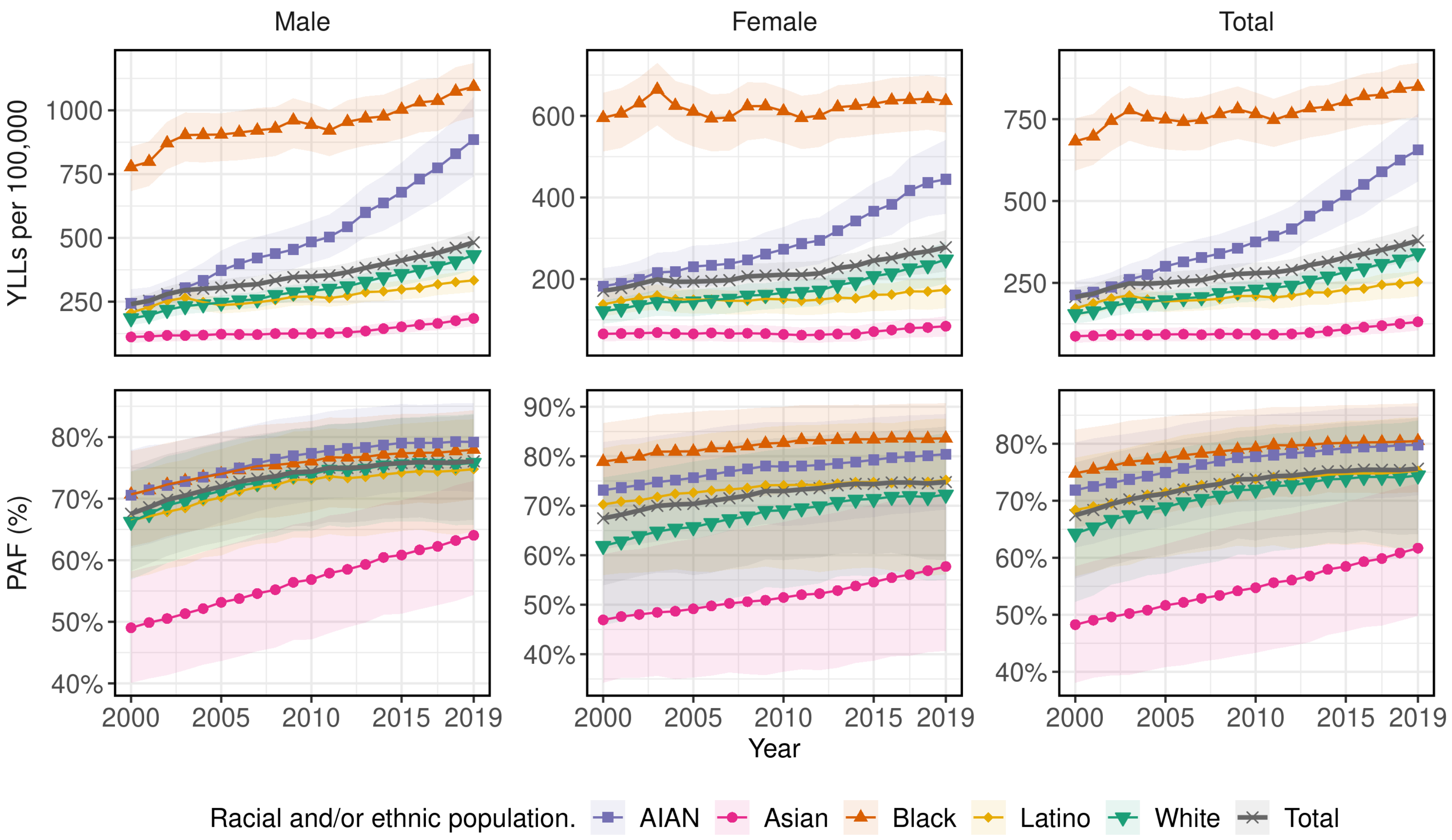

Note: shaded areas indicate the 95% confidence interval

Figure S33. Age-standardised attributable YLL rates and PAFs, atrial fibrillation, 2000–2019

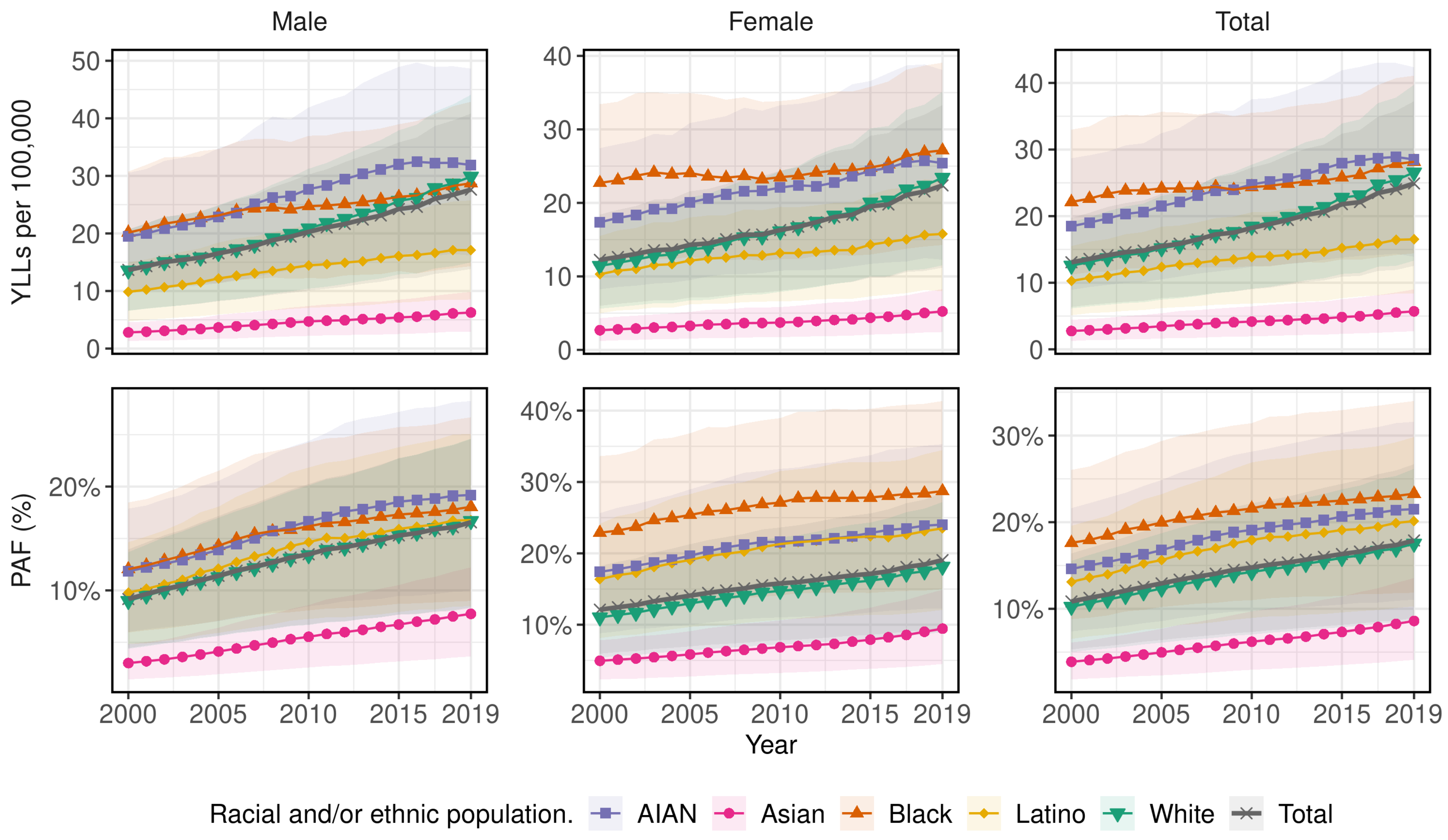

Note: shaded areas indicate the 95% confidence interval

Figure S34. Age-standardised attributable YLL rates and PAFs, chronic respiratory, 2000–2019

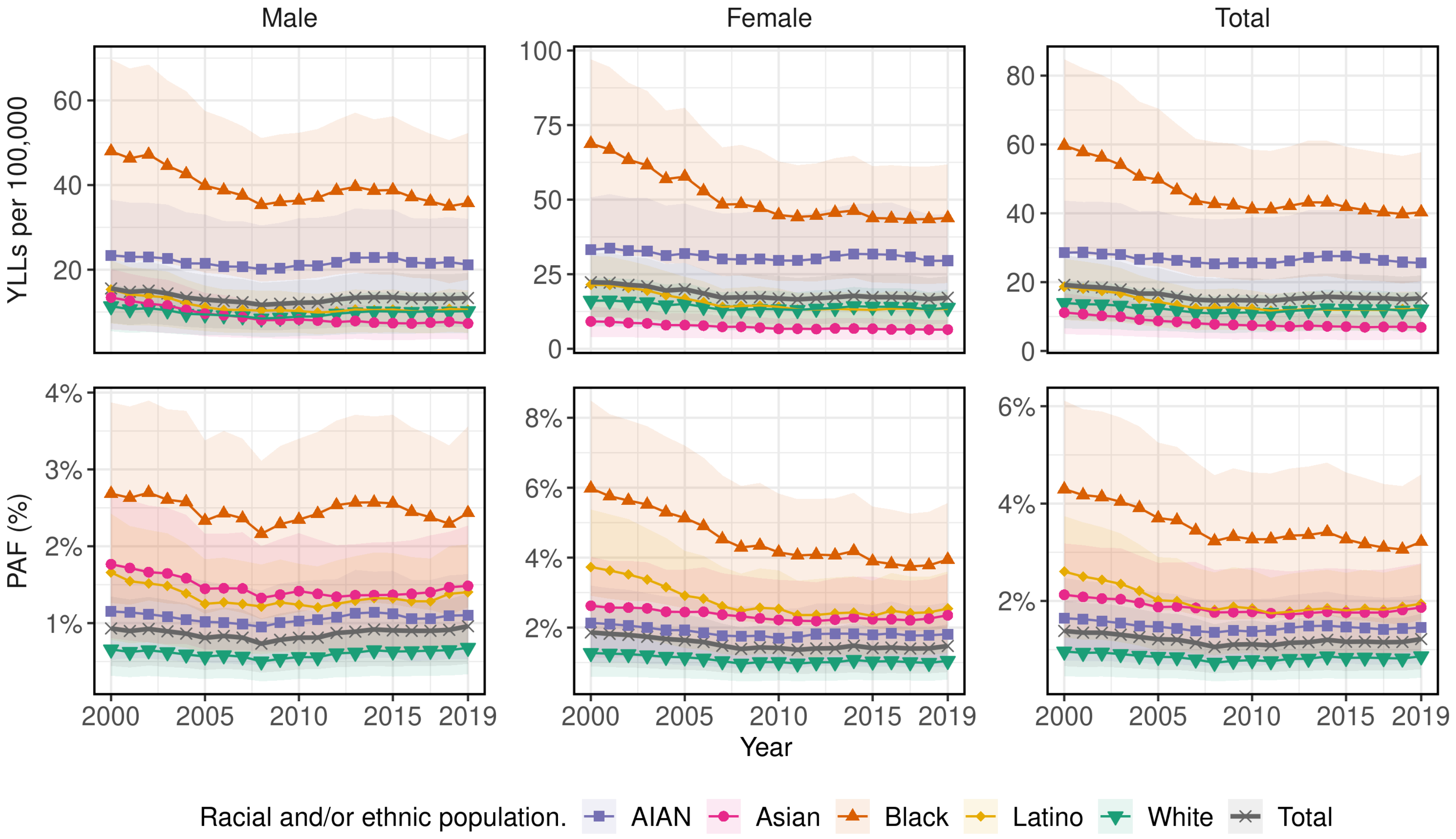

Note: shaded areas indicate the 95% confidence interval

Figure S35. Age-standardised attributable YLL rates and PAFs, asthma, 2000–2019

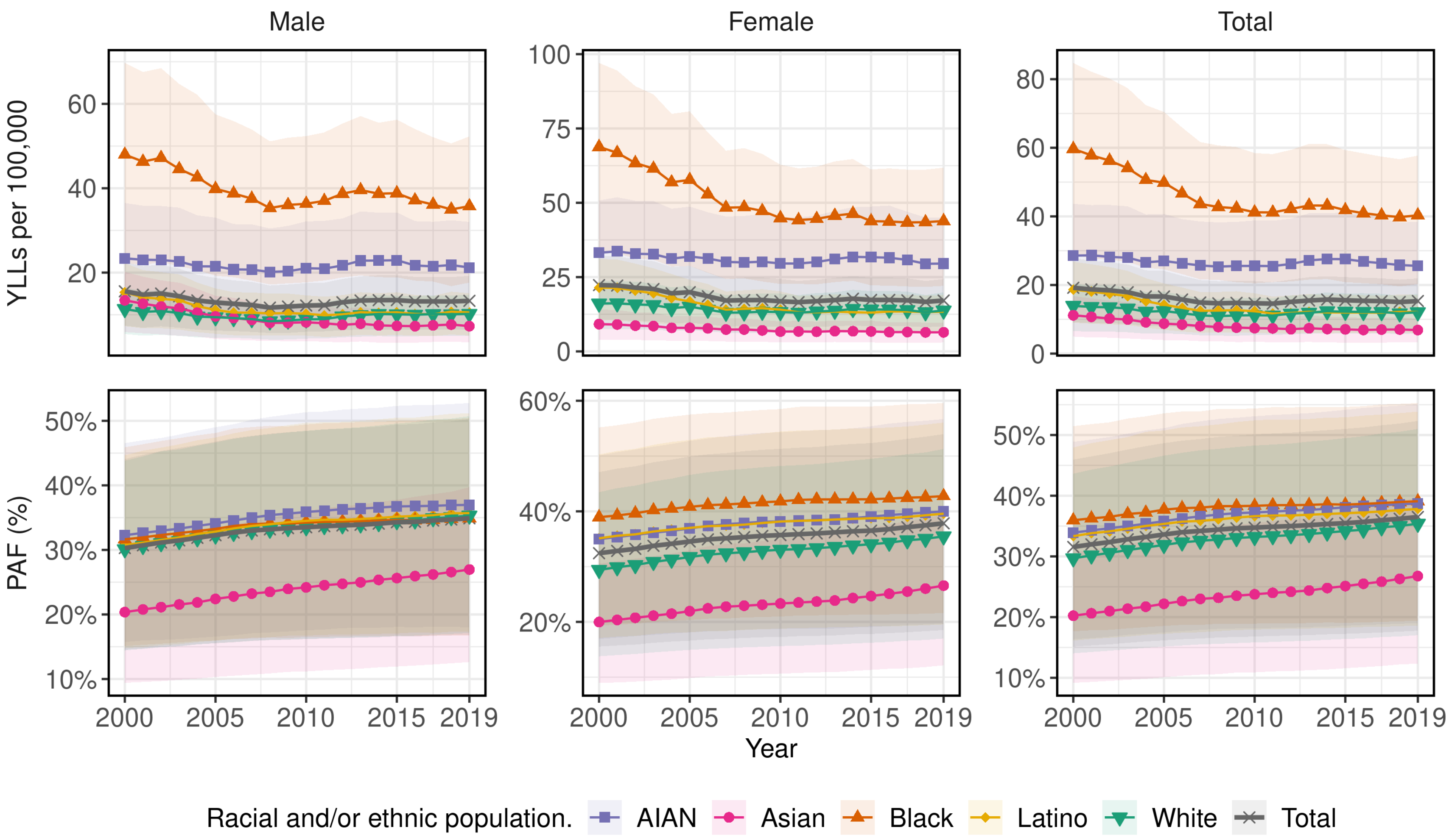

Note: shaded areas indicate the 95% confidence interval

Figure S36. Age-standardised attributable YLL rates and PAFs, digestive diseases, 2000–2019

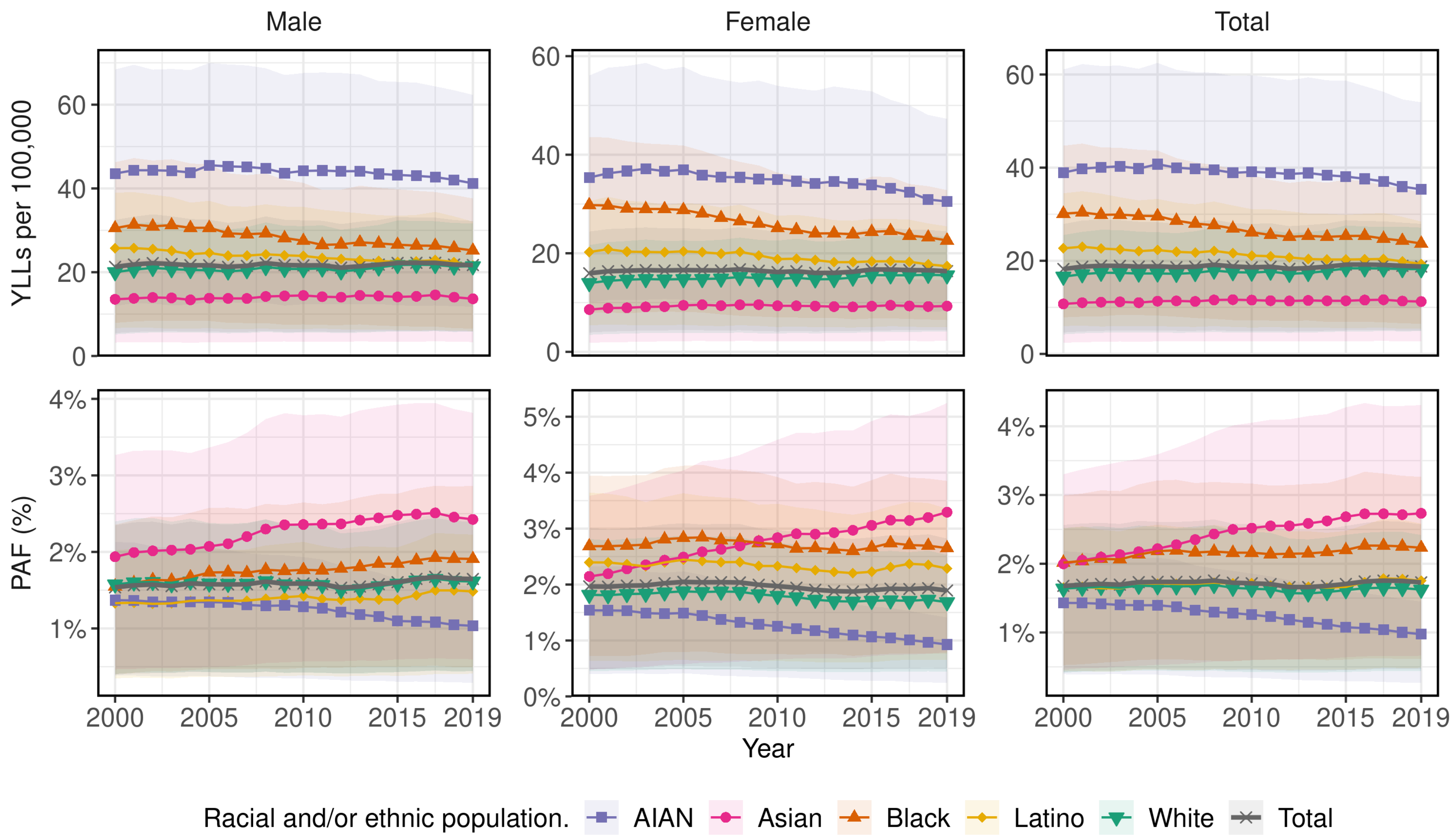

Note: shaded areas indicate the 95% confidence interval

Figure S37. Age-standardised attributable YLL rates and PAFs, gallbladder & biliary, 2000–2019

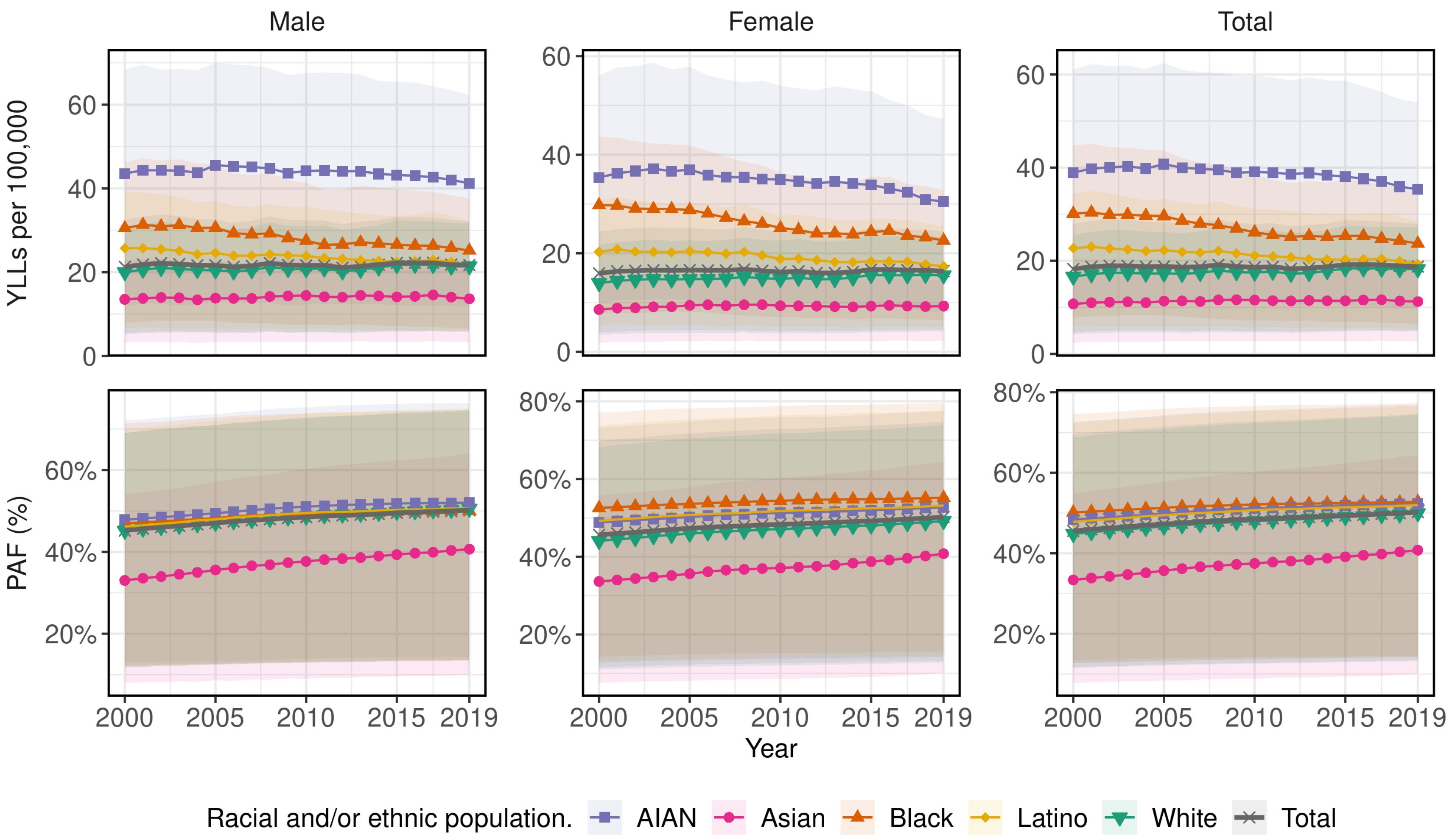

Note: shaded areas indicate the 95% confidence interval

Figure S38. Age-standardised attributable YLL rates and PAFs, neurological disorders, 2000–2019

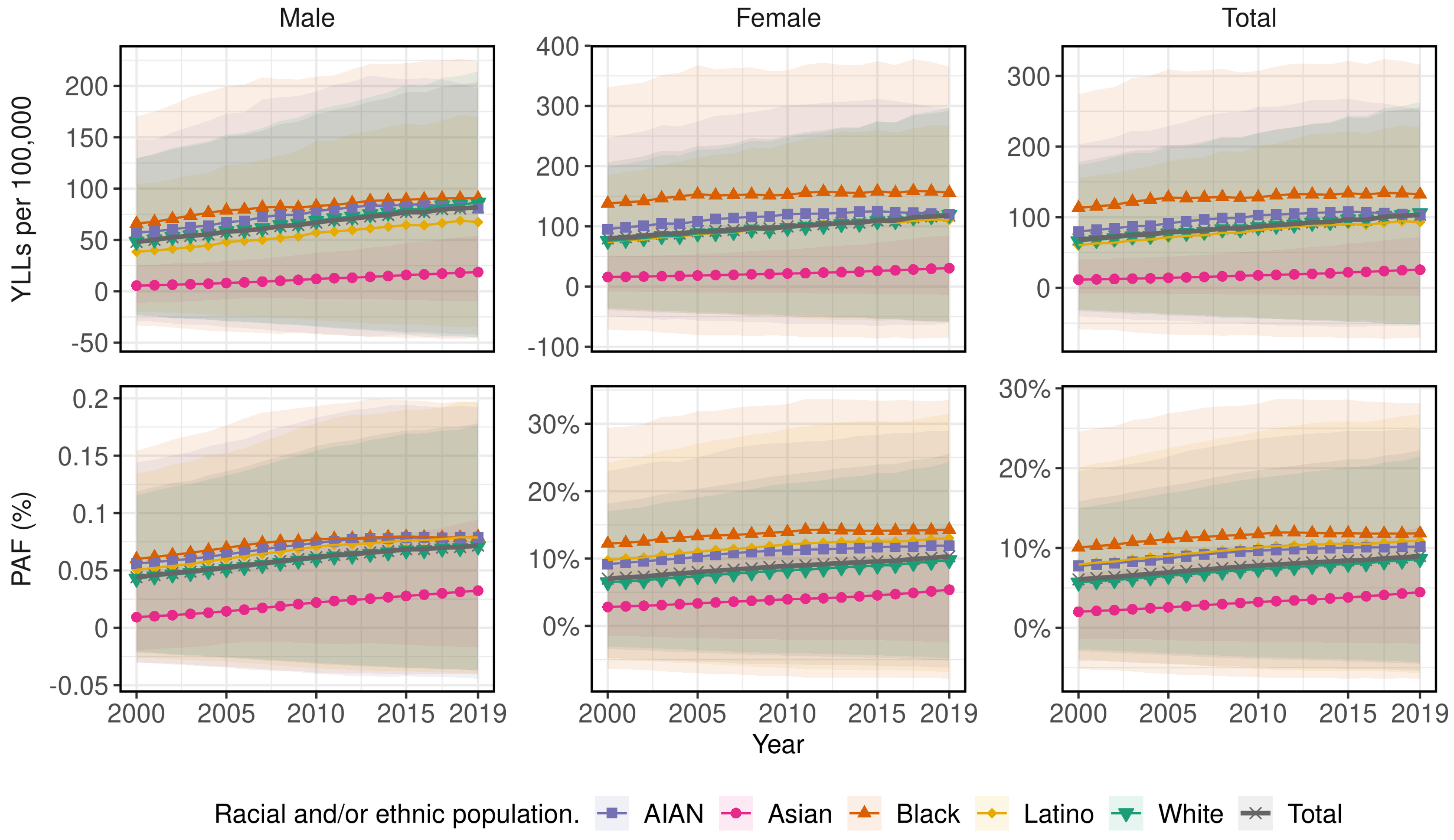

Note: shaded areas indicate the 95% confidence interval

Figure S39. Age-standardised attributable YLL rates and PAFs, Alzheimer's disease, 2000–2019

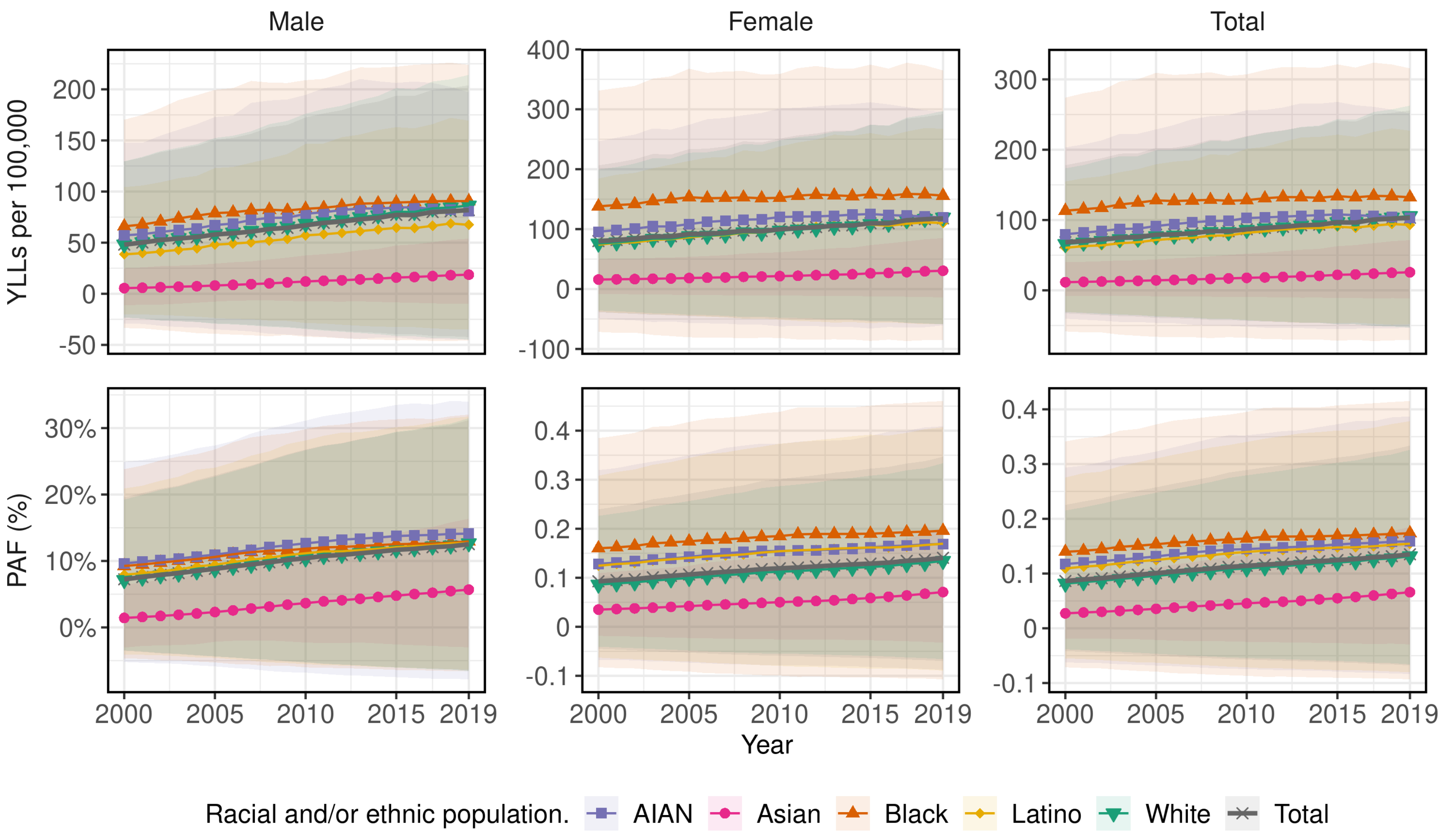

Note: shaded areas indicate the 95% confidence interval

Figure S40. Age-standardised attributable YLL rates and PAFs, diabetes & CKD, 2000–2019

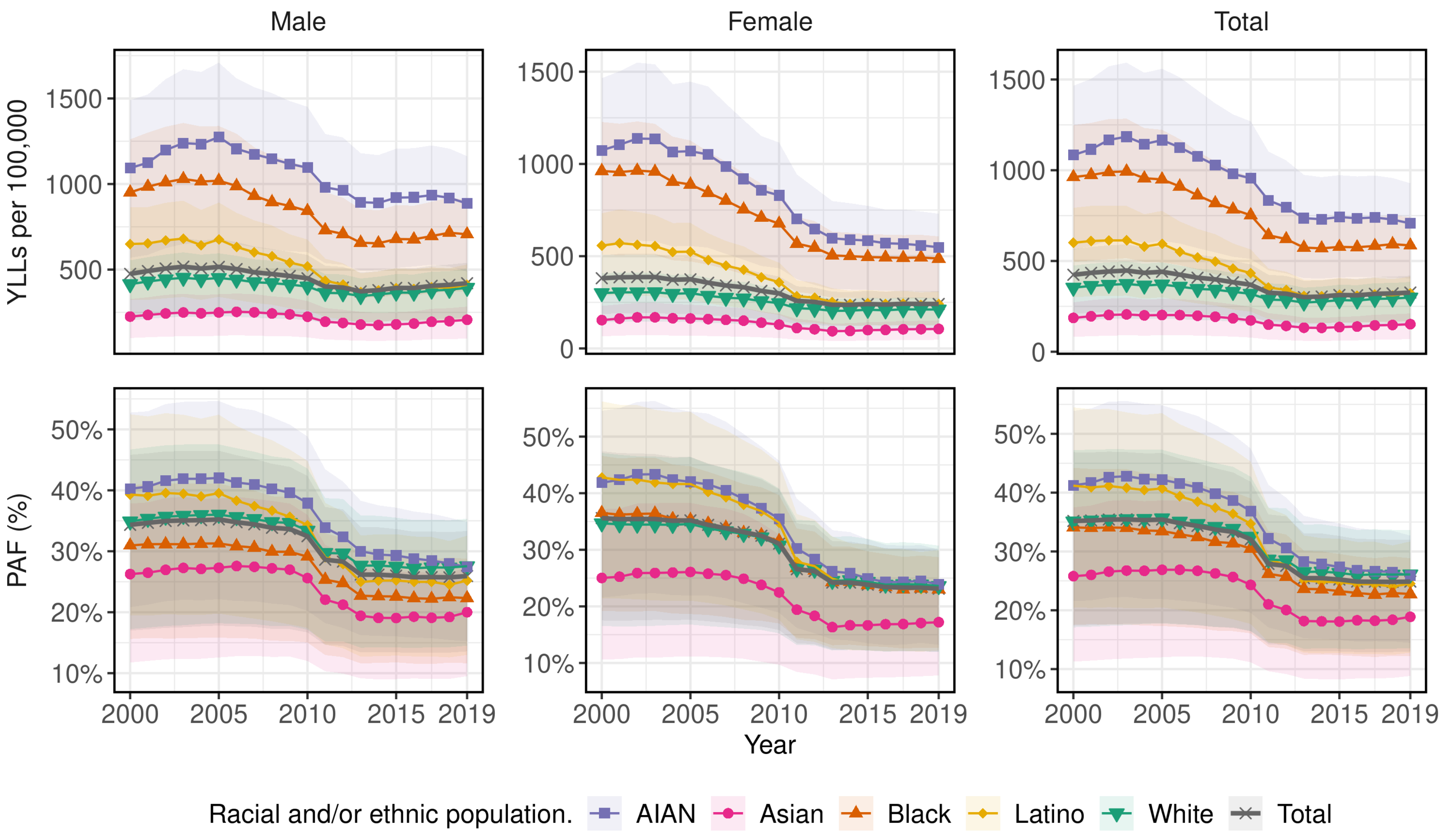

Note: shaded areas indicate the 95% confidence interval

Figure S41. Age-standardised attributable YLL rates and PAFs, diabetes, 2000–2019

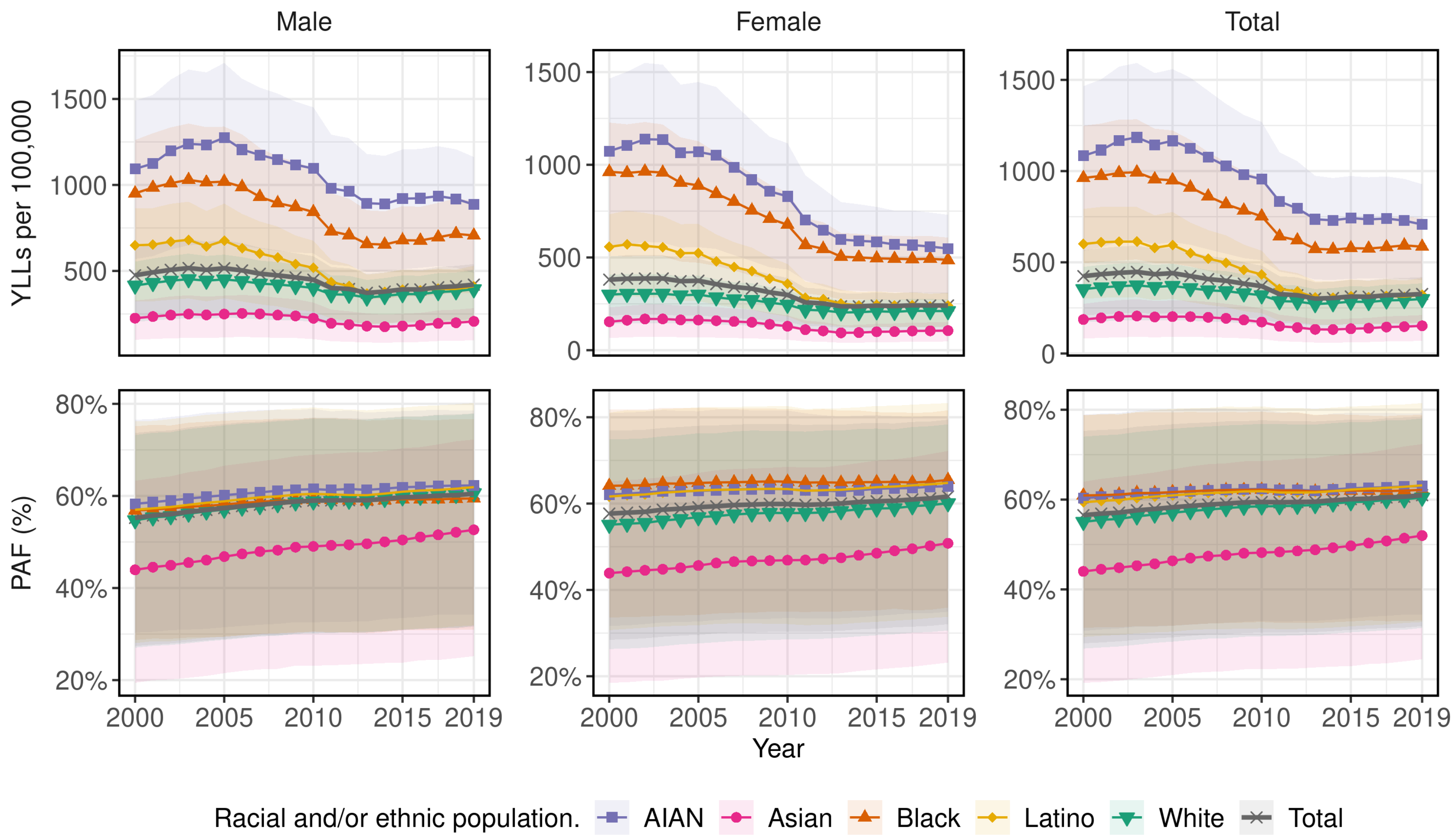

Note: shaded areas indicate the 95% confidence interval

Figure S42. Age-standardised attributable YLL rates and PAFs, diabetes type 2, 2000–2019

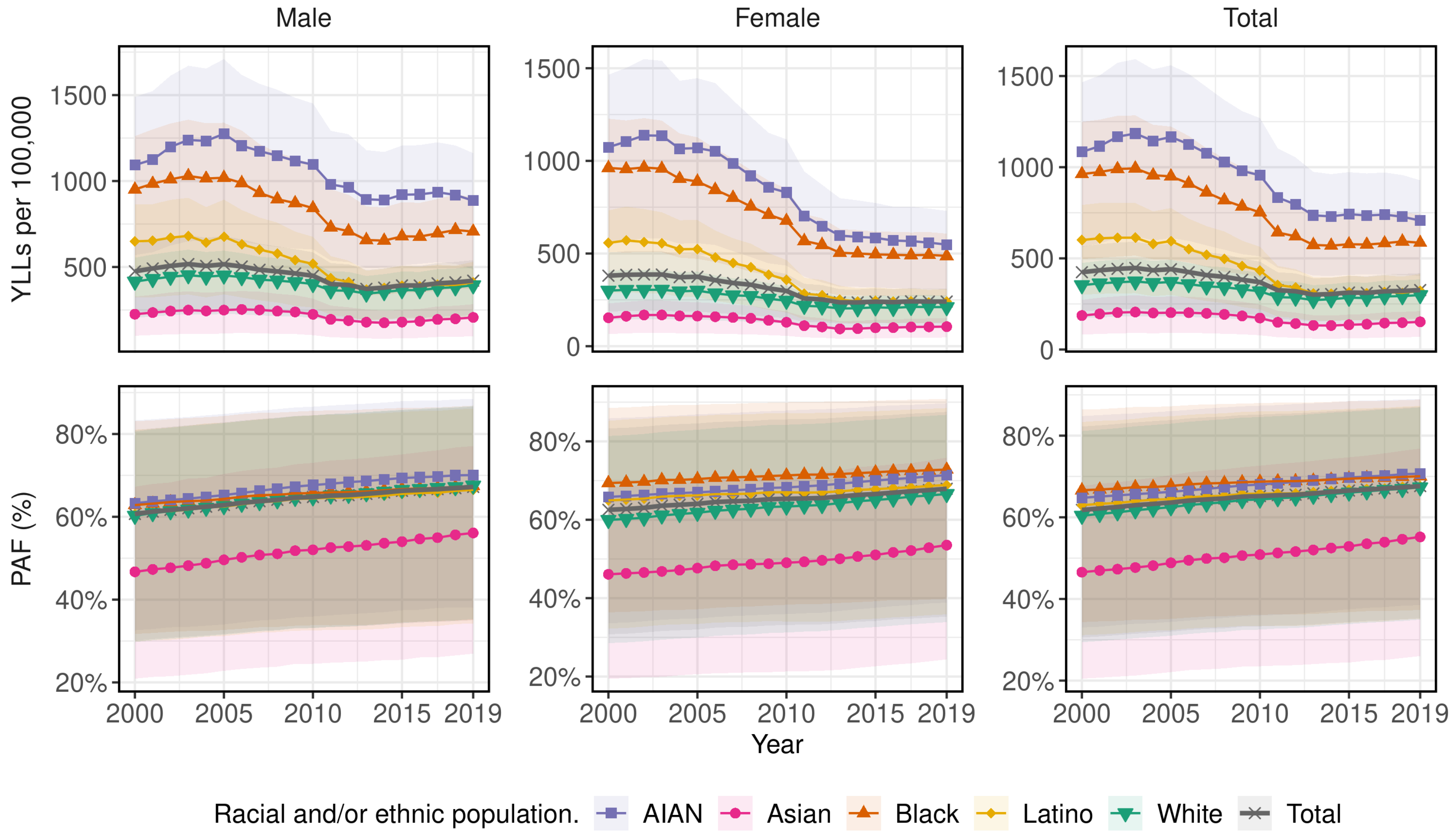

Note: shaded areas indicate the 95% confidence interval
